# Drug resistance in cancer: molecular mechanisms and emerging treatment strategies

**DOI:** 10.1186/s43556-025-00352-w

**Published:** 2025-11-17

**Authors:** Jinxin Li, Jiatao Hu, Yiren Yang, Hanzhong Zhang, Ying Liu, Yu Fang, Le Qu, Anqi Lin, Peng Luo, Aimin Jiang, Linhui Wang

**Affiliations:** 1 Department of Urology, Changhai Hospital, Naval Medical University (Second Military Medical University), Shanghai, China; 2 Department of Urology, Affiliated Jinling Hospital, Medical School of Nanjing University, Nanjing, China; 3 Donghai County People’s Hospital (Affiliated, Donghai County People’s Hospital - Jiangnan University Smart Healthcare Joint Laboratory, Kangda College of Nanjing Medical University), Lianyungang, 222000 China; 4 Department of Oncology, Zhujiang Hospital, Southern Medical University, Guangzhou, 510282 Guangdong China

**Keywords:** Drug resistance, Cancer, Tumor microenvironment, Multi-omics, Therapeutic strategies

## Abstract

Therapeutic resistance remains a defining challenge in oncology, limiting the durability of current therapies and contributing to disease relapse and poor patient outcomes. This review systematically integrates recent progress in understanding the molecular, cellular, and ecological foundations of drug resistance across chemotherapy, targeted therapy, and immunotherapy. We delineate how genetic alterations, epigenetic reprogramming, post-translational modifications, and non-coding RNA networks cooperate with metabolic reprogramming and tumor microenvironment remodeling to sustain resistant phenotypes. The influence of the microbiome is highlighted as an emerging determinant of therapeutic response through immune modulation and metabolic cross-talk. By summarizing key regulatory circuits, We establishe a unified framework linking clonal evolution, metabolic adaptability, and tumor ecological dynamics. We further synthesizes novel therapeutic strategies that convert resistance mechanisms into therapeutic vulnerabilities, including synthetic lethality approaches, metabolic targeting, and disruption of stem cell and stromal niches. Advances in single-cell and spatial omics, liquid biopsy, and artificial intelligence are emphasized as transformative tools for early detection and real-time prediction of resistance evolution. This review also identifies major translational gaps in preclinical modeling and proposes precision oncology frameworks guided by evolutionary principles. By bridging mechanistic understanding with adaptive clinical design, this work provides an integrated roadmap for overcoming therapeutic resistance and achieving sustained, long-term cancer control.

## Introduction

Cancer has become one of the leading causes of mortality worldwide. In 2022, an estimated 20 million new cancer cases were reported globally, with approximately 9.7 million deaths attributed to cancer and its complications [1]. This imposes not only a significant public health burden but also a major threat to human life expectancy [2]. For the vast majority of solid tumors, curative resection remains the primary treatment option [3]. However, for certain early-stage tumors, particularly those with no obvious clinical symptoms and high invasiveness, it is often challenging to achieve early detection and timely intervention, resulting in the loss of the optimal treatment window [4].

To address this challenge, comprehensive treatment strategies centered on drug therapy have become the cornerstone of management for most tumors. From traditional chemotherapy to targeted therapy and immunotherapy, the spectrum of available treatments has continued to expand, leading to significant reductions in mortality rates and improvements in overall survival [5–7]. However, drug resistance, the most fundamental challenge in cancer treatment, is directly and fatally linked to tumors. It is not an isolated complication but a core biological process that causes treatment failure and triggers tumor recurrence and metastasis. The complexity of this issue stems from the extreme diversity of its mechanisms [8]. Tumor cells can evade killing through multiple pathways, including activating drug efflux pumps, inducing target mutations, and activating alternative signaling pathways [9–11]. Moreover, these mechanisms exhibit spatial heterogeneity within tumors and dynamically evolve over the course of treatment.

More alarmingly, the prevalence of drug resistance extends across all mainstream therapies. From the perspective of drug classes, whether chemotherapy agents, targeted therapies, immunotherapeutic agents, or novel modalities such as (ADCs, resistance remains a major concern [12–15]. From the perspective of treatment timelines, resistance may present as either primary (intrinsic) insensitivity or as acquired resistance developing during the course of therapy [16, 17]. Regardless of type, all forms of resistance ultimately lead to tumor recurrence or metastasis, culminating in treatment failure. Therefore, the early identification of resistance types and elucidation of their molecular mechanisms are crucial for optimizing therapeutic strategies and improving clinical outcomes.

This review begins with the current clinical landscape of cancer treatment and examines the various forms of therapeutic resistance, including both intrinsic and acquired types. It further analyzes resistance across different treatment modalities. Based on this framework, we provide a detailed synthesis of resistance mechanisms at multiple biological levels, including gene expression and mutation, epigenetic modifications, protein expression and post-translational modifications, and tumor immune microenvironmental factors. More importantly, we systematically summarize emerging strategies proposed in recent years to overcome drug resistance, with a particular focus on novel therapies. Finally, we highlight limitations in current research and propose future directions that may guide subsequent studies. Through this synthesis and analysis, we aim to provide a valuable reference for elucidating the molecular basis of tumor drug resistance and for developing more effective treatment strategies.

## Clinical burden and evolving resistance paradigms

Tumor drug resistance fundamentally limits the clinical benefits of cancer therapy. Currently, approximately 90% of chemotherapy failures and more than 50% of targeted or immunotherapy failures are directly attributable to resistance, which not only impedes improvements in survival rates but also results in substantial waste of medical resources [18]. As precision medicine guided by molecular subtyping advances, the dual paradigm of resistance, intrinsic versus acquired, along with tumor-specific barriers, has become a critical consideration in clinical decision-making. The unique resistance mechanisms associated with targeted therapies, immune checkpoint inhibitors, and ADCs further underscore the dynamic evolutionary capacity of tumors under therapeutic pressure. Therefore, by assessing the hierarchical clinical burden imposed by resistance, deconstructing its taxonomic framework, and elucidating the molecular logic underlying resistance in these three leading therapeutic modalities, a mechanistic roadmap can be established to help overcome current treatment bottlenecks.

### Therapeutic failure in practice

In clinical practice, tumor resistance has become a core challenge in cancer management. The emergence of resistance is the principal cause of most tumor recurrences and a major factor contributing to cancer-related mortality [19]. Importantly, this challenge is not confined to any single treatment type but is observed across virtually all malignancies [20]. Resistance is thus a universal obstacle faced by clinicians and researchers alike.

Among available therapeutic options, resistance has been documented in both conventional chemotherapy and newer modalities such as targeted therapy and immunotherapy. Reports indicate that up to 90% of chemotherapy failures are attributable to resistance, a finding consistently observed across most malignant tumors [18]. In breast cancer, resistance to paclitaxel [21], 5-fluorouracil [22], doxorubicin [23], cyclophosphamide [24], and carboplatin [25] not only results in tumor recurrence but also predicts poor patient prognosis. Similarly, in colorectal and gastric cancers, resistance to agents such as 5-fluorouracil [26, 27], and oxaliplatin [28, 29] poses a serious threat to patient survival. Targeted agents and immune checkpoint inhibitors, including small molecules and biologics, have demonstrated significant efficacy but remain subject to resistance. For example, imatinib mesylate, widely recognized for its success in chronic myeloid leukemia and gastrointestinal stromal tumors, eventually encounters resistance [30]. Targeted therapies against human epidermal growth factor receptor 2 (HER2) can develop resistance within one year [31]. In melanoma, immunotherapy resistance can occur within five years, and in non–small cell lung cancer (NSCLC), disease progression occurs in approximately 56% of patients within four years [32].

To address resistance to monotherapy, combination strategies have been proposed. Theoretically, due to differing mechanisms of action and resistance, combination therapy should maximize efficacy while minimizing the likelihood of resistance. However, in clinical reality, combination regimens often fail, particularly in advanced-stage tumors, because of emerging resistance. Moreover, while response rates may improve, multidrug chemotherapy significantly increases treatment-related toxicities [33]. This challenge extends beyond chemotherapy to combinations involving targeted therapies and immunotherapies. For instance, in metastatic melanoma, dual immune checkpoint blockade with anti–PD-1 and anti–CTLA-4 antibodies achieved high initial response rates, but long-term survival benefits remain uncertain, with nearly half of patients experiencing severe treatment-related adverse events [34]. Given the existence of cross-resistance and multidrug resistance mechanisms, combination therapy does not represent a definitive solution to the problem [35].

### Clinical categorization of drug resistance

**Fig. 1:**
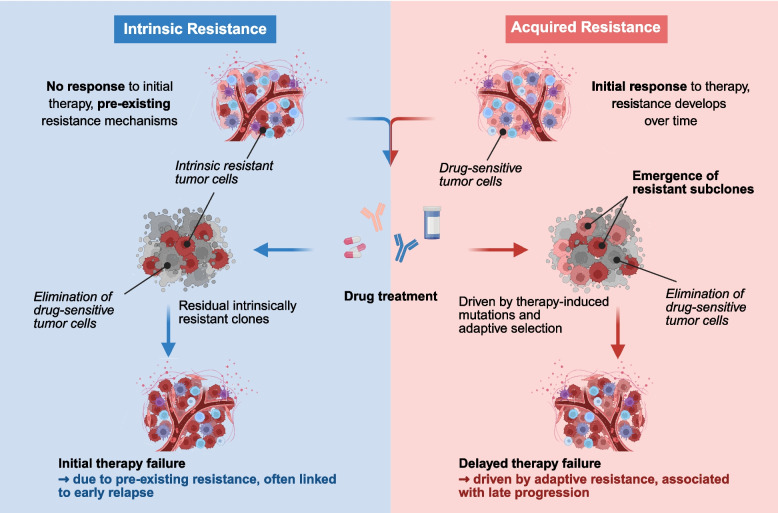
The evolution of intrinsic and acquired drug resistance

Broadly, drug resistance can be classified into two paradigms: intrinsic resistance (primary resistance) and acquired resistance (secondary resistance) (Fig. 1). Intrinsic resistance refers to a lack of response to initial treatment, indicating that resistance mechanisms pre-exist before therapy begins [16]. Acquired resistance develops during or after treatment, implying that there was an initial therapeutic response followed by the emergence of resistance [36]. This therapeutic escape severely compromises the achievement of complete remission.

Tumor cells possess remarkable phenotypic plasticity, enabling survival through continuous adaptation under immune surveillance and anticancer therapy. During phenotypic conversion, certain tumor cells undergo Darwinian passive selection and enrichment, ensuring the survival of the fittest, while others actively respond to diverse internal and external stimuli [37]. Under the combined pressures of tumor heterogeneity and drug selection, a minority of tumor cells divide and form distinct subpopulations [38, 39]. These resistant subclones often exhibit dormancy and stem cell–like properties, proliferate slowly, and ultimately result in persistent resistance leading to disease progression. In some cases, they may regain drug sensitivity after treatment cessation [40].

In addition to these general categories, tumor-specific resistance mechanisms pose unique challenges. For example, molecular heterogeneity often drives resistance. In NSCLC, the epidermal growth factor receptor (EGFR) is a well-established therapeutic target. Despite mutation rates of up to 30%, three generations of EGFR tyrosine kinase inhibitors (EGFR-TKIs) have been developed for this patient population, yet resistance inevitably emerges [41]. First-generation EGFR-TKIs such as gefitinib and second-generation agents such as afatinib are effective in patients with and L858R mutations. However, the emergence of the T790M mutation leads to acquired resistance in a substantial proportion of patients within 9–14 months of treatment [42, 43]. Third-generation EGFR-TKIs, exemplified by osimertinib, display improved efficacy against T790M-positive tumors but have driven the appearance of the C797S mutation. Consequently, resistance can arise via both EGFR-dependent and EGFR-independent pathways [44–46].

Microenvironmental characteristics also play pivotal roles in resistance. In pancreatic ductal adenocarcinoma (PDAC), the acellular matrix can constitute up to 90% of tumor volume, displaying extensive fibrosis [47]. Excess extracellular matrix (ECM) deposition elevates interstitial fluid pressure, impairs vascularization, and, when driven by activated cancer-associated fibroblasts (CAFs), creates a physical barrier to drug delivery [48, 49]. Such features significantly limit the penetration of agents like gemcitabine and are associated with poor prognosis. In glioblastoma, vascular abnormalities may disrupt the blood–brain barrier (BBB) and increase permeability; however, this disruption is uneven, and many regions maintain an intact BBB [50]. Moreover, overexpression of efflux pumps further reduces drug concentrations, resulting in diminished therapeutic efficacy [51].

Hematological malignancies, unlike solid tumors, are not impeded by physical barriers or dense ECM but depend on specialized mechanisms such as stem cell dormancy and bone marrow niche dynamics. In chronic myeloid leukemia (CML), the BCR–ABL fusion protein is a classical target for TKI therapy with imatinib [52]. Nonetheless, mutations in SH3 and SH2 kinase domains, alterations in the P-loop via the T315I mutation, and single amino acid substitutions can substantially impair TKI efficacy [53]. In multiple myeloma (MM), which arises from plasma cells, the bone marrow microenvironment (BMME) plays a central role [54]. Interactions between immune cells and MM cells, through both direct contact and soluble mediators, activate multiple signaling pathways, enhancing tumor survival, reducing drug sensitivity, and fostering resistance [55].

Addressing the spatial heterogeneity of tumors, barriers to microenvironmental remodeling, and tumor type–specific resistance mechanisms is essential for establishing a multidimensional understanding of drug resistance and advancing therapeutic strategies.

### Resistance in targeted therapy, immunotherapy, and antibody–drug conjugates

Targeted therapy is currently one of the main comprehensive treatment approaches for diverse malignancies. To date, numerous molecular targets across multiple signaling pathways have been identified and applied in clinical practice, yet drug resistance remains an inevitable obstacle. Broadly, the evolution of resistance to targeted therapy involves both target-dependent and target-independent mechanisms (Table 1). The former primarily includes target modifications (mutations, amplifications, fusions) and bypass activation (upregulation of compensatory signaling pathways), whereas the latter encompasses phenotypic conversion (lineage plasticity) and microenvironmental protection (physical barriers, immune suppression), among others.

**Table 1:** The main targets of targeted therapy and immunotherapy and their mechanisms of resistance

Signaling pathway	Target	Representative drugs	Main mechanisms of resistance	References
RTKs pathway	EGFR	Osimertinib	Secondary mutations (T790M/C797S) MET/HER2 amplification phenotypic transformation	[56]
	ALK	Lorlatinib	ALK kinase-domain mutations Activation of bypass signalling pathways	[57]
	ROS1	Crizotinib	Secondary mutations (G2032R/L2026M) ROS1 fusion	[58]
	MET	Capmatinib	Secondary mutations (D1228/Y1230) EGFR compensatory activation	[59]
	HER2	Trastuzumab	Intracellular domain truncation (p95-HER2) PI3K/AKT sustained activation	[60]
MAPK pathway	KRAS ^G12C^	Sotorasib	Acquired Y96D/H95Q mutation NRAS/AKT feedback activation	[61]
	MEK	Trametinib	Acquired mutations in MEK1/2 ERK reactivation	[62]
Angiogenesis pathway	VEGFR	Bevacizumab	Alternative vascular growth pathway (FGF) Hypoxia-induced VEGF expression	[63]
	HIF-2α	Belzutifan	Compensatory activation of HIF-1α Acquired mutations in EPAS1	[64]
DDR pathway	PARP	Olaparib	Restoration of BRCA1/2 function 53BP1 deletion Overexpression of drug efflux pump (ABCB1)	[65]
	ATM/ATR	AZD1390	Upregulation of replication fork protection protein	[66]
DDR pathway	Polθ	RP-6685	53BP1 deletion	[66]
Epigenetics pathway	IDH1/2	Enasidenib Ivosidenib	Acquired IDH2 mutation (Q316E) 2-HG metabolic bypass activation	[67]
	BET	RVX208	BRD4 hyperphosphorylation Enhanced Wnt/β-catenin signaling	[68]
Cell cycle pathway	CDK4/6	Palbociclib	RB1 deletion/mutation CDK2/cyclin E amplification FGFR1 signal compensation	[69]
immune checkpoint	PD1/PDL1	Nivolumab Avelumab	T Cell Exhaustion Disfunction of tumor antigen Compensatory Inhibitory Signaling	[70]

*RTKs *Receptor tyrosine kinases, *MAPK *Mitogen-activated protein kinase, *DDR *DNA damage repair

For example, the therapeutic efficacy of trastuzumab, which targets HER2, is influenced not only by expression levels of the p95-HER2 isoform but also by mutations in phosphatidylinositide 3-kinase (PI3K) [71]. Alterations in PI3K catalytic subunit alpha (PIK3CA) or loss of phosphatase and tensin homolog (PTEN) can hyperactivate the PI3K–AKT pathway, thereby promoting resistance to anti-HER2 therapy [72]. Due to the complexity of intracellular signaling and regulatory networks, inhibition of a single target often triggers feedback loops that establish new compensatory pathways, fostering resistance. For instance, capmatinib, a MET inhibitor used in NSCLC and gastric cancer, can induce activation of the EGFR pathway, leading to resistance [73–75].

Immune suppression can also undermine targeted therapy. In tumors resistant to osimertinib, increased macrophage infiltration, heightened M2 polarization, reduced T cell infiltration, and diminished activation collectively create an immunosuppressive microenvironment that compromises therapeutic efficacy [76]. Thus, resistance to targeted therapy reflects a multifaceted process involving molecular alterations, signaling pathway reprogramming, and tumor microenvironmental modulation, underscoring the need for integrative perspectives to decipher its underlying logic.

Immunotherapy likewise demonstrates both primary and secondary resistance. Primary resistance frequently occurs in so-called “cold tumors”, characterized by low antigenicity, minimal or absent T cell infiltration, and an immunosuppressive microenvironment, resulting in poor responsiveness [77]. For example, prostate cancer is considered an “immune desert” with low T cell infiltration and weak immunogenicity, severely limiting the efficacy of immune checkpoint inhibitors [78, 79]. Even “hot tumors” are not uniformly responsive. In renal cell carcinoma, for example, although immunotherapy is a first-line option, immune exhaustion and “cold” subtypes persist, remaining insensitive to immune checkpoint blockade but potentially responsive to targeted agents [80].

Tumor heterogeneity, both inter- and intra-tumoral, significantly impacts immunotherapy outcomes. Even among initial responders, more than 60% of NSCLC patients and 30% of those with metastatic melanoma eventually develop resistant lesions, leading to recurrence [81, 82]. Mechanistically, resistance, whether primary or secondary, ultimately converges on T cell dysfunction and the accumulation of immunosuppressive cells, with immune checkpoint inhibitors failing to trigger effective immune responses. Current research therefore focuses on restoring effector cell function and overcoming immune evasion, including the development of molecular classification systems based on biomarkers to guide patient stratification and precision treatment, thereby mitigating resistance development [83]. Additionally, breaking away from traditional pathological classification concepts and establishing tumor molecular classification characteristics based on molecular markers will aid in patient stratification, thereby establishing more targeted precision treatment models, which will help mitigate the development of immunotherapy resistance.

ADCs combine targeted delivery with potent cytotoxic payloads, offering high efficacy with reduced systemic toxicity. However, resistance to ADCs has emerged as a substantial barrier to their clinical benefit. Target antigen–dependent resistance, characterized by reduced antigen expression, is common. For example, high HER2 expression correlates with better responses to breast cancer ADCs [84], whereas reduced expression or heterogeneity adversely affects outcomes [85]. Defects in endocytosis and lysosomal processing also contribute. ADCs require internalization and lysosomal degradation to release their cytotoxic payload. Impaired expression of endophilin A2 (Endo II) reduces HER2 internalization in breast cancer, diminishing response to T-DM1[86]. Abnormal caveolin-1 (CAV1) mediated endocytosis has been observed in resistant cell lines, lowering uptake efficiency [87]. Non-cleavable ADCs transported into cells are degraded in the acidic environment of lysosomes and by active lysosomal enzymes. However, in T-DM1-resistant tumor cell lines, not only were lysosomal alkalization and impaired lysosomal protease activity observed, but also abnormal V-ATPase (a proton pump regulating lysosomal acidification) activity was detected [88, 89].

Resistance can also arise from modifications in the payload target or from drug efflux. For instance, in non-Hodgkin lymphoma resistant to microtubule inhibitor monomethyl auristatin E (MMAE)-containing ADCs such as pinatuzumab vedotin and polatuzumab vedotin, multidrug resistance 1 (MDR1) upregulation mediates enhanced drug efflux [90]. Resistance to sacituzumab govitecan (SG) has been associated with topoisomerase-1 (TOP1) mutations (E418K and frameshift) and a novel tumor-associated calcium signal transducer 2 (TACSTD2) and its encoded protein trophoblast cell surface antigen-2 (TROP2) T256R missense mutation, both of which impair drug activity [91]. Overall, ADC resistance is a multifactorial process involving alterations at every stage of drug targeting, internalization, processing, and payload action, necessitating comprehensive mechanistic understanding for the development of next-generation ADC strategies.

## Molecular mechanisms underpinning drug resistance

Drug resistance in cancer is a dynamic, multifactorial adaptive process driven by complex interactions among host genetic background, intrinsic tumor cell plasticity, immune system dynamics, and the tumor microenvironment (TME) [92, 93]. As treatment paradigms have evolved from conventional chemotherapy to targeted therapy, immunotherapy, and combination regimens, the spectrum of resistance mechanisms has become increasingly diverse and complex. Across cancer types, tumor genetic and phenotypic heterogeneity, clonal evolution, and therapeutic pressure collectively shape the temporal and spatial dynamics of resistance, posing persistent clinical challenges [94–97].

**Fig. 2:**
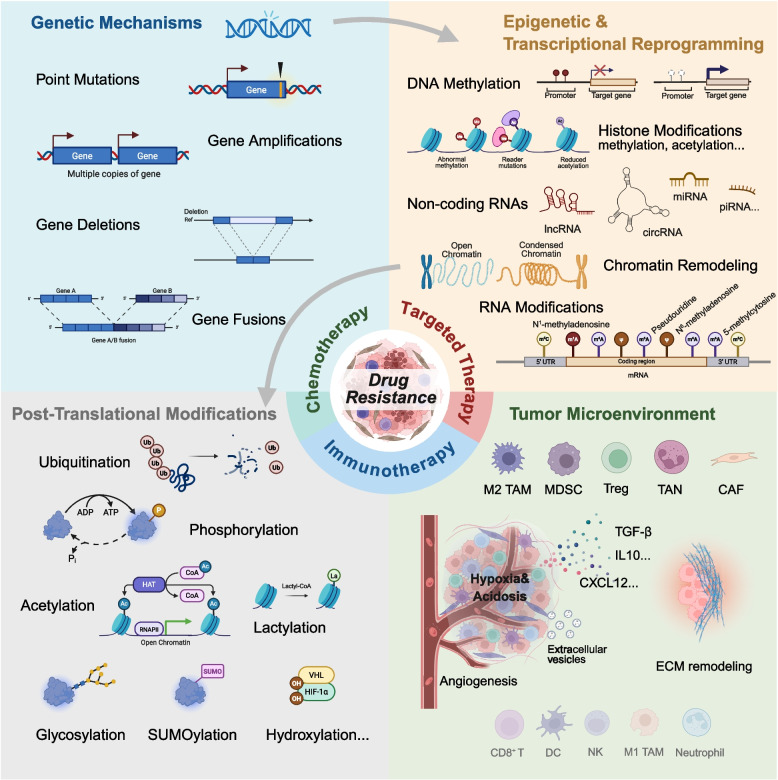
Molecular mechanisms underpinning drug resistance. Multiple mechanisms collectively mediate tumor resistance, including genetic mechanisms, epigenetic mechanisms, post-translational modifications, and the tumor microenvironment. TAM: Tumor-associated macrophages; MDSC: Myeloid-derived suppressor cells; Treg: Regulatory T cells; TAN: Tumor-associated neutrophils; CAF: Cancer-associated fibroblasts

This section provides a systematic overview of the core molecular mechanisms underlying drug resistance, encompassing genetic alterations, epigenetic and transcriptional reprogramming, proteomic and post-translational adaptations, and TME-mediated resistance (Fig. 2). By summarizing classical mechanisms alongside recent research advances, we aim to deepen the understanding of resistance diversity across cancer types and therapeutic modalities, thereby informing prediction and intervention strategies.

### Genetic mechanisms

Genetic alterations represent some of the earliest and most fundamental drivers of drug resistance. By modifying drug target structures, activating alternative signaling pathways, or enhancing cellular survival and repair capacity, these alterations confer selective advantages to tumor cells under therapeutic pressure (Fig. 2). Common genetic changes include acquired driver mutations, gene amplifications, fusions, and deletions, all of which have been implicated in resistance across multiple tumor types and treatment modalities [98–100].

In chemotherapy, genetic alterations can impair drug efficacy through various mechanisms [101]. TP53 mutations are particularly prevalent, reducing apoptosis in response to DNA damage and conferring broad resistance to chemotherapeutic agents [102, 103]. Upregulation of MDR1 leads to P-glycoprotein–mediated drug efflux, a well-established multidrug resistance mechanism [104, 105]. Resistance may also stem from mutations in direct drug targets. For example, TOP1 mutations (p.L617I, p.R621H, p.E710G) reduce the formation of TOP1–DNA cleavage complexes in response to camptothecin derivatives, leading to resistance to topoisomerase I inhibitors [106, 107]. β-tubulin gene mutations identified in paclitaxel-resistant cell lines reduce drug binding affinity to microtubules, lowering sensitivity [108, 109].

In targeted therapy, resistance often arises from mutations within the drug target or activation of bypass and downstream signaling pathways [110]. Steric hindrance mutations introduce bulkier amino acids at the drug-binding interface, obstructing inhibitor access. For example, the BCR–ABL1 T315I gatekeeper mutation blocks imatinib binding [111, 112]. Altered affinity mutations, such as EGFR T790M mutation increases ATP binding, reducing first- and second-generation EGFR inhibitor efficacy [113, 114]. Conformational change mutations disrupt the inactive conformation of kinases or stabilize their active state, sustaining kinase activation. For example, ALK mutations F1174L and L1196M stabilize the active conformation of ALK, reducing sensitivity to first-generation ALK inhibitors like crizotinib [115, 116]. Bypass signaling activation, such as PIK3CA mutations or PTEN loss activating PI3K/AKT, KRAS/NRAS/BRAF mutations reactivating ras proto-oncogene (RAS)/mitogen-activated protein kinases (MAPK), or janus kinase 2 (JAK2)/signal transducer and activator of transcription 3 (STAT3) activation, can restore proliferation despite target inhibition [117–122].

Immunotherapy resistance is frequently driven by genetic defects in antigen presentation and interferon-gamma (IFN-γ) signaling [110]. The former disrupts the stability of the MHC-I complex, leading to loss of MHC-I expression on tumor cells and preventing T cell-mediated recognition and killing, with B2M mutations or deletions being a key example [123, 124], while JAK1/JAK2 loss-of-function mutations impair IFN-γ responsiveness, reducing PD-L1 and MHC-I induction [125–127]. Additional alterations include ZNF689 deficiency, which promotes genomic instability and impairs antigen presentation [128], and recombination activating gene 21 (RAD21) amplification, which cooperates with yes-associated protein 1 (YAP)/TEA domain family member 4 (TEAD4) to suppress interferon signaling genes such as STAT1 and interferon regulatory factor 9 (IRF9) [129].

### Epigenetic and transcriptional reprogramming

Epigenetic regulation and transcriptional reprogramming are central to the development and modulation of drug resistance in cancer [130, 131]. Epigenetic regulation refers to heritable changes in gene expression that occur without alterations in the DNA sequence, mediated by mechanisms such as DNA methylation, histone modifications, chromatin remodeling, and non-coding RNAs (Fig. 2). These processes shape chromatin states and determine transcriptional potential [132]. Transcriptional reprogramming describes the dynamic adjustment of transcription factor activity, chromatin accessibility, and regulatory networks in response to external pressures, enabling tumor cells to establish gene expression programs that support adaptation and survival [133, 134]. Epigenetic alterations provide the permissive chromatin landscape that facilitates such transcriptional plasticity under stress [135, 136]. Dysregulation of these processes can silence tumor suppressor genes, remodel signaling pathways, alter the immune microenvironment, and drive metabolic adaptation, ultimately undermining therapeutic efficacy [137–139]. Importantly, epigenetic interventions, such as DNA methyltransferase and histone deacetylase inhibitors, have shown promise in partially reversing resistance [140, 141].

DNA methylation is a classical epigenetic mechanism involving the addition of a methyl group to cytosine residues, typically forming 5-methylcytosine, which alters chromatin conformation and transcriptional activity [142]. The mechanisms by which DNA methylation contributes to drug resistance vary across different therapeutic modalities [143]. In chemotherapy, hypermethylation-mediated silencing of tumor suppressor genes and regulation of DNA damage repair pathways are common. For instance, MutL homolog 1 (MLH1) promoter hypermethylation impairs mismatch repair, reducing sensitivity to platinum-induced DNA damage and promoting chemoresistance [144]. In targeted therapy, DNA methylation can modulate oncogenic signaling, alter target expression, or affect drug transport and metabolism. In clear cell renal cell carcinoma, QPCT promoter hypomethylation enhances nuclear factor kappa-B (NF-κB)-mediated transcription, stabilizes HRAS, and activates the extracellular regulated protein kinases (ERK) pathway, promoting sunitinib resistance [145]. In immunotherapy, DNA methylation can suppress antigen presentation, inhibit T cell–attracting chemokines, and induce immune evasion. For example, in hepatocellular carcinoma, Riplet promoter hypermethylation increases fatty acid synthase stability, enhances palmitic acid–dependent STAT3 activation, and induces CD8⁺ T cell exhaustion, reducing PD-1 blockade efficacy [146].

Histone modifications regulate gene expression by altering chromatin accessibility via covalent modification of histone tails [147]. Key modifications include acetylation, methylation, phosphorylation, and ubiquitination [148]. For example, trimethylation of histone H3 lysine 9 (H3K9me3) catalyzed by SUV39H1 promotes heterochromatin at the androgen receptor variant 7 (AR-V7) promoter in prostate cancer, sustaining AR-V7 expression and conferring resistance to enzalutamide [149]. Recently identified modifications such as lactylation, succinylation, citrullination, and crotonylation also affect transcription and resistance [150–152]. In colorectal cancer, hypoxia-induced lactate accumulation increases H3K18 lactylation (H3K18la), upregulating RUBCNL and activating autophagy, thereby mediating bevacizumab resistance [153]. In glioblastoma, lysine catabolism increases crotonyl-CoA and histone H4 crotonylation, suppressing interferon signaling and enabling immunotherapy resistance [154]. Furthermore, alterations in histone modification pathways also represent critical genetic events in tumor progression. For example, mutations in lysine demethylase 6A (KDM6A), exert gender-dual effects in urothelial carcinoma: enhancing immunotherapy response in males but promoting angiogenesis and poor outcomes in females, revealing mechanisms underlying gender differences in tumor immunotherapy response [155].Chromatin remodeling, the ATP-dependent repositioning of nucleosomes, regulates transcription factor access and gene expression [156, 157]. In EGFR-mutant NSCLC, SWI/SNF complexes sustain accessibility at oxidative stress–responsive loci, supporting NRF2 pathway activation and osimertinib resistance [158]. AP-1 transcription factors, particularly FOS-like antigen 1 (FOSL1) and JUN, cooperate with chromatin remodeling to drive resistance-specific transcriptional programs [159]. The presence of mutations in chromatin remodeling genes like ARID1A, coupled with high CD8 + T cell infiltration, is linked to improved prognosis in urothelial carcinoma patients receiving adjuvant chemotherapy and immunotherapy [160]. These findings underscore how chromatin remodeling facilitates resistance not merely through individual gene regulation but by orchestrating global, adaptable transcriptional networks [161].

Non-coding RNAs (ncRNAs), including microRNA (miRNA), long non-coding RNAs (lncRNA), and circular RNA (circRNA), modulate gene expression by influencing chromatin structure, mRNA stability, translation, and signaling pathways [162, 163]. In the context of cancer therapy resistance, ncRNAs contribute to adaptive survival under treatment stress by targeting critical signaling pathways, regulating drug transport and metabolism, and modulating apoptosis and immune evasion, thereby constituting a key epigenetic mechanism across multiple treatment modalities [162, 164, 165]. In breast cancer, miR-1275 downregulation derepresses MDK, activates PI3K/AKT, and increases stemness, promoting epirubicin resistance [166]. In clear cell renal cell carcinoma, lncARSR functions as a competing endogenous RNA (ceRNA) for miR-34 and miR-449, upregulating AXL and c-MET and activating downstream AKT and STAT3 signaling to drive sunitinib resistance [167]. In colorectal cancer, circPHLPP2 enhances interleukin-36 gamma (IL36γ) transcription via interleukin enhancer binding factor 3 (ILF3), suppressing natural killer (NK) cell infiltration and effector function, promoting anti-PD-1 resistance [168]. Collectively, these examples highlight the diverse roles of ncRNAs in driving therapy resistance. Beyond these, additional classes of ncRNAs, such as PIWI-interacting RNA (piRNA) and tRNA-derived fragments (tRFs), have also been implicated in drug resistance, pointing to an increasingly complex and still-evolving regulatory network [169–171].

RNA modifications represent a crucial layer of post-transcriptional gene regulation, and a wide array of chemical modifications have been identified across various RNA species [172]. Among the most well-characterized are N6-methyladenosine (m6A), N1-methyladenosine (m1A), 5-methylcytosine (m5C), pseudouridine (Ψ), and adenosine-to-inosine (A-to-I) editing [173]. These modifications affect splicing, stability, localization, translation, and degradation of mRNA, tRNA, rRNA, and lncRNA, thereby participating in fundamental cellular processes [174]. In the context of tumor progression, dysregulation of RNA modification regulators, including writers, erasers, and readers, has been widely implicated [175]. Through modulating oncogenic signaling pathways, enhancing stemness, promoting immune evasion, or enabling metabolic reprogramming, RNA modifications play central roles in maintaining tumor phenotypes and facilitating therapeutic resistance [176]. Among various RNA modifications, m6A is frequently highlighted due to its well-defined regulatory roles [177, 178]. Methyltransferase like 3 (METTL3), an m6A “writer,” is upregulated in lenvatinib-resistant hepatocellular carcinoma, enhancing EGFR mRNA translation and activating ERK signaling [179]. METTL3 also promotes PD-L1 mRNA stability via insulin-like growth factor 2 mRNA binding protein 3 (IGF2BP3), thereby impairing T cell-mediated cytotoxicity and reducing anti–PD-L1 therapy efficacy in breast cancer [180]. In parallel, m6A “erasers” also contribute to drug resistance. The m6A demethylase AlkB homolog 5 (ALKBH5) is upregulated in cisplatin-resistant ovarian cancer, where it forms a positive feedback loop with the transcription factor homeobox A10 (HOXA10), and promotes resistance by demethylating JAK2 mRNA, preventing its degradation by YTHDF2 and activating the JAK2/STAT3 signaling pathway [181]. Other modifications such as m1A and m7G, mediated by tRNA methyltransferase 6 (TRMT6)/TRMT61A and METTL1/WD repeat domain 4 (WDR4), have also been increasingly associated with therapy resistance, further highlighting the multifaceted roles of RNA modifications in shaping drug response [182–185].

During therapy, drug-induced selective pressure can trigger transcriptional reprogramming that remodels gene networks to sustain resistance [186]. Transcription factors such as cellular myelocytomatosis oncogene (c-MYC), forkhead box protein M1 (FOXM1), and hypoxia-inducible factor 1 alpha (HIF-1α) promote stemness, epithelial–mesenchymal transition (EMT), and immune evasion [187–189]. For instance, in chemotherapy-resistant breast cancer, MYC cooperates with myeloid cell leukemia sequence 1 (MCL1) to enhance oxidative phosphorylation and maintain cancer stem cell properties [190]. Enhancers, especially super-enhancers (SEs), can increase chromatin accessibility and recruit transcriptional coactivators, thereby amplifying the transcription of resistance-related genes and facilitating tumor cell adaptation under therapeutic pressure [191, 192]. In poly ADP-ribose polymerase (PARP) inhibitor (PARPi)–resistant ovarian cancer, lactate accumulation induced by enhanced glycolysis promotes H4K12la histone modification at the RAD23A promoter and its associated super-enhancer (Nira-SE), which facilitates MYC recruitment and drives RAD23A overexpression, ultimately enhancing DNA repair capacity and conferring niraparib resistance [193].

### Post-translational adaptations and modifications

Post-translational modifications (PTMs) of proteins profoundly influence their stability, localization, and activity, thereby regulating essential cellular processes and contributing to drug resistance in cancer [194, 195] (Fig. 2).

The dynamic regulation of ubiquitination and de-ubiquitination is a central mechanism in resistance to chemotherapy, targeted therapy, and immunotherapy by precisely regulating key proteins in tumor cells [196]. Alterations in these processes can stabilize drug transporters such as MDR1 and breast cancer resistance protein (BCRP) or DNA damage repair (DDR) proteins, enhancing drug efflux, promoting DNA repair, and facilitating cell survival [197–199]. Regulation of target protein stability also influences the sensitivity of targeted therapy and immunotherapy. For instance, ubiquitin-specific peptidase 28 (USP28) enhances SRY-box 9 (SOX9) stability and DNA damage repair, mediating resistance to PARP inhibitors [200]. Inhibiting USP8 increases PD-L1 and MHC-I expression, thereby improving responsiveness to PD-1/PD-L1 blockade [201].

Phosphorylation, typically regulated by kinase activity, modulates downstream signaling cascades and contributes to resistance. In renal cell carcinoma, reciprocal activation between ERK2 and T-LAK cell–derived protein kinase (TOPK) promotes sorafenib resistance in a phosphorylation-dependent manner [202]. Phosphorylation can also interact with ubiquitination to regulate protein stability. For example, in prostate cancer, methionine 1-linked ubiquitination (M1-Ubi) of PTEN at K144 and K197 inhibits its phosphatase activity, accelerating tumor progression and reducing sensitivity to enzalutamide [203]. Additionally, NDR1 phosphorylates the E3 ligase F-box protein 11 (FBXO11), enhancing β-catenin ubiquitination, promoting its degradation, and inhibiting nuclear translocation, thereby suppressing metastasis [204].

Acetylation, especially lysine acetylation, is another key PTM in resistance. On one hand, histone acetyltransferases (HATs) such as CBP and p300 can upregulate resistance-related genes, promoting tumor progression and drug resistance [205]. On the other hand, histone deacetylases (HDACs) can induce chromatin remodeling and epigenetic changes that counteract therapy [206]. In renal cell carcinoma, HDAC1-mediated deacetylation of p53 promotes lipid accumulation and sorafenib resistance [207]. Targeting acetylation, particularly through HDAC inhibitors, is therefore a promising strategy.

Emerging PTMs such as lactylation and SUMOylation have gained attention. Lactylation, catalyzed by lactate, modifies lysine residues on histones and non-histone proteins, regulating resistance pathways [208]. For example, H3 lysine 18 lactylation (H3K18la) mediates cisplatin resistance in bladder cancer [209], while H3K9la accumulates in the LUC7L2 promoter, promoting its transcriptional expression and temozolomide resistance in glioblastoma [210]. Non-histones such as X-ray repair cross-complementing 1 (XRCC1) undergo lactylation at the lysine 247 site (K247), which enhances transport to the cell nucleus and strengthens DNA repair capacity, thereby mediating glioblastoma resistance to therapy [211]. SUMOylation, the covalent attachment of small ubiquitin-like modifiers (SUMO) to proteins, can modulate their function, localization, and stability [212]. Upregulation of the SUMO protein E2 enzyme, UBC9, correlates with increased resistance in breast and liver cancers, and targeting UBC9 can reverse resistance [213]. SUMO2/3 modification of Ku80 at K307 inhibits oxaliplatin-induced apoptosis in colorectal cancer [214].

Importantly, PTM pathways often interact rather than operate independently. For example, SUMOylation can crosstalk with phosphorylation, acetylation, and ubiquitination to regulate drug sensitivity [215]. Such interplay highlights the complexity of PTM networks in resistance and the necessity of integrative strategies when developing interventions.

### Tumor microenvironment-mediated resistance

TME plays a pivotal role in mediating drug resistance by providing physical barriers, biochemical protection, and a dynamic ecosystem that enables tumor cells to evade immune clearance, adapt to therapeutic pressure, and activate survival pathways [216] (Fig. 2).

Immune cells, as the primary components of the TME, comprise both anti-tumor and immunosuppressive populations. Tumor-associated macrophages (TAMs) can suppress T cell activity by secreting cytokines such as transforming growth factor-β‌ (TGF-β) and by releasing exosomes, as well as by upregulating PD-L1 expression, thereby mediating resistance to immune checkpoint inhibitors (ICIs) [217]. Other immunosuppressive cells, including myeloid-derived suppressor cells (MDSCs), tumor-associated neutrophils (TANs), regulatory T cells (Tregs), and tumor-associated dendritic cells (tDCs), also contribute to immune evasion and tumor progression [218]. Tumor-associated fibroblasts (CAFs) produce dense extracellular matrix (ECM) components such as collagen, fibronectin, and hyaluronic acid, while secreting pro-survival factors like hepatocyte growth factor (HGF) [219]. These actions not only create physical and biochemical barriers that hinder drug penetration but also directly promote tumor survival and suppress T cell and NK cell activity. Additionally, CAFs can directly suppress T cell and NK cell infiltration and activity, and by inducing Tregs and TAMs, they shape an inhibitory immune microenvironment [220].

The tumor vasculature also contributes to resistance. Newly formed microvessels are often in the TME tortuous and leaky, reducing drug delivery efficiency and fostering hypoxia [221]. HIFs induced under these conditions promote resistance to targeted therapies such as TKIs and suppress anti-tumor immunity by increasing the production of metabolic byproducts like lactate [222]. Hypoxia also drives nutrient competition between tumor and immune cells, particularly for glucose, thereby impairing T cell and NK cell cytotoxicity and pushing the TME toward an immunosuppressive state [223, 224]. Furthermore, hypoxia-mediated acidification lowers the efficacy of weakly basic chemotherapeutics like adriamycin, whereas pH modulation toward alkalinity can mitigate resistance [225]. Endothelial cells within the vasculature can sense oxidative stress and secrete cytokines such as fibroblast growth factor 2 (FGF2) and chemokine (C-X-C motif) ligand 12 (CXCL12), directly inducing resistance and promoting immune escape by upregulating PD-L1, recruiting MDSCs, and inhibiting T cell function [226–229].

The ECM plays multiple critical roles in tumorigenesis and progression by providing mechanical support, regulating the microenvironment, and serving as a source of signaling molecules [230]. ECM–integrin interactions, particularly involving β1-integrin, αvβ3, and αvβ5, promote cancer stem cell (CSC) phenotypes and activate survival pathways such as PI3K/AKT and focal adhesion kinase (FAK), conferring drug resistance [231–233]. ECM components like TGF-β and type I collagen enhance CSC marker expression, reinforcing stemness [234, 235], while hyaluronic acid (HA) supports CSC maintenance via Twist- and TGFβ–Snail-dependent signaling [236]. ECM stiffness also influences therapy response. Increased stiffness enhances YAP/TAZ activity, promoting resistance to targeted agents in breast cancer and melanoma [237, 238]. Stiffness-associated resistance can involve augmented DNA repair. For instance, MAP4K4/6/7-mediated ubiquitin phosphorylation is reduced in tumor cells adjacent to stiff ECM, enhancing double-strand break repair and decreasing drug sensitivity [239]. Moreover, dense ECM deposition forms a physical barrier that impedes immune cell infiltration, while collagen fibers can directly damage T cells and impair cytotoxic function [240–242]. ECM-mediated YAP/TAZ nuclear translocation can also upregulate PD-L1 expression in tumor cells, further suppressing immune responses [243].

## Emerging strategies to overcome or prevent resistance

Tumor drug resistance remains a formidable challenge in modern oncology, often likened to the “Sword of Damocles” hanging over current treatment paradigms. Building on an in-depth understanding of the underlying mechanisms, the next step is to develop strategies that can overcome resistance and restore anti-tumor efficacy. Contemporary research addresses this problem from molecular, cellular, and tumor ecosystem perspectives, with the ultimate goal of achieving truly precise cancer therapy.

### Targeting resistance mechanisms directly

**Fig. 3:**
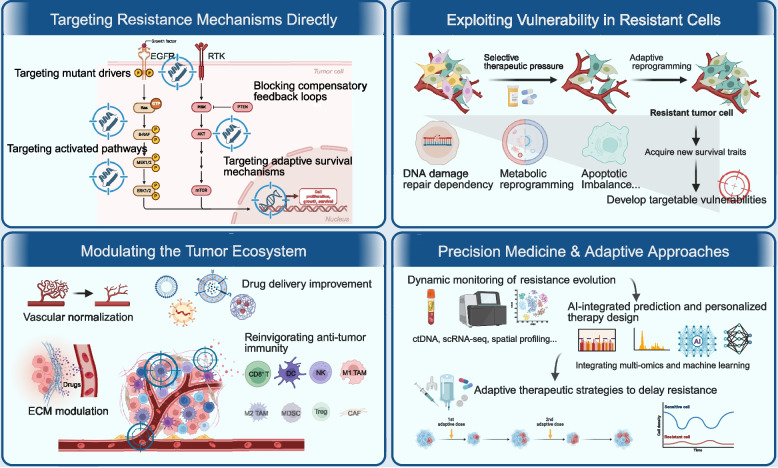
Emerging strategies to overcome or prevent resistance. The current main strategies for treating tumor resistance include directly targeting resistance mechanisms, targeting the vulnerabilities of resistant cells, repairing the tumor ecosystem, and using precision and adaptive treatment methods. TAM: Tumor-associated macrophages; MDSC: Myeloid-derived suppressor cells; Treg: Regulatory T cells; CAF: Cancer-associated fibroblasts

One of the most direct strategies involves designing specific inhibitors to target known drug-resistant mutations and their associated signaling pathways (Fig. 3 and Table 2). This approach relies on precise molecular characterization of resistance mechanisms. For example, fourth-generation EGFR-TKIs have been developed to address the T790M mutation, which confers resistance to first- and second-generation EGFR-TKIs, as well as the C797S mutation, which emerges after treatment with third-generation agents like osimertinib. These novel inhibitors not only retain activity against sensitive mutations such as L858R but also inhibit EGFR +/T790M and EGFR +/T790M/C797S variants, thereby overcoming resistance [244]. In chronic lymphocytic leukemia (CLL), covalent Bruton tyrosine kinase (BTK) inhibitors exert their activity by binding to cysteine residue 481 of the BTK protein [245]. When this residue mutates, covalent inhibitors lose binding ability and efficacy. In such cases, non-covalent BTK inhibitors such as pirtobrutinib can act independently of BTK mutation status, showing clinical activity in patients resistant to ibrutinib [246].

**Table 2:** Representative clinical studies currently conducted on strategies to overcome tumor drug resistance (2023–2025)

Agents	Purpose/Result	Status	Register ID
177Lu-PSMA-I&T/ Abiraterone/ Enzalutamide	Comparing the safety and efficacy of 177Lu-PSMA-I&T versus hormone therapy in patients with mCRPC	Active, not recruiting	NCT05204927
KN046/ Regorafenib/ Apatinib	Evaluating the efficacy and safety of KN046 in combination with regorafenib or apatinib for digestive system cancers resistant to PD-1/PD-L1 blockade	Recruiting	NCT06099821
B013/ Paclitaxel	Evaluating the efficacy and safety of B013 in patients with platinum-resistant recurrent ovarian cancer	Recruiting	NCT06434610
ILDR/ Anti-PD-1/PD-L1	Evaluating the efficacy and safety of combining intestinal low dose radiotherapy and PD-1/PD-L1 inhibitors for metastatic malignant solid tumors after acquired resistance to anti-PD1/PD-L1 treatment	Recruiting	NCT07071103
LTC004/ Toripalimab	Evaluating the efficacy and safety of LTC004 in combination with toripalimab in patients with advanced solid tumors resistant to first-line immunotherapy	Recruiting	NCT06490068
Cadonilimab/ Pemetrexed/ Anlotinib	Evaluating the efficacy and safety of cadonilimabin combination with pemetrexed and anlotinib for treatment of elderly patients with T790M-negative advanced non-squamous NSCLC following resistance to EGFR-TKI	Recruiting	NCT06277674
TVB-2640/ Enzalutamide	Evaluating the TVB-2640 administered in combination with enzalutamide in Men with mCRPC	Recruiting	NCT05743621
AB-1015	Evaluating the safety and efficacy of AB-1015 in patients with resistant/refractory epithelial ovarian cancer	Active, not recruiting	NCT05617755
Enhertu	Evaluating the efficacy and safety of Fam-Trastuzumab Deruxtecan-Nxki (T-DXd) as a subsequent line of therapy in HER2-positive metastatic castration-resistant prostate adenocarcinoma	Recruiting	NCT06610825
HRS-5041	Evaluating the safety, tolerability, pharmacokinetics and efficacy of 5041–103 in subjects with mCRPC	Recruiting	NCT06830850
Decitabine/ Carboplatin/ Paclitaxel/ Selinexor	Evaluating the efficacy and safety of combination of the hypomethylating agent decitabine and the nuclear export receptor XPO-1 inhibitor selinexor to reverse platinum resistance in relapsed/refractory epithelial ovarian cancer	Recruiting	NCT05983276
Inosine/Chemotherapy agents	Exploring the Efficacy of Inosine Reversing Chemo Resistance in Triple Negative Breast Cancer	Completed	NCT06355024
Adebrelimab/ Fuzuloparib	Evaluating the efficacy and safety of adebrelimab combined with fuzuloparib in the treatment of patients with recurrent platinum-resistant ovarian cancer	Recruiting	NCT05753826
Pembrolizumab/ Encorafenib/ Binimetinib	Evaluating the efficacy of transient addition BRAF and MEK Inhibitors to overcome primary resistance to immunotherapy in metastatic melanoma patients	Completed	NCT05304546
CUSP06	Evaluating the safety, tolerability, pharmacokinetics, and efficacy of CUSP06 in patients with platinum-refractory/resistant ovarian cancer and other advanced solid tumors	Recruiting	NCT06234423
SON-1010	Assessing the safety, tolerability, and PK/PD of SON-1010 in combination with atezolizumab administered to patients with advanced solid tumors and patients with platinum-resistant ovarian cancer	Recruiting	NCT05756907
Lenvatinib/ VIC1911	Testing the safety and efficacy of lenvatinib in combination with Aurora kinase A inhibitor VIC-1911 in participate with lenvatinib-unresponsive or lenvatinib-resistant hepatocellular carcinoma	Recruiting	NCT05718882
Nivolumab/ Ipilimumab	Evaluating the nivolumab and ipilimumab in patients with MSI/dMMR mCRC resistant to anti-PD1 monotherapy	Recruiting	NCT05310643
JANX007/ Darolutamide	Assessing the safety, tolerability, pharmacokinetic, pharmacodynamic, and the preliminary efficacy of JANX007 in adults with mCRPC	Recruiting	NCT05519449
HS-20093	Study to evaluate the efficacy, safety, tolerability and pharmacokinetic of HS-20093 as a monotherapy in subjects with mCRPC and other solid tumors	Recruiting	NCT06001255
LG002	Investigating the safety and efficacy of Neo-DCVac combined with ICIs in the treatment of advanced lung cancer resistant to ICIs	Recruiting	NCT06329908
Cabozantinib/ Nivolumab	Evaluating the combination of cabozantinib and nivolumab in subjects with advanced castration-resistant prostate cancer	Recruiting	NCT05502315
SX-682/ Enzalutamide	Studing the combination of SX-682 plus enzalutamide in men with mCRPC who have failed abiraterone	Recruiting	NCT06228053
BAY3546828	Evaluating the safety, tolerability, pharmacokinetics, and antitumor activity of actinium-225-macropa-pelgifatamab (BAY 3546828) in participants with advanced mCRPC	Recruiting	NCT06052306
SV-102	Evaluating the safety, tolerability, and efficacy of SYNC-T Therapy SV-102 and to identify the maximum tolerated dose (MTD) and/or selected dose for phase 2b study	Recruiting	NCT06533644
Gedatolisib/ Darolutamide	Evaluating the safety, preliminary efficacy, and pharmacokinetics of gedatolisib in combination with darolutamide in subjects with mCRPC	Recruiting	NCT06190899
TORL-1–23	Evaluating the safety and efficacy of TORL-1–23 in patients with advanced platinum-resistant epithelial ovarian cancer	Recruiting	NCT06690775
ZA-001	Evaluating the safety, whole-body distribution and radiation dosimetry of ZA-001 in mCRPC	Completed	NCT06359821
FPI-2265	Evaluating the safety and efficacy of FPI-2265 in patients with PSMA-positive mCRPC	Recruiting	NCT06402331
Apalutamide/ Carotuximab	Study of apalutamide with carotuximab in patients with mCRPC	Recruiting	NCT05534646
L-TIL/tislelizumab	Investigating the safety and efficacy of Liquid Tumor Infiltrating Lymphocytes (L-TIL) plus tislelizumab as second line therapy for PD-1 inhibitor resistant advanced NSCLC Patients	Recruiting	NCT05878028
OPB-101	Evaluating the safety and efficacy of OPB-101 in platinum-resistant ovarian cancer	Recruiting	NCT07030907
Carboplatin/ ^177^Lu-PSMA-617	Study of ^177^Lu-PSMA-617 plus carboplatin in mCRPC	Recruiting	NCT06303713
Docetaxel/ Carboplatin	Investigating platinum and taxane chemotherapy in mCRPC patients with alterations in DNA damage response genes	Recruiting	NCT06439225
JSB462/ AAA617	Study of JSB462 (Luxdegalutamide) in combination with lutetium (^177^Lu) vipivotide tetraxetan in adult male patients with PSMA-positive mCRPC	Recruiting	NCT07047118
^225^Ac-LNC1011/ ^68^ Ga-PSMA-11	Exploring the safety and efficacy of 225Ac-labeled LNC1011 for treating patients with PSMA-positive mCRPC	Recruiting	NCT07117760
HLD-0915	Assessing of the safety and efficacy of HLD-0915 as monotherapy in patients with mCRPC	Recruiting	NCT06800313
Dalpicilib/ Cetuximab	Evaluating the efficacy and safety of cetuximab combined with dalpicilib compared to cetuximab monotherapy in patients with HPV-negative, anti-PD-1-resistant recurrent or metastatic head and neck squamous cell carcinoma	Recruiting	NCT06935188
Bicalutamide/ Sunitinib	Evaluating the safety and efficacy of bicalutamide in combination with sunitinib in patients with receptor tyrosine kinase inhibitor resistant renal cell carcinoma	Recruiting	NCT06222593
Nimotuzumab/ Capecitabine	Exploring the efficacy and safety of a combination regimen of Anti-PD1 monoclonal antibody, nimotuzumab, and capecitabin in treating recurrent or metastatic nasopharyngeal carcinoma patients who have failed first-line platinum-based chemotherapy	Recruiting	NCT06259721
CD19/CD22 -CAR T cells	Investigating the safety, tolerability, and pharmacokinetic properties of human CD19-CD22 targeted T cells infusion for refractory/relapsed leukemia/lymphoma patients with or without central nervous system involvement	Recruiting	NCT06213636

*mCRPC *Metastatic Castration-Resistant Prostate Cancer, *ILDR* Low-dose radiotherapy to the intestine, *MSI* Microsatellite Instability, *dMMR *Deficient mismatch Repair, *NSCLC *Non-small cell lung cancer

Data sources: clinical registration website (https://clinicaltrials.gov)

The core of the strategy targeting resistance mechanisms directly lies in developing highly selective targeted drugs that address the direct molecular causes of resistance. Thus, by converting the mechanism of resistance from therapeutic endpoint into a starting point for drug design, this strategy marks a shift from passive adaptation to active elimination, enabling more precise targeting of tumor evolutionary escape routes.

### Exploiting vulnerabilities in resistant cells

Drug resistance is an outcome of evolutionary selection. While resistant cells survive by activating compensatory pathways or undergoing phenotypic changes, these adaptations may create new dependencies that can be exploited therapeutically. This concept aligns with synthetic lethality, targeting vulnerabilities such as DNA repair, metabolic reprogramming, or lineage plasticity, thereby transforming cancer cells' adaptive traits into fatal weaknesses (Fig. 3 and Table 2).

In platinum-resistant ovarian cancer, resistance acquisition is accompanied by increased ataxia-telangiectasia-mutated-and-Rad3-related kinase (ATR)-checkpoint kinase 1 (CHK1) activity. When PARP inhibitor–resistant cells are treated with a combination of PARPi and ATR inhibitors, sensitivity is restored through synergistic replication fork stalling, increased double-strand breaks, and apoptosis [247]. In KRAS ^G12C^-mutant lung and colorectal cancers, combining KRAS ^G12C^ inhibitors adagrasib with sevenless homologue 1 (SOS1) inhibitors (BI-3406) or src homology region 2-containing protein tyrosine phosphatase 2 (SHP2) inhibitors disrupts MRAS-driven receptor tyrosine kinase (RTK) feedback activation, enhancing anti-tumor efficacy and delaying resistance onset [248].

Metabolic targeting is another approach. In ibrutinib-resistant mantle cell lymphoma (MCL), glutamine synthase (GLS) overexpression correlates with glutamine dependency and metabolic levels. Inhibiting GLS with telaglenastat in combination with ibrutinib has shown synergistic activity [249]. In refractory melanoma, simultaneous inhibition of anti-apoptotic proteins MCL1 and BCLXL has achieved greater tumor suppression compared to single-agent inhibition [250].

Therefore, targeting the vulnerability of drug-resistant cells shifts cancer treatment from simply blocking signaling pathways to applying evolutionary principles, aiming to achieve long-term disease control by exploiting resistance-induced weaknesses.

### Modulating the tumor ecosystem

Drug resistance is rarely an isolated cellular event. It is shaped by the tumor’s broader ecological niche, including the physical barrier of the stroma, the immunosuppressive cellular infiltrate, and the metabolic environment. Targeting this ecosystem seeks to dismantle the protective niche of resistant clones (Fig. 3).

Degrading tumor stroma and normalizing vasculature can enhance drug delivery. HA is one of the main components of the extracellular matrix. By regulating the gel fluid pressure of the tumor interstitium, it can reduce the delivery of anticancer drugs [251]. In PDAC, pretreatment with pegylated recombinant human hyaluronidase (PEGPH20) reduces HA levels, lowers interstitial fluid pressure, and improves paclitaxel distribution, thereby enhancing efficacy [252]. Novel anti-angiogenic agents such as vanucizumab, which simultaneously inhibits VEGF-A and angiopoietin-2 (Ang-2), thereby significantly reducing abnormal tumor angiogenesis [253]. More importantly, this treatment approach provides a transient vascular normalization window that significantly enhances the efficacy of radiotherapy and chemotherapy [254].

Reprogramming the immune microenvironment is also promising. Macrophages, particularly M2 macrophages, have a unique inhibitory role in the tumor microenvironment, and the colony-stimulating factor 1 (CSF-1) receptor (CSF-1R) plays a crucial role in the recruitment and differentiation of monocytes into pro-tumor M2 macrophages and their survival [255]. On the other hand, inhibiting CSF-1R promotes the infiltration and activation of CD3 + CD8 + T cells in the tumor microenvironment, suppresses tumor immune escape, and enhances the antitumor activity of PD-1/PD-L1 inhibitors, thereby overcoming resistance to PD-1/PD-L1 axis blockade [256]. Enhancing antigen presentation processes has similar immunotherapy-sensitizing effects. Wu et al. found that higher levels of antigen-presenting mast cells (apMCs) in breast tissue are associated with enhanced anti-PD-1 therapy efficacy, while the allergy medication, cromolyn, can activate apMC-mediated T cell immunity and enhance tumor sensitivity to anti-PD-1 therapy, providing a new therapeutic strategy to overcome anti-PD-1 resistance [257].

Advanced drug delivery systems can overcome physical barriers. Nanocarriers engineered for receptor-mediated uptake, transporter exploitation, or lipophilic transcellular passage can cross the BBB in glioblastoma, enabling multifunctional delivery of agents that regulate metabolism, induce ferroptosis, or modulate immunity [258–260]. Light-responsive nanoplatforms targeting the TME can induce reactive oxygen species (ROS) production, enable multimodal imaging, and trigger immunogenic cell death in PDAC [261]. Co-delivery of gemcitabine and MnFe₂O₄ exhibit dual effects of chemotherapy and induction of ferroptosis, with this synergistic effect significantly enhancing treatment sensitivity [262]. These approaches all open new avenues for improving PDAC drug resistance and enhancing treatment sensitivity.

### Precision medicine and adaptive therapeutic approaches

The key to achieving precise treatment for tumor drug resistance lies in the early and dynamic identification of the evolution of drug resistance (Fig. 3). Therefore, establishing an early drug resistance warning model is an important means of achieving drug resistance monitoring. Jones et al. detected mutations in the mismatch repair (MMR) genes MSH2 and MSH6 in the plasma of glioma patients [263]. These mutations are not only the most common circulating genetic alterations following temozolomide treatment but are also detected earlier than recurrence, thereby achieving the goal of early resistance warning through circulating tumor DNA (ctDNA). Similarly, in colorectal cancer, ctDNA has been used for early identification of tumor resistance, monitoring of treatment response, and recurrence warning [264]. Some of the latest technologies are also being used for resistance monitoring. For example, single-cell transcriptomics and spatial transcriptomics technologies can not only identify resistant tumor cell subpopulations but also map tumor evolution at single-cell resolution [265]. This not only deepens our understanding of resistance mechanisms but also provides a more efficient way to discover new intervention targets [209, 266–268].

Building on these, large models established by deeply integrating multi-omics data such as epigenomics, genomics, transcriptomics, proteomics, metabolomics, and pharmacogenomics using artificial intelligence (AI) technology can not only further explore the underlying logic of tumor resistance but also enable early prediction of tumor resistance [269–271]. More importantly, by classifying and stratifying tumors with different gene expression characteristics, it can strongly support clinical decision-making and significantly enhance the precision of tumor treatment [272].

Additionally, some adaptive treatment modalities beyond traditional standard therapies have made important contributions to improving tumor treatment sensitivity. For example, in prostate cancer with PTEN deletion, Qi et al. found that intermittent administration of PI3K inhibitors, compared to daily dosing, more effectively activates CD8 + T cell-dependent antitumor immune responses, promoting sustained responses to anti-PD-1 therapy [273]. Based on the dynamic changes in the androgen receptor (AR) gene and AR-V7 in circulating tumor cells, researchers have established a method to predict sensitivity to chemotherapy and endocrine therapy in metastatic castration-resistant prostate cancer (mCRPC) [274]. Following docetaxel treatment for mCRPC, patients with liquid biopsy-negative AR and AR-V7 status benefited from combination therapy with enzalutamide, while those with positive status did not.

These measures effectively support the entire process of tumor drug resistance monitoring, prediction, and intervention, thereby promoting a paradigm shift in drug resistance control from empirical treatment to data-driven precision treatment.

### Innovative therapeutic approaches

#### Microbiome modulation

**Fig. 4:**
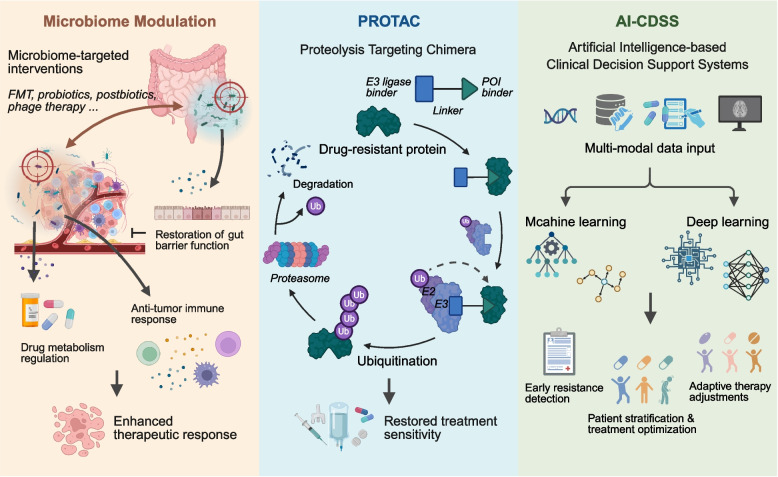
Clinical research progress regarding drug resistance. This schematic illustrates three innovative approaches targeting therapeutic resistance: microbiome-targeted therapies, PROTAC technology, AI-driven clinical decision support

The human microbiome, particularly the gut microbiota, has emerged as a critical regulator of therapeutic response in oncology (Fig. 4). Clinical evidence increasingly shows that microbial dysbiosis contributes to both primary and acquired resistance across multiple tumor types through three interconnected mechanisms: immunomodulation via metabolite-mediated T cell polarization, enzymatic drug metabolism altering chemotherapeutic bioavailability, and barrier disruption that promotes pro-tumorigenic inflammation.

Modulating the microbiome, through approaches such as fecal microbiota transplantation (FMT), precision probiotics, and phage-targeted decolonization, represents a paradigm-shifting strategy for resensitizing refractory malignancies. This section synthesizes recent advances in microbiome engineering for overcoming resistance, highlighting biomarkers and intervention frameworks with translational potential in precision oncology.

Antibiotic treatment has been shown to diminish the efficacy of PD-1 blockade in cancer patients, although the underlying mechanisms remain incompletely understood. Fidelle et al. demonstrated that antibiotic-induced downregulation of mucosal addressin cell adhesion molecule-1 (MAdCAM-1) in the ileum facilitates gut recolonization by Enterobacteriaceae [275]. This, in turn, reprograms gut-tropic α4β7⁺CD4⁺ regulatory T17 cells toward tumor dissemination. Moreover, low serum levels of soluble MAdCAM-1 were correlated with poor prognosis in independent cohorts of lung, kidney, and bladder cancer patients, suggesting that the MAdCAM-1–α4β7 axis could be a targetable gut immune checkpoint in cancer immunosurveillance. Joachim et al. developed an in vivo model in which C57BL/6J mice received oral supplementation with the bacterial metabolite deaminated tyrosine (DAT) under the regulation of type I interferon (IFN-I) [276]. DAT delayed tumor growth and enhanced the effects of anti-CTLA-4 or anti-PD-1 immune checkpoint inhibitors, effects that were dependent on host IFN-I signaling. Oral DAT supplementation altered gut microbiota composition, increasing bacterial taxa associated with favorable responses to immunotherapy.

Research into traditional Chinese medicine has also provided insights. Huang et al. investigated whether combining ginseng polysaccharides (GPs) with an anti-PD-1 monoclonal antibody could enhance therapeutic efficacy via modulation of the gut microbiota [277]. They found that the combination upregulated the microbial metabolite valeric acid, downregulated L-tryptophan, and reduced the kynurenine/tryptophan ratio, thereby decreasing regulatory T cell abundance and increasing effector T cell activity. This suggests that GPs in combination with anti-PD-1 may improve immunotherapy sensitivity in NSCLC.

Rodríguez-García et al. examined the role of urolithin A (UroA), a metabolite produced by the gut microbiota, in multiple myeloma (MM). Analysis of a retrospective cohort of 45 patients revealed that UroA synergized with bortezomib in vitro, indicating its potential as an adjunctive therapy to overcome resistance in MM [278]. Wu et al. investigated the inhibitory effect of flaxseed lignans (FL) on breast cancer biological behavior and evaluated the role of FL in enhancing the anticancer efficacy of PD-1/PD-L1 inhibitors (PDi) [279]. Following conversion of FL to enterolactone (ENL) by the gut microbiota, FL administration suppressed BC progression. ENL inhibited malignant BC behavior by downregulating CD38—a key gene associated with immunosuppression and resistance to PD-1/PD-L1 blockade. Consequently, FL enhances the anticancer efficacy of PDi through modulation of gut microbiota and host immunity.

Prior to the study by Xu et al., the potential role of the gut microbiota in modulating gastric cancer (GC) sensitivity to oxaliplatin remained uninvestigated [280]. Antibiotic treatment diminished the therapeutic efficacy of oxaliplatin in GC mouse models, an effect transferable to germ-free mice via fecal microbiota transplantation (FMT), suggesting gut microbiome involvement in oxaliplatin response. Further metabolomic data revealed that metabolically active Akkermansia muciniphila potentiated oxaliplatin efficacy. The researchers demonstrated that A. muciniphila-derived phenethylamine (PEA), acting as a glycolysis inhibitor, enhanced oxaliplatin response in gastric cancer cells by directly counteracting FUBP1 activity.

Current studies report inconsistent findings regarding the gut microbiome as a biomarker for ICIs response. Kim et al. identified TANB77, a previously uncultured and unique bacterial taxon, as exhibiting the highest enrichment in responders through a meta-analysis of ten independent ICIs-treated cohorts [281]. Murine models with higher gut TANB77 abundance showed improved responses to anti-PD-1 therapy. Furthermore, mice administered intraperitoneal injections of TANB77-derived proteins demonstrated enhanced anti-PD-1 responses, providing in vivo evidence for the therapeutic benefit of pirin-like proteins. These findings suggest that pirins from the TANB77 clade may potentiate ICIs responses across diverse cancer patient populations.

#### PROTACs in early-phase trials

PROTAC (Proteolysis-Targeting Chimera), a new biomedical tech, is used for selective and targeted protein degradation. It works by using the cell's natural ubiquitin–proteasome pathway. The PROTAC molecule has three parts: a targeting ligand that identifies and binds to the target protein; a linker that connects the parts; and an E3 ubiquitin ligase ligand that recruits the E3 ubiquitin ligase. When the PROTAC molecule binds to the target protein, the E3 ubiquitin ligase is brought close, leading to the target protein being marked by ubiquitination and then degraded by the proteasome (Fig. 4). This tech has advantages like strong targeting, high efficiency, and the ability to degrade traditionally undruggable targets. It shows great potential in treating tumors, neurodegenerative diseases, and other conditions, as well as in basic biological research.

Receptor-interacting serine/threonine-protein kinase 1 (RIPK1) functions as a critical stress sentinel coordinating cell survival, inflammation, and immunogenic cell death (ICD). While RIPK1's catalytic activity is essential for triggering cell death, its non-catalytic scaffolding function mediates potent pro-survival signaling. Mannion et al. synthesized a PROTAC that specifically degrades both human and murine RIPK1 [282]. PROTAC-mediated RIPK1 depletion disrupted TNFR1 and Toll-like receptor 3/4 (TLR3/4) signaling hubs, potentiating NF-κB, MAPK, and IFN signaling outputs. This potentiated ICD, enhanced antitumor immunity, and elicited sustained therapeutic responses. Consequently, targeting RIPK1 via PROTAC technology emerges as a promising strategy to overcome resistance to radiotherapy or immunotherapy and augment anticancer therapies.

Targeted protein degradation (TPD) regulates protein levels by redirecting E3 ligases via small molecules to ubiquitinate novel substrates, marking them for proteasomal destruction. TPD has recently emerged as a pivotal strategy in drug discovery. Schröder et al. engineered a potent DCAF1-BRD9 PROTAC, providing an alternative approach to address intrinsic resistance toward VHL-based degraders [283]. This highlights the potential of DCAF1-PROTACs as a promising strategy to overcome ligase-mediated resistance in clinical settings.

Pharmacological studies have led to the discovery of vepdegestrant (ARV-471), a selective, orally available and potent estrogen receptor (ER) degrader, which is a PROTAC—based small molecule. Gough et al. used biochemical and intracellular target—binding experiments to clarify how it works [284]. They also used ER + pre—clinical breast cancer models, including those with wild—type (WT) and mutated ESR1, to confirm that ER degradation can hinder tumor growth, suggesting vepdegestrant might be a more effective foundation for estrogen therapy for ER +/HER2—breast cancer patients. RIPK has a scaffolding function, causing resistance to ICIs and emerging as a promising target to enhance cancer immunotherapy. To address the challenge of the vague binding pocket in RIPK1's intermediate domain, Yu et al. used PROTAC technology to develop the RIPK1 degrader LD4172 [285]. As reported in the study, the RIPK1 degrader LD4172 can serve both as a chemical probe to investigate the scaffolding function of RIPK1 and as a potential therapeutic agent to increase tumor response to ICIs therapy.

Triple-negative breast cancer (TNBC) is the most aggressive breast cancer subtype, marked by high heterogeneity and invasiveness, with limited treatment options. Guo et al. used PROTAC technology to develop potential protein arginine methyltransferase 5 (PRMT5) degraders in vitro and in vivo. YZ-836P, a promising compound, showed cytotoxicity against TNBC cells after 48 h, reducing PRMT5 and kruppel-like factor 5 (KLF5) protein levels [286]. These findings position YZ-836P as a strong candidate for advancing TNBC treatment.

The chemotherapeutic modulating abilities of chemotherapy drugs are promising for addressing the low immunogenicity, immunosuppressive lactic microenvironment, and adaptive immune resistance in colorectal cancer. Zhao and colleagues developed self-assembling, self-delivering nanoPROTACs (DdLD NPs) containing doxorubicin (DOX) and dBET57, stabilized and facilitated by DSPE-PEG2000 [287]. DdLD NPs enhance the stability, cellular delivery, and tumor targeting of DOX and dBET57. They effectively kill colorectal cancer cells and induce ICD. These self-delivering nanoPROTACs may pave the way for chemo-enhanced tumor immunotherapy.

Osteosarcoma (OS) is the most common malignant bone tumor, and c-MET is a recognized therapeutic target. However, traditional c-MET inhibitors are limited by acquired drug resistance and side effects. c-MET—targeted PROTACs offer better antitumor effects by overcoming drug resistance, but their safety is still a concern due to the lack of tumor—targeting ability. Fu et al. generated four AS1411—SL1 chimeras and evaluated their therapeutic effects both in vitro and in vivo [288]. These AS1411—SL1 chimeras are likely to be promising c-MET degraders in osteosarcoma targeted therapy.

In NSCLC, EGFR is a key target as 60% of cases express it. However, resistance to EGFR inhibitors and limitations in strategies for both TKI—sensitive and mutant NSCLC patients remain concerns. Vartak et al. developed a nano—lipid EGFR and BRD4 degrader PROTAC (EPRO and BPRO) for lung cancer [289]. Using refined hydrolysis, the hydrophobic molecules were encapsulated in EGFR—targeted nano—lipid carriers (T—BEPRO). In mice with tumors, T—BEPRO administered intravenously achieved a remarkable tumor growth inhibition (TGI) rate of 77.6% and had lasting tumor suppression, surpassing drug—only treatment.

#### Artificial intelligence-powered clinical decision support

The formidable challenge of cancer drug resistance, characterized by extreme complexity, heterogeneity, and dynamic evolution across tumor types, remains a critical bottleneck in clinical oncology. Traditional approaches to predicting resistance patterns and optimizing subsequent therapeutic strategies often struggle to harness the vast, multidimensional datasets generated in modern oncology practice, encompassing clinical records, multi-omics profiles (genomic, transcriptomic, proteomic), medical imaging (radiomics/pathomics), and real-time monitoring data. This gap has catalyzed the rapid emergence of AI-based Clinical Decision Support Systems (CDSS) as a transformative frontier in resistance management. By leveraging sophisticated machine learning (ML) and deep learning (DL) techniques, including natural language processing and multimodal data fusion, AI-CDSS platforms are increasingly being deployed to decode intricate resistance mechanisms, predict individual patient risk trajectories with enhanced accuracy and timeliness, and generate evidence-based, personalized therapeutic recommendations (Fig. 4). This rapidly evolving field is transitioning from proof-of-concept studies towards tangible clinical integration, ultimately improving patient outcomes across diverse malignancies.

##### ML-based clinical decision support

ML algorithms learn patterns and relationships directly from historical data without explicit programming. Within CDSS, machine learning excels at predictive modeling by identifying patients at high risk for adverse events, disease recurrence, or, crucially, treatment resistance. It further enables risk stratification of patients based on predicted outcomes or treatment responses, and facilitates pattern recognition by detecting subtle associations within structured data.

The application of ML to cancer-specific pharmacogenomic datasets shows significant promise for identifying predictive response biomarkers, thereby enabling personalized treatment. Chia et al. introduced the precision oncology platform CAN-Scan, which utilizes ML technology to analyze next-generation pharmacogenetic datasets generated from cryopreserved biobank specimens of patient-derived primary cell lines (PDCs) [290]. CAN-Scan uncovered prognostic biomarkers and alternative therapeutic strategies, particularly for patients non-responsive to first-line chemotherapy. This approach demonstrates significant potential for improving biomarker discovery and guiding personalized therapy.

ICIs have revolutionized cancer treatment for multiple tumor types. However, a substantial proportion of patients treated with CPIs fail to derive benefit or experience only transient responses. Chen et al. developed a fibroblast senescence-associated transcriptomic signature (FSS), which is highly correlated with tumor-promoting signaling pathways and immune dysregulation that foster tumor progression [291]. Utilizing the FSS, a ML framework demonstrated remarkable accuracy in predicting ICI response and survival outcomes, achieving superior AUC values across validation, testing, and internal cohorts. Most notably, the FSS consistently outperformed established signatures in predicting robustness, encompassing diverse cancer subtypes. Fomin et al. employed a machine learning-based approach utilizing the de-identified, nationwide Flatiron Health-Foundation Medicine clinico-genomic database for NSCLC to identify genomic markers predictive of clinical response to ICIs therapy [292]. Building on this, they discovered multiple genomic markers and pathways that reveal the biological mechanisms influencing ICIs therapy response, potentially enhancing response rates to CPI therapy in NSCLC patients. Ricciuti et al. performed comprehensive tumor genomic sequencing, ML-based tumor-infiltrating lymphocyte (TIL) evaluation, multi-color immunofluorescence staining, and/or HLA-I immunohistochemistry (IHC) analysis on matched pre- and post-ICI treatment tumor biopsy samples from NSCLC patients treated at the Dana-Farber Cancer Institute who developed acquired ICI resistance [293]. This multi-modal analysis revealed the genomic and immunophenotypic heterogeneity underlying ICI resistance in NSCLC. To explore mechanisms of ICIs resistance, Sahni et al. developed the Immunotherapy Resistance cell–cell Interaction Scanner (IRIS), a machine learning model designed to identify cell type-specific tumor microenvironment ligand-receptor interactions associated with ICIs resistance [294]. They proposed a robust ICIs response biomarker, highlighting the critical role of downregulating ligand-receptor interactions related to chemotaxis in suppressing lymphocyte infiltration in resistant tumors.

Given that many patients fail to derive durable benefit, Hamidi et al. leveraged machine learning to analyze RNA sequencing data, targeted DNA panel results, immunohistochemistry, and digital pathology from 2,803 UC patients across four phase III randomized trials [295]. This approach identified four distinct transcriptomic subtypes predictive of response to the PD-L1 inhibitor atezolizumab.

Neoadjuvant chemotherapy and immunotherapy aim to eliminate residual tumor cells and reduce recurrence risk. However, drug resistance during neoadjuvant treatment poses a significant obstacle. Zhou et al. integrated advanced technologies including single-cell transcriptomics, whole-genome sequencing, RNA sequencing, proteomics, machine learning, and in vitro/vivo experiments [296]. By analyzing cross-cancer cohorts, they utilized single-cell sequencing to investigate the association between the efficacy of neoadjuvant chemotherapy/immunotherapy and RNA methylation. Multi-omics analysis coupled with machine learning algorithms identified genomic variations, transcriptional dysregulation, and prognostic correlations of RNA methylation regulators (RMRs), revealing distinct molecular subtypes that guide pan-cancer stratification for neoadjuvant therapy.

Chen et al. developed a machine learning-based systematic combinatorial design strategy to identify the most promising drug combinations for patients with relapsed/refractory (R/R) acute myeloid leukemia (AML) [297]. This predictive approach leverages single-cell transcriptomic data and monotherapy response profiles from primary patient samples to identify targeted combinations capable of selectively inhibiting therapy-resistant cancer cells within individual AML patient samples. Furthermore, in initial experiments using clinical trial samples, the method predicted the clinical efficacy of venetoclax-azacitidine combination therapy in AML patients. Collectively, this combined computational and experimental approach provides a rational pathway for identifying personalized combination therapies for R/R AML patients, targeting therapy-resistant leukemia cells and thereby enhancing its potential for clinical translation. Dysregulation of alternative splicing (AS) is increasingly recognized as a pivotal factor in the pathogenesis, disease progression, and therapeutic resistance of B-cell acute lymphoblastic leukemia (B-ALL). Zhuo et al. developed a prognostic model based on 18 AS events (18-AS), refined through the sophisticated integration of bioinformatic approaches and advanced machine learning algorithms [298]. These findings illuminate the role of AS events as novel prognostic biomarkers and therapeutic targets, advancing personalized therapeutic strategies in B-ALL management. Ianevski et al. described a machine learning-based approach, scTherapy, which leverages single-cell transcriptomic profiles to prioritize screening of multi-target therapeutic regimens for individual patients with hematological malignancies or solid tumors [299]. They established a broadly applicable strategy for identifying personalized treatment regimens capable of selectively and combinatorially inhibiting malignant cells while sparing non-cancerous cells, thereby enhancing the likelihood of clinical success.

Breast cancer patients may initially benefit from cytotoxic chemotherapy but subsequently develop treatment resistance and recurrence. Chemotherapy-resistant breast cancer stem cells (BCSCs) play a pivotal role in cancer relapse and metastasis. Leveraging a machine learning strategy, Sun et al. developed an mRNA-based BCSC signature to assess cancer stemness in primary breast cancer patient samples [300]. Through this BCSC signature, they revealed the critical role of polyamine synthesis in regulating chemotherapy-induced BCSC enrichment and proposed novel therapeutic avenues for breast cancer treatment.

Over 50% of patients with refractory high-grade serous carcinoma (HGSC) retain homologous recombination proficiency, rendering them resistant to platinum-based agents and PARP inhibitors. Tamura et al. conducted a comprehensive investigation of this tumor type by integrating machine learning analysis of large public datasets with a novel mouse oviduct-based genetically engineered HGSC organoid model [301]. This approach ultimately identified combination therapy involving conventional chemotherapy and mTOR inhibitors as a potential treatment strategy for HGSC, with p62 emerging as a significant biomarker.

DNA damage repair plays a pivotal role in HCC, driving tumorigenesis, progression, and treatment response [302]. Hong et al. proposed an innovative machine learning framework for the precise assessment of DDR, leveraging both scRNA-seq and bulk RNA sequencing data [303]. This model revealed dynamic interactions between DDR and NK cells and B cells within the primary HCC microenvironment. By shaping an immunosuppressive microenvironment that promotes tumor growth through metabolic reprogramming, the framework was ultimately utilized to predict the overall survival of HCC patients and their resistance to PD-1 therapy.

The efficacy of induction chemotherapy (IC) as a first-line treatment for advanced nasopharyngeal carcinoma (NPC) remains debated, and reliable biomarkers to predict its response are lacking. Tang et al. developed an AI-based radiomics approach to identify metabolic biomarkers through a discovery cohort-based machine learning methodology, with validation in a cohort simulating clinically challenging real-world scenarios [304]. The study revealed that dysregulation of plasma lipoproteins may drive IC resistance in NPC patients. The predictive model constructed from plasma metabolite profiles demonstrated strong predictive power and real-world generalizability. These findings hold significant implications for therapeutic strategy development and may offer potential targets to enhance IC efficacy.

Leveraging recent advances in machine learning, Zhao and colleagues investigated the impact of tumor mutations on the response to common therapeutic agents (conferring resistance) [305]. The resulting predictive model integrated numerous genetic alterations distributed across multiple molecular complexes. Applied to cisplatin-treated cervical cancer patients, the model revealed the regulatory role of the RTK-JAK-STAT complex in drug resistance, enabling a quantitative and interpretable assessment of drug response.

##### DL-based clinical decision support

DL is a powerful subset of machine learning that utilizes multi-layered artificial neural networks. The key strength of DL lies in its ability to automatically learn hierarchical representations and complex features from raw, high-dimensional, unstructured data. For instance, in medical imaging analysis (Radiomics/Pathomics), DL extracts intricate patterns beyond human perception from computed tomography (CT), magnetic resonance imaging (MRI), positron emission tomography (PET) scans, and histopathology slides, aiding in tumor characterization, treatment response assessment, and early detection of drug resistance. Similarly, for genomic/transcriptomic data interpretation, DL models complex interactions within high-dimensional omics data to uncover resistance mechanisms and biomarkers.

Relying solely on single-modality data often fails to comprehensively capture the complex heterogeneity among patients, including variations in resistance to anti-HER2 therapy and differences in the efficacy of combination treatment regimens, particularly in the management of HER2-positive gastric cancer [306]. Chen et al. collected multimodal data, encompassing radiological, pathological, and clinical information, from a cohort of 429 patients [307]. They further introduced a deep learning model, termed the Multimodal Mode, which integrates these diverse data streams to enable precise prediction of treatment response. This model achieved an AUC score of 0.821 for overall response prediction, with a remarkable AUC of 0.914 specifically for predicting response to combined immunotherapy. This study thus underscores the critical importance of multimodal data analysis in enhancing treatment evaluation and advancing personalized medicine for HER2-positive GC. The rapidly expanding pool of scRNA-seq data provides large-scale bulk gene expression databases for drug screening, enabling the identification of optimal clinical applications for anticancer drugs by investigating heterogeneity in drug responses across cancer cell subpopulations. Chen et al. proposed scDEAL, a deep transfer learning framework, for predicting cancer drug response at the single-cell level [308]. A key innovation of scDEAL is its integration of drug-related bulk RNA-seq data with scRNA-seq data, coupled with the transfer of models trained on bulk RNA-seq data to predict drug response in scRNA-seq data. scDEAL facilitates the investigation of cell reprogramming, drug selection, and drug repurposing to enhance therapeutic outcomes.

The identification of molecular features that mediate clinically aggressive phenotypes in prostate cancer remains a significant biological and clinical challenge [309, 310]. Elmarakeby and colleagues developed P-NET, a biologically informed deep learning model, to stratify prostate cancer patients based on treatment-resistant status and to evaluate the molecular drivers of treatment resistance through the model's full interpretability, thereby enabling therapeutic targeting [311]. They demonstrated that P-NET can utilize molecular data to predict cancer states, outperforming other modeling approaches. Their work substantiates that biologically informed, fully interpretable neural networks can enable preclinical discovery and clinical prediction in prostate cancer, with potential broad applicability across multiple cancer types. Gallagher et al. applied deep reinforcement learning (DRL) to guide adaptive drug dosing regimens [312]. They demonstrated that these regimens extended the time to disease progression by more than twofold compared to current adaptive protocols in mathematical models dynamically aligned with prostate cancer. This underscores DRL's capability to develop therapeutic strategies in novel or complex settings. Collectively, the DRL-generated personalized treatment regimens outperformed clinical standard-of-care regimens across all scenarios.

Cyclin-dependent kinase 4 and 6 inhibitors (CDK4/6i) have revolutionized the treatment landscape for breast cancer. However, objective responses are achieved in fewer than 50% of patients, and nearly all patients eventually develop resistance during therapy [313, 314]. Park et al. constructed an interpretable deep learning model, based on a reference atlas of multiprotein complexes in cancer, to predict response to the CDK4/6i drug palbociclib [315]. The predictions were applicable to both patients and PDX models, a capability unattainable with single-gene biomarkers. This study enabled the comprehensive assessment of how a tumor's genetic features modulate resistance to CDK4/6i. Furthermore, trastuzumab, a monoclonal antibody targeting HER2, is an effective therapy for metastatic breast cancer [316]. However, a subset of patients develop resistance to this treatment, making the monitoring of its therapeutic efficacy crucial [317]. Kim et al. described a deep learning-assisted method based on surface-enhanced Raman spectroscopy (SERS) immunoassay to monitor trastuzumab efficacy [318]. This method specifically targets HER2-overexpressing exosomes in murine urine. By employing SERS-deep learning analysis to monitor drug efficacy in urine exosomes from trastuzumab-treated mice, they confirmed that this monitoring system enables a proactive response to the problem of treatment resistance.

The EGFR genotype is critical for treatment decision-making in lung cancer; however, its testing results may be affected by tumor heterogeneity and biopsy procedures [319]. Importantly, not all patients harboring EGFR mutations achieve favorable outcomes following treatment with EGFR-TKIs, highlighting the necessity for stratification of EGFR mutant genotypes [320]. Wang et al. included 18,232 lung cancer patients from nine cohorts in China and the United States, all of whom underwent CT imaging and EGFR gene sequencing [272]. Based on this comprehensive dataset, they developed a fully artificial intelligence-based system (FAIS) that predicts both EGFR genotype and prognosis for EGFR-TKI therapy by analyzing whole-lung information derived from CT images.

Elucidating the molecular mechanisms underlying metabolic reprogramming to develop personalized risk prognosis assessment methods for hierarchically guided therapeutic strategies holds significant clinical importance for neuroblastoma (NB). Jin et al. employed a machine learning-based multi-step procedure to clarify the synergistic mechanisms by which metabolic reprogramming drives malignant progression in NB at both the single-cell and metabolic flux levels [321]. Subsequently, a metabolic reprogramming-related prognostic signature (MPS) based on MPS stratification, along with personalized treatment strategies, was developed and independently validated using preclinical models. This study provides profound insights into the molecular mechanisms of metabolic reprogramming-mediated malignant progression in NB. It also offers a novel perspective for developing targeted therapeutics based on innovative precision risk prediction methodologies, which are anticipated to contribute significantly to the advancement of NB treatment strategies.

Dysregulation of BCL2 family proteins plays a critical role in leukemogenesis and progression [322]. Consequently, pharmacological inhibition of these proteins is increasingly becoming a common therapeutic approach. However, its efficacy is compromised in clinical or preclinical studies due to the emergence of primary and acquired resistance [323]. Shah et al. developed a drug sensitivity prediction model based on a deep tabular learning algorithm to assess Venetoclax sensitivity in T-cell acute lymphoblastic leukemia (T-ALL) patient samples [324]. Several response biomarkers for ICIs show promise but have yet to achieve clinical-scale application. Johannet et al. developed a pipeline integrating deep learning on histological specimens with clinical data to predict ICI response in patients with advanced melanoma [325]. This multivariable classifier achieved an AUC of 0.800 on Aperio AT2 images and 0.805 on Leica SCN400 images. It accurately stratified patients into high-risk and low-risk groups, demonstrating its potential for integration into clinical practice.

Integrating multiple types of biological data is crucial for a comprehensive understanding of cancer biology; however, this task remains challenging due to data heterogeneity, complexity, and sparsity. Cai et al. proposed an unsupervised deep learning model, Multi-Omic Synthetic Augmentation model (MOSA), specifically designed to integrate and augment the Cancer Dependency Map (DepMap) [326]. MOSA successfully revealed multi-omic features critical for cell clustering and the identification of biomarkers associated with drug and genetic dependencies. The hypoxic microenvironment is often resistant to multiple therapeutic modalities, prompting the development of hypoxia-activated prodrugs (HAPs) to target these resistant regions [327]. The HAP evofosfamide (TH-302) has shown promise in preclinical and early clinical trials for sarcoma [328]. Jardim-Perassi et al. developed a DL model utilizing multiparametric MRI and registered pathology images to identify hypoxic regions and monitor the therapeutic response to TH-302 in rhabdomyosarcoma PDX models and syngeneic fibrosarcoma models. This study demonstrates that AI analysis of pre-treatment MRI images can predict hypoxia status and subsequent response to HAPs. This approach can be used to monitor treatment response and adapt therapeutic regimens to prevent the emergence of resistance. Zhang et al. leveraged a single-cell transcriptomic atlas encompassing diverse cancer and tissue types to reveal heterogeneous expression patterns within malignant cells, premalignant cells, and cancer-associated stromal and endothelial cells [329]. We propose a deep learning framework named Shennong for in vitro screening of anticancer drugs, designed to target each distinct cell population within the transcriptomic landscape. This robust and interpretable framework holds promise for accelerating the drug discovery process and enhancing the accuracy and efficiency of drug screening. The increasing availability of pharmacological data and the rapid advancement of deep learning methods have enabled the construction of models for predicting and screening drug combinations. To address the gap in virtual screening of drug combinations within large-scale databases, Ye et al. proposed the ScaffComb framework. Inspired by phenotype-directed drug design, ScaffComb integrates phenotypic information into molecular scaffolds [330]. This framework can be applied to screen drug libraries and identify high-efficacy drug combinations. Protein kinases (PKs) regulate diverse cellular functions and serve as targets for small-molecule kinase inhibitors (KIs) in cancer and other diseases. However, KI resistance has become a common clinical complication affecting multiple cancers, targeted kinases, and drugs. To address this challenge, Lin et al. leveraged multimodal features and deep hybrid learning to report an upgraded web server, Dr. Kinase, for predicting four drug resistance hotspot sites and assessing the impact of mutations on previously identified DR hotspots in PKs [331].

## Translational challenges and future perspectives

Translating mechanistic insights into clinically actionable strategies for overcoming therapeutic resistance faces multifaceted challenges across biological, technological, and clinical domains. Biologically, tumor heterogeneity and evolutionary dynamics generate parallel resistance mechanisms across spatially distinct niches, while the plasticity of the tumor–immune ecosystem enables continuous immune evasion [332–334]. Technologically, conventional biopsies often fail to capture real-time clonal adaptation, and preclinical models such as immortalized cell lines lack the microenvironmental complexity necessary for accurate target validation [335]. Clinically, the compartmentalization of resistance mechanisms by cancer type complicates the development of universal therapeutic strategies, and pharmacodynamic decoupling is common when targeting non-genetic adaptive states.

**Fig. 5:**
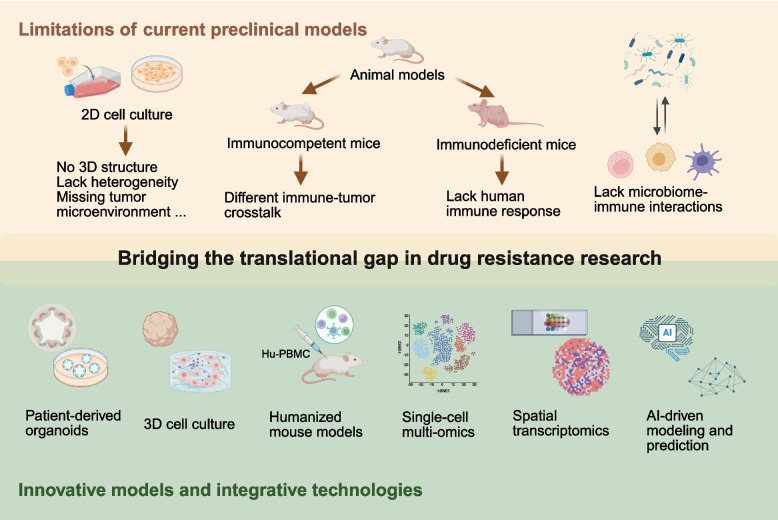
Bridging the translational gap in drug resistance research. Schematic summary of major limitations in current preclinical models and emerging approaches to enhance translational relevance. Conventional 2D cultures and animal models lack human immune-microbiome interactions and fail to predict clinical responses. Innovative models and technologies, including patient-derived organoids, humanized mice, single-cell and spatial omics, and AI-driven prediction, provide more physiologically relevant platforms for study

Emerging solutions include functional precision medicine platforms that integrate patient-derived organotypic cultures with single-cell multi-omics, AI-driven digital twins that simulate resistance evolution, and adaptive clinical trial designs incorporating biomarker-guided treatment arms. Achieving success will require redefining therapeutic goals from maximal tumor cell eradication to ecological containment of resistant clones, leveraging evolutionary principles to design “extinction therapies” (Fig. 5).

### Limitations of preclinical models

Conventional preclinical models have fundamental limitations in reproducing the dynamics of human therapeutic resistance, primarily due to their inability to capture the integrated tumor–immune–stromal ecosystem and the spatiotemporal heterogeneity of clinical tumors [336]. In vitro monolayer cultures fail to replicate ECM architecture, biomechanical forces, and metabolic gradients, contributing to the > 90% attrition rate of oncology drugs that demonstrate efficacy in vitro but fail in clinical trials [337, 338].

Murine models face parallel constraints. Syngeneic tumors in immunocompetent mice lack human-specific immune interactions shaped by human leukocyte antigen (HLA) diversity, whereas patient-derived xenografts (PDXs) in immunodeficient mice exclude adaptive immunity, which is essential for evaluating immunomodulatory agents [339]. Additionally, most models overlook the microbiome’s influence on drug metabolism and immune activation, as illustrated by germ-free conditions abolishing the microbiota-dependent efficacy of CTLA-4 blockade [340, 341].

Innovative approaches are addressing these deficits: patient-derived organotypic cocultures incorporating autologous immune cells and fibroblasts; computational digital twins trained on longitudinal multi-omics datasets to model resistance evolution; and humanized microbiome–immune models reconstituted with patient-derived microbiota [342]. These advances, aligned with frameworks such as the National Institutes of Health (NIH) Human Tumor Atlas Network’s spatial profiling standards, promise model systems with enhanced predictive value for resistance research (Fig. 5).

### Emerging frontiers in resistance research

High-resolution technologies are reshaping our understanding of resistance by revealing spatial, temporal, and molecular dynamics previously inaccessible. Single-cell multi-omics platforms, such as paired single-cell RNA (scRNA)-seq and single-cell assay for transposase-accessible chromatin (scATAC)-seq, can track transcriptional and epigenetic reprogramming within rare resistant subclones, showing how phenotypic plasticity enables transient drug tolerance without genetic changes [343]. Spatial transcriptomics platforms map resistance niches in situ, identifying immunosuppressive stromal barriers that shelter persister cells, as observed in PDAC where fibrotic zones harbor quiescent tumor cells with high survival gene expression [344].

Epi-transcriptomic profiling, including m6A-seq and pseudouridine mapping, has uncovered RNA modification–driven adaptive pathways [345]. For example, FTO-mediated m6A demethylation in leukemia stem cells increases BCL2 mRNA stability, conferring venetoclax resistance independent of genomic mutations [346]. These insights have revealed novel vulnerability categories: metabolic dependencies in hypoxic niches, context-specific synthetic lethalities triggered by RNA-modifying enzyme dysregulation, and neoepitope-independent T cell recognition sites exposed by altered RNA modifications (Fig. 5).

### Towards proactive management and preemptive strategies

Shifting from reactive to proactive resistance management represents a fundamental change in oncology. Traditional strategies often address resistance only after it becomes clinically evident, limiting treatment options. Future approaches must prioritize early detection and intervention before resistant clones become dominant.

Liquid biopsy platforms enable minimally invasive, real-time monitoring of ctDNA, circulating tumor cells (CTCs), and other biomarkers [347]. Early detection of resistance-associated mutations or clonal expansions creates an opportunity to intervene before clinical progression.

Machine learning–based risk prediction algorithms can integrate longitudinal liquid biopsy data, imaging, and clinical records to forecast resistance emergence, allowing for timely and personalized treatment adjustments.

Adaptive therapeutic cycling, alternating between agents or modalities based on molecular feedback, can suppress resistant populations by preventing any single clone from gaining a permanent advantage. This strategy aligns with evolutionary principles, aiming to control tumor growth by managing clonal dynamics rather than eradicating all cancer cells outright (Fig. 5).

## Conclusion

Research into tumor drug resistance faces three core challenges: at the biological level, tumor heterogeneity and clonal evolution lead to the dynamic accumulation of resistance mechanisms, while the physical barriers of the microenvironment limit drug delivery. At the technical level, existing models struggle to simulate the human ecosystem, and static biopsies cannot capture the real-time evolution of resistant clones. At the clinical level, resistance accounts for 90% of chemotherapy failures and over 50% of targeted/immunotherapy failures, while combination strategies often fail to sustain efficacy due to cross-resistance and increased toxicity. These challenges collectively hinder improvements in treatment outcomes.

Currently, strategies to address drug resistance issues mainly focus on directly targeting drug resistance mechanisms, microenvironment remodeling, and adaptive treatment. Although the concept of precise dynamic intervention based on ctDNA early warning of drug-resistant clones and AI multi-omics model prediction of evolutionary trajectories has been proposed, the above methods still face translational challenges. Directly targeting strategies are limited by tumor adaptive escape, microenvironment regulation exhibits spatiotemporal heterogeneity, and clinical models still inadequately simulate complex mechanisms such as microbiota-immune interactions.

Therefore, future research should focus on interdisciplinary innovation to overcome current bottlenecks, including: 1) Technological innovation, developing high-resolution dynamic monitoring technologies, such as single-cell spatial multi-omics analysis of drug-resistant niches, combining liquid biopsy to track epigenetic evolution, constructing humanized immune-microbiome chimeric models, and integrating patient-derived organoids with microbiota transplantation to simulate real microenvironments. 2) Targeted expansion, delving into non-genetic adaptive mechanisms, such as the regulation of immune memory remodeling by microbial metabolites and novel PTM interaction networks. 3) Clinical paradigm innovation, promote “evolution-guided therapy” through AI-driven adaptive clinical trials, intermittent dosing to activate anti-tumor immunity, and multifunctional nanocarriers to achieve tumor ecological regulation.

These measures will help establish a proactive drug resistance early warning, targeted elimination, and ecological control prevention and control system, ultimately driving a paradigm shift in cancer treatment from passive response to drug resistance to active ecological regulation, and achieving the long-term disease management goal of controllable drug resistance.

## Data Availability

Not applicable.

## References

[1] Bray F, Laversanne M, Sung H, Ferlay J, Siegel RL, Soerjomataram I, et al. Global cancer statistics 2022: GLOBOCAN estimates of incidence and mortality worldwide for 36 cancers in 185 countries. CA Cancer J Clin. 2024;74(3):229–63. 10.3322/caac.21834. 38572751 10.3322/caac.21834

[2] Bray F, Laversanne M, Weiderpass E, Soerjomataram I. The ever-increasing importance of cancer as a leading cause of premature death worldwide. Cancer. 2021;127(16):3029–30. 10.1002/cncr.33587. 34086348 10.1002/cncr.33587

[3] Matuszczak M, Kiljanczyk A, Salagierski M. Surgical Approach in Metastatic Renal Cell Carcinoma: A Literature Review. Cancers (Basel). 2023;15(6). 10.3390/cancers15061804. 10.3390/cancers15061804 PMC10046362 36980688

[4] Hickman L, Contreras C. Gallbladder Cancer: Diagnosis, Surgical Management, and Adjuvant Therapies. Surg Clin North Am. 2019;99(2):337–55. 10.1016/j.suc.2018.12.008. 30846038 10.1016/j.suc.2018.12.008

[5] Meyerhardt JA, Fuchs CS. Chemotherapy options for gastric cancer. Semin Radiat Oncol. 2002;12(2):176–86. 10.1053/srao.2002.30823. 11979419 10.1053/srao.2002.30823

[6] Smith CEP, Prasad V. Targeted cancer therapies. Am Fam Physician. 2021;103(3):155–63. 33507053

[7] Rui R, Zhou L, He S. Cancer immunotherapies: advances and bottlenecks. Front Immunol. 2023;14:1212476. 10.3389/fimmu.2023.1212476. 37691932 10.3389/fimmu.2023.1212476 PMC10484345

[8] Cai M, Song XL, Li XA, Chen M, Guo J, Yang DH, et al. Current therapy and drug resistance in metastatic castration-resistant prostate cancer. Drug Resist Updat. 2023;68:100962. 10.1016/j.drup.2023.100962. 37068396 10.1016/j.drup.2023.100962

[9] Gao X, Aguanno D, Board M, Callaghan R. Exploiting the metabolic energy demands of drug efflux pumps provides a strategy to overcome multidrug resistance in cancer. Biochim Biophys Acta Gen Subj. 2021;1865(8):129915. 10.1016/j.bbagen.2021.129915. 33965440 10.1016/j.bbagen.2021.129915

[10] Herzog SK, Fuqua SAW. ESR1 mutations and therapeutic resistance in metastatic breast cancer: progress and remaining challenges. Br J Cancer. 2022;126(2):174–86. 10.1038/s41416-021-01564-x. 34621045 10.1038/s41416-021-01564-x PMC8770568

[11] Keresztes D, Kerestely M, Szarka L, Kovacs BM, Schulc K, Veres DV, et al. Cancer drug resistance as learning of signaling networks. Biomed Pharmacother. 2025;183:117880. 10.1016/j.biopha.2025.117880. 39884030 10.1016/j.biopha.2025.117880

[12] An J, Peng C, Tang H, Liu X, Peng F. New Advances in the Research of Resistance to Neoadjuvant Chemotherapy in Breast Cancer. Int J Mol Sci. 2021;22(17). 10.3390/ijms22179644. 10.3390/ijms22179644 PMC8431789 34502549

[13] Labrie M, Brugge JS, Mills GB, Zervantonakis IK. Therapy resistance: opportunities created by adaptive responses to targeted therapies in cancer. Nat Rev Cancer. 2022;22(6):323–39. 10.1038/s41568-022-00454-5. 35264777 10.1038/s41568-022-00454-5 PMC9149051

[14] Vesely MD, Zhang T, Chen L. Resistance mechanisms to anti-PD cancer immunotherapy. Annu Rev Immunol. 2022;40:45–74. 10.1146/annurev-immunol-070621-030155. 35471840 10.1146/annurev-immunol-070621-030155

[15] Chen YF, Xu YY, Shao ZM, Yu KD. Resistance to antibody-drug conjugates in breast cancer: mechanisms and solutions. Cancer Commun. 2023;43(3):297–337. 10.1002/cac2.12387. 10.1002/cac2.12387 PMC10009672 36357174

[16] Kwon M, An M, Klempner SJ, Lee H, Kim KM, Sa JK, et al. Determinants of response and intrinsic resistance to PD-1 blockade in microsatellite instability-high gastric cancer. Cancer Discov. 2021;11(9):2168–85. 10.1158/2159-8290.CD-21-0219. 33846173 10.1158/2159-8290.CD-21-0219

[17] Saleh R, Elkord E. Acquired resistance to cancer immunotherapy: role of tumor-mediated immunosuppression. Semin Cancer Biol. 2020;65:13–27. 10.1016/j.semcancer.2019.07.017. 31362073 10.1016/j.semcancer.2019.07.017

[18] Mansoori B, Mohammadi A, Davudian S, Shirjang S, Baradaran B. The different mechanisms of cancer drug resistance: a brief review. Adv Pharm Bull. 2017;7(3):339–48. 10.15171/apb.2017.041. 29071215 10.15171/apb.2017.041 PMC5651054

[19] Wang X, Zhang H, Chen X. Drug resistance and combating drug resistance in cancer. Cancer Drug Resist. 2019;2(2):141–60. 10.20517/cdr.2019.10. 34322663 10.20517/cdr.2019.10 PMC8315569

[20] Seyedi S, Harris VK, Kapsetaki SE, Narayanan S, Saha D, Compton Z, et al. Resistance management for cancer: lessons from farmers. Cancer Res. 2024;84(22):3715–27. 10.1158/0008-5472.CAN-23-3374. 39356625 10.1158/0008-5472.CAN-23-3374 PMC11565176

[21] Abouzeid HA, Kassem L, Liu X, Abuelhana A. Paclitaxel resistance in breast cancer: current challenges and recent advanced therapeutic strategies. Cancer Treatment and Research Communications. 2025;43:100918. 10.1016/j.ctarc.2025.100918. 40215760 10.1016/j.ctarc.2025.100918

[22] Fahmy SA, Mahdy NK, Mohamed AH, Mokhtar FA, Youness RA. Hijacking 5-Fluorouracil Chemoresistance in Triple Negative Breast Cancer via microRNAs-Loaded Chitosan Nanoparticles. Int J Mol Sci. 2024;25(4). 10.3390/ijms25042070. 10.3390/ijms25042070 PMC10889139 38396746

[23] Purnomosari D, Nabila BZ, Widyarini S, Mustofa. Targeting immune cells in tumor microenvironment in triple negative breast cancer therapy: future perspective to overcome doxorubicin resistance and toxicity. Med Oncol. 2025;42(5):150. 10.1007/s12032-025-02712-6. 40183881 10.1007/s12032-025-02712-6

[24] Porkka K, Blomqvist C, Rissanen P, Elomaa I, Pyrhonen S. Salvage therapies in women who fail to respond to first-line treatment with fluorouracil, epirubicin, and cyclophosphamide for advanced breast cancer. J Clin Oncol. 1994;12(8):1639–47. 10.1200/JCO.1994.12.8.1639. 8040676 10.1200/JCO.1994.12.8.1639

[25] Anurag M, Jaehnig EJ, Krug K, Lei JT, Bergstrom EJ, Kim BJ, et al. Proteogenomic markers of chemotherapy resistance and response in triple-negative breast cancer. Cancer Discov. 2022;12(11):2586–605. 10.1158/2159-8290.CD-22-0200. 36001024 10.1158/2159-8290.CD-22-0200 PMC9627136

[26] Blondy S, David V, Verdier M, Mathonnet M, Perraud A, Christou N. 5-Fluorouracil resistance mechanisms in colorectal cancer: from classical pathways to promising processes. Cancer Sci. 2020;111(9):3142–54. 10.1111/cas.14532. 32536012 10.1111/cas.14532 PMC7469786

[27] Yuan J, Khan SU, Yan J, Lu J, Yang C, Tong Q. Baicalin enhances the efficacy of 5-fluorouracil in gastric cancer by promoting ROS-mediated ferroptosis. Biomed Pharmacother. 2023;164:114986. 10.1016/j.biopha.2023.114986. 37295251 10.1016/j.biopha.2023.114986

[28] Hashemi M, Esbati N, Rashidi M, Gholami S, Raesi R, Bidoki SS, et al. Biological landscape and nanostructural view in development and reversal of oxaliplatin resistance in colorectal cancer. Transl Oncol. 2024;40:101846. 10.1016/j.tranon.2023.101846. 38042134 10.1016/j.tranon.2023.101846 PMC10716031

[29] Harada K, Sakamoto N, Ukai S, Yamamoto Y, Pham QT, Taniyama D, et al. Establishment of oxaliplatin-resistant gastric cancer organoids: importance of myoferlin in the acquisition of oxaliplatin resistance. Gastric Cancer. 2021;24(6):1264–77. 10.1007/s10120-021-01206-4. 34272617 10.1007/s10120-021-01206-4

[30] Masucci MT, Motti ML, Minopoli M, Di Carluccio G, Carriero MV. Emerging targeted therapeutic strategies to overcome imatinib resistance of gastrointestinal stromal tumors. Int J Mol Sci. 2023. 10.3390/ijms24076026. 37046997 10.3390/ijms24076026 PMC10094678

[31] Seidman AD, Fornier MN, Esteva FJ, Tan L, Kaptain S, Bach A, et al. Weekly trastuzumab and paclitaxel therapy for metastatic breast cancer with analysis of efficacy by HER2 immunophenotype and gene amplification. J Clin Oncol. 2001;19(10):2587–95. 10.1200/JCO.2001.19.10.2587. 11352950 10.1200/JCO.2001.19.10.2587

[32] Topalian SL, Hodi FS, Brahmer JR, Gettinger SN, Smith DC, McDermott DF, et al. Five-year survival and correlates among patients with advanced melanoma, renal cell carcinoma, or non-small cell lung cancer treated with nivolumab. JAMA Oncol. 2019;5(10):1411–20. 10.1001/jamaoncol.2019.2187. 31343665 10.1001/jamaoncol.2019.2187 PMC6659167

[33] Raja FA, Counsell N, Colombo N, Pfisterer J, du Bois A, Parmar MK, et al. Platinum versus platinum-combination chemotherapy in platinum-sensitive recurrent ovarian cancer: a meta-analysis using individual patient data. Ann Oncol. 2013;24(12):3028–34. 10.1093/annonc/mdt406. 24190964 10.1093/annonc/mdt406

[34] Larkin J, Chiarion-Sileni V, Gonzalez R, Grob JJ, Cowey CL, Lao CD, et al. Combined nivolumab and ipilimumab or monotherapy in untreated melanoma. N Engl J Med. 2015;373(1):23–34. 10.1056/NEJMoa1504030. 26027431 10.1056/NEJMoa1504030 PMC5698905

[35] Enriquez-Navas PM, Kam Y, Das T, Hassan S, Silva A, Foroutan P, et al. Exploiting evolutionary principles to prolong tumor control in preclinical models of breast cancer. Sci Transl Med. 2016;8(327):327ra24. 10.1126/scitranslmed.aad7842. 26912903 10.1126/scitranslmed.aad7842 PMC4962860

[36] Lu J, Li J, Lin Z, Li H, Lou L, Ding W, et al. Reprogramming of TAMs via the STAT3/CD47-SIRPα axis promotes acquired resistance to EGFR-TKIs in lung cancer. Cancer Lett. 2023;564:216205. 10.1016/j.canlet.2023.216205. 37146936 10.1016/j.canlet.2023.216205

[37] Shi ZD, Pang K, Wu ZX, Dong Y, Hao L, Qin JX, et al. Tumor cell plasticity in targeted therapy-induced resistance: mechanisms and new strategies. Signal Transduct Target Ther. 2023;8(1):113. 10.1038/s41392-023-01383-x. 36906600 10.1038/s41392-023-01383-x PMC10008648

[38] Lim ZF, Ma PC. Emerging insights of tumor heterogeneity and drug resistance mechanisms in lung cancer targeted therapy. J Hematol Oncol. 2019;12(1):134. 10.1186/s13045-019-0818-2. 31815659 10.1186/s13045-019-0818-2 PMC6902404

[39] Hass R, von der Ohe J, Ungefroren H. Impact of the Tumor Microenvironment on Tumor Heterogeneity and Consequences for Cancer Cell Plasticity and Stemness. Cancers (Basel). 2020;12(12). 10.3390/cancers12123716. 10.3390/cancers12123716 PMC7764513 33322354

[40] Boumahdi S, de Sauvage FJ. The great escape: tumour cell plasticity in resistance to targeted therapy. Nat Rev Drug Discov. 2020;19(1):39–56. 10.1038/s41573-019-0044-1. 31601994 10.1038/s41573-019-0044-1

[41] Dong RF, Zhu ML, Liu MM, Xu YT, Yuan LL, Bian J, et al. EGFR mutation mediates resistance to EGFR tyrosine kinase inhibitors in NSCLC: from molecular mechanisms to clinical research. Pharmacol Res. 2021;167:105583. 10.1016/j.phrs.2021.105583. 33775864 10.1016/j.phrs.2021.105583

[42] Fu K, Xie F, Wang F, Fu L. Therapeutic strategies for EGFR-mutated non-small cell lung cancer patients with osimertinib resistance. J Hematol Oncol. 2022;15(1):173. 10.1186/s13045-022-01391-4. 36482474 10.1186/s13045-022-01391-4 PMC9733018

[43] Westover D, Zugazagoitia J, Cho BC, Lovly CM, Paz-Ares L. Mechanisms of acquired resistance to first- and second-generation EGFR tyrosine kinase inhibitors. Ann Oncol. 2018;29(suppl_1):i10–9. 10.1093/annonc/mdx703. 29462254 10.1093/annonc/mdx703 PMC6454547

[44] Nishiyama A, Takeuchi S, Adachi Y, Otani S, Tanimoto A, Sasaki M, et al. MET amplification results in heterogeneous responses to osimertinib in EGFR-mutant lung cancer treated with erlotinib. Cancer Sci. 2020;111(10):3813–23. 10.1111/cas.14593. 32735723 10.1111/cas.14593 PMC7540985

[45] Planchard D, Loriot Y, Andre F, Gobert A, Auger N, Lacroix L, et al. EGFR-independent mechanisms of acquired resistance to AZD9291 in EGFR T790M-positive NSCLC patients. Ann Oncol. 2015;26(10):2073–8. 10.1093/annonc/mdv319. 26269204 10.1093/annonc/mdv319

[46] Park W, Wei S, Xie CL, Han JH, Kim BS, Kim B, et al. Targeting pyruvate dehydrogenase kinase 1 overcomes EGFR C797S mutation-driven osimertinib resistance in non-small cell lung cancer. Exp Mol Med. 2024;56(5):1137–49. 10.1038/s12276-024-01221-2. 38689087 10.1038/s12276-024-01221-2 PMC11148081

[47] Hsu SK, Jadhao M, Liao WT, Chang WT, Hung CT, Chiu CC. Culprits of PDAC resistance to gemcitabine and immune checkpoint inhibitor: tumour microenvironment components. Front Mol Biosci. 2022;9:1020888. 10.3389/fmolb.2022.1020888. 36299300 10.3389/fmolb.2022.1020888 PMC9589289

[48] Rebelo R, Xavier CPR, Giovannetti E, Vasconcelos MH. Fibroblasts in pancreatic cancer: molecular and clinical perspectives. Trends Mol Med. 2023;29(6):439–53. 10.1016/j.molmed.2023.03.002. 37100646 10.1016/j.molmed.2023.03.002

[49] Cannone S, Greco MR, Carvalho TMA, Guizouarn H, Soriani O, Di Molfetta D, et al. Cancer Associated Fibroblast (CAF) Regulation of PDAC Parenchymal (CPC) and CSC Phenotypes Is Modulated by ECM Composition. Cancers (Basel). 2022;14(15). 10.3390/cancers14153737. 10.3390/cancers14153737 PMC9367491 35954400

[50] Liebner S, Dijkhuizen RM, Reiss Y, Plate KH, Agalliu D, Constantin G. Functional morphology of the blood-brain barrier in health and disease. Acta Neuropathol. 2018;135(3):311–36. 10.1007/s00401-018-1815-1. 29411111 10.1007/s00401-018-1815-1 PMC6781630

[51] Gomez-Zepeda D, Taghi M, Scherrmann JM, Decleves X, Menet MC. ABC Transporters at the Blood-Brain Interfaces, Their Study Models, and Drug Delivery Implications in Gliomas. Pharmaceutics. 2019;12(1). 10.3390/pharmaceutics12010020. 10.3390/pharmaceutics12010020 PMC7022905 31878061

[52] Pan YL, Zeng SX, Hao RR, Liang MH, Shen ZR, Huang WH. The progress of small-molecules and degraders against BCR-ABL for the treatment of CML. Eur J Med Chem. 2022;238:114442. 10.1016/j.ejmech.2022.114442. 35551036 10.1016/j.ejmech.2022.114442

[53] El-Tanani M, Nsairat H, Matalka, II, Lee YF, Rizzo M, Aljabali AA, et al. The impact of the BCR-ABL oncogene in the pathology and treatment of chronic myeloid leukemia. Pathol Res Pract. 2024;254(155161). 10.1016/j.prp.2024.155161. 10.1016/j.prp.2024.155161 38280275

[54] Aksoy O, Lind J, Sunder-Plassmann V, Vallet S, Podar K. Bone marrow microenvironment- induced regulation of Bcl-2 family members in multiple myeloma (MM): therapeutic implications. Cytokine. 2023;161:156062. 10.1016/j.cyto.2022.156062. 36332463 10.1016/j.cyto.2022.156062

[55] Bhowmick K, von Suskil M, Al-Odat OS, Elbezanti WO, Jonnalagadda SC, Budak-Alpdogan T, et al. Pathways to therapy resistance: the sheltering effect of the bone marrow microenvironment to multiple myeloma cells. Heliyon. 2024;10(12):e33091. 10.1016/j.heliyon.2024.e33091. 39021902 10.1016/j.heliyon.2024.e33091 PMC11252793

[56] Leonetti A, Sharma S, Minari R, Perego P, Giovannetti E, Tiseo M. Resistance mechanisms to osimertinib in EGFR-mutated non-small cell lung cancer. Br J Cancer. 2019;121(9):725–37. 10.1038/s41416-019-0573-8. 31564718 10.1038/s41416-019-0573-8 PMC6889286

[57] Cooper AJ, Sequist LV, Lin JJ. Third-generation EGFR and ALK inhibitors: mechanisms of resistance and management. Nat Rev Clin Oncol. 2022;19(8):499–514. 10.1038/s41571-022-00639-9. 35534623 10.1038/s41571-022-00639-9 PMC9621058

[58] Guaitoli G, Bertolini F, Bettelli S, Manfredini S, Maur M, Trudu L, et al. Deepening the Knowledge of ROS1 Rearrangements in Non-Small Cell Lung Cancer: Diagnosis, Treatment, Resistance and Concomitant Alterations. Int J Mol Sci. 2021;22(23). 10.3390/ijms222312867.. 10.3390/ijms222312867 PMC8657497 34884672

[59] Rivas S, Marin A, Samtani S, Gonzalez-Feliu E, Armisen R. MET Signaling Pathways, Resistance Mechanisms, and Opportunities for Target Therapies. Int J Mol Sci. 2022;23(22). 10.3390/ijms232213898. 10.3390/ijms232213898 PMC9697723 36430388

[60] Wang ZH, Zheng ZQ, Jia SC, Liu SN, Xiao XF, Chen GY, et al. Trastuzumab resistance in HER2-positive breast cancer: mechanisms, emerging biomarkers and targeting agents. Front Oncol. 2022;12:1006429. 10.3389/fonc.2022.1006429. 36276152 10.3389/fonc.2022.1006429 PMC9584623

[61] Di Federico A, Ricciotti I, Favorito V, Michelina SV, Scaparone P, Metro G, et al. Resistance to KRAS G12C inhibition in non-small cell lung cancer. Curr Oncol Rep. 2023;25(9):1017–29. 10.1007/s11912-023-01436-y. 37378881 10.1007/s11912-023-01436-y

[62] Kun E, Tsang YTM, Ng CW, Gershenson DM, Wong KK. MEK inhibitor resistance mechanisms and recent developments in combination trials. Cancer Treat Rev. 2021;92:102137. 10.1016/j.ctrv.2020.102137. 33340965 10.1016/j.ctrv.2020.102137

[63] Shah FH, Nam YS, Bang JY, Hwang IS, Kim DH, Ki M, et al. Targeting vascular endothelial growth receptor-2 (VEGFR-2): structural biology, functional insights, and therapeutic resistance. Arch Pharm Res. 2025;48(5):404–25. 10.1007/s12272-025-01545-1. 40341988 10.1007/s12272-025-01545-1 PMC12106596

[64] Toledo RA, Jimenez C, Armaiz-Pena G, Arenillas C, Capdevila J, Dahia PLM. Hypoxia-Inducible Factor 2 Alpha (HIF2alpha) Inhibitors: Targeting Genetically Driven Tumor Hypoxia. Endocr Rev. 2023;44(2):312–22. 10.1210/endrev/bnac025. 36301191 10.1210/endrev/bnac025 PMC10216878

[65] Dias MP, Moser SC, Ganesan S, Jonkers J. Understanding and overcoming resistance to PARP inhibitors in cancer therapy. Nat Rev Clin Oncol. 2021;18(12):773–91. 10.1038/s41571-021-00532-x. 34285417 10.1038/s41571-021-00532-x

[66] Baxter JS, Zatreanu D, Pettitt SJ, Lord CJ. Resistance to DNA repair inhibitors in cancer. Mol Oncol. 2022;16(21):3811–27. 10.1002/1878-0261.13224. 35567571 10.1002/1878-0261.13224 PMC9627783

[67] Zhuang X, Pei HZ, Li T, Huang J, Guo Y, Zhao Y, et al. The molecular mechanisms of resistance to IDH inhibitors in acute myeloid leukemia. Front Oncol. 2022;12:931462. 10.3389/fonc.2022.931462. 35814406 10.3389/fonc.2022.931462 PMC9260655

[68] Wang ZQ, Zhang ZC, Wu YY, Pi YN, Lou SH, Liu TB, et al. Bromodomain and extraterminal (BET) proteins: biological functions, diseases, and targeted therapy. Signal Transduct Target Ther. 2023;8(1):420. 10.1038/s41392-023-01647-6. 37926722 10.1038/s41392-023-01647-6 PMC10625992

[69] Huang J, Zheng L, Sun Z, Li J. CDK4/6 inhibitor resistance mechanisms and treatment strategies (Review). Int J Mol Med. 2022. 10.3892/ijmm.2022.5184. 36043521 10.3892/ijmm.2022.5184 PMC9448295

[70] Lei Q, Wang D, Sun K, Wang L, Zhang Y. Resistance mechanisms of anti-PD1/PDL1 therapy in solid tumors. Front Cell Dev Biol. 2020;8:672. 10.3389/fcell.2020.00672. 32793604 10.3389/fcell.2020.00672 PMC7385189

[71] Rigakos G, Razis E, Koliou GA, Oikonomopoulos G, Tsolaki E, Sperinde J, et al. Evaluation of the role of p95 HER2 isoform in trastuzumab efficacy in metastatic breast cancer. Anticancer Res. 2021;41(4):1793–802. 10.21873/anticanres.14945. 33813384 10.21873/anticanres.14945

[72] Zhang H, Zhang L, He Y, Jiang D, Sun J, Luo Q, et al. PI3K PROTAC overcomes the lapatinib resistance in PIK3CA-mutant HER2 positive breast cancer. Cancer Lett. 2024;598:217112. 10.1016/j.canlet.2024.217112. 38986734 10.1016/j.canlet.2024.217112

[73] Wolf J, Hochmair M, Han JY, Reguart N, Souquet PJ, Smit EF, et al. Capmatinib in MET exon 14-mutated non-small-cell lung cancer: final results from the open-label, phase 2 GEOMETRY mono-1 trial. Lancet Oncol. 2024;25(10):1357–70. 10.1016/S1470-2045(24)00441-8. 39362249 10.1016/S1470-2045(24)00441-8

[74] Zhang Y, Shen L, Peng Z. Advances in MET tyrosine kinase inhibitors in gastric cancer. Cancer Biol Med. 2024;21(6):484–98. 10.20892/j.issn.2095-3941.2024.0044. 38727001 10.20892/j.issn.2095-3941.2024.0044 PMC11208904

[75] Kim S, Kim TM, Kim DW, Kim S, Kim M, Ahn YO, et al. Acquired resistance of MET-amplified non-small cell lung cancer cells to the MET inhibitor capmatinib. Cancer Res Treat. 2019;51(3):951–62. 10.4143/crt.2018.052. 30309221 10.4143/crt.2018.052 PMC6639226

[76] Han R, Guo H, Shi J, Wang H, Zhao S, Jia Y, et al. Tumour microenvironment changes after osimertinib treatment resistance in non-small cell lung cancer. Eur J Cancer. 2023;189:112919. 10.1016/j.ejca.2023.05.007. 37320935 10.1016/j.ejca.2023.05.007

[77] Zhang J, Huang D, Saw PE, Song E. Turning cold tumors hot: from molecular mechanisms to clinical applications. Trends Immunol. 2022;43(7):523–45. 10.1016/j.it.2022.04.010. 35624021 10.1016/j.it.2022.04.010

[78] Rai V. Immune Checkpoint Inhibitor Therapy for Prostate Cancer: Present and Future Prospectives. Biomolecules. 2025;15(6). 10.3390/biom15060751. 10.3390/biom15060751 PMC12190701 40563393

[79] Sridaran D, Bradshaw E, DeSelm C, Pachynski R, Mahajan K, Mahajan NP. Prostate cancer immunotherapy: improving clinical outcomes with a multi-pronged approach. Cell Rep Med. 2023;4(10):101199. 10.1016/j.xcrm.2023.101199. 37738978 10.1016/j.xcrm.2023.101199 PMC10591038

[80] Meng J, Jiang A, Lu X, Gu D, Ge Q, Bai S, et al. Multiomics characterization and verification of clear cell renal cell carcinoma molecular subtypes to guide precise chemotherapy and immunotherapy. Imeta. 2023;2(4):e147. 10.1002/imt2.147. 38868222 10.1002/imt2.147 PMC10989995

[81] Memon D, Schoenfeld AJ, Ye D, Fromm G, Rizvi H, Zhang X, et al. Clinical and molecular features of acquired resistance to immunotherapy in non-small cell lung cancer. Cancer Cell. 2024;42(2):209-24 e9. 10.1016/j.ccell.2023.12.013. 38215748 10.1016/j.ccell.2023.12.013 PMC11249385

[82] Schachter J, Ribas A, Long GV, Arance A, Grob JJ, Mortier L, et al. Pembrolizumab versus ipilimumab for advanced melanoma: final overall survival results of a multicentre, randomised, open-label phase 3 study (KEYNOTE-006). Lancet. 2017;390(10105):1853–62. 10.1016/S0140-6736(17)31601-X. 28822576 10.1016/S0140-6736(17)31601-X

[83] Agrawal V, Benjamin KT, Ko EC. Radiotherapy and immunotherapy combinations for lung cancer. Curr Oncol Rep. 2020;23(1):4. 10.1007/s11912-020-00993-w. 33215306 10.1007/s11912-020-00993-w

[84] Burris HA 3rd, Rugo HS, Vukelja SJ, Vogel CL, Borson RA, Limentani S, et al. Phase II study of the antibody drug conjugate trastuzumab-DM1 for the treatment of human epidermal growth factor receptor 2 (HER2)-positive breast cancer after prior HER2-directed therapy. J Clin Oncol. 2011;29(4):398–405. 10.1200/JCO.2010.29.5865. 21172893 10.1200/JCO.2010.29.5865

[85] Yamazaki CM, Yamaguchi A, Anami Y, Xiong W, Otani Y, Lee J, et al. Antibody-drug conjugates with dual payloads for combating breast tumor heterogeneity and drug resistance. Nat Commun. 2021;12(1):3528. 10.1038/s41467-021-23793-7. 34112795 10.1038/s41467-021-23793-7 PMC8192907

[86] Baldassarre T, Truesdell P, Craig AW. Endophilin A2 promotes HER2 internalization and sensitivity to trastuzumab-based therapy in HER2-positive breast cancers. Breast Cancer Res. 2017;19(1):110. 10.1186/s13058-017-0900-z. 28974266 10.1186/s13058-017-0900-z PMC5627411

[87] Sung M, Tan X, Lu B, Golas J, Hosselet C, Wang F, et al. Caveolae-mediated endocytosis as a novel mechanism of resistance to Trastuzumab emtansine (T-DM1). Mol Cancer Ther. 2018;17(1):243–53. 10.1158/1535-7163.MCT-17-0403. 29054985 10.1158/1535-7163.MCT-17-0403

[88] Rios-Luci C, Garcia-Alonso S, Diaz-Rodriguez E, Nadal-Serrano M, Arribas J, Ocana A, et al. Resistance to the antibody-drug conjugate T-DM1 is based in a reduction in lysosomal proteolytic activity. Cancer Res. 2017;77(17):4639–51. 10.1158/0008-5472.CAN-16-3127. 28687619 10.1158/0008-5472.CAN-16-3127

[89] Wang H, Wang W, Xu Y, Yang Y, Chen X, Quan H, et al. Aberrant intracellular metabolism of T-DM1 confers T-DM1 resistance in human epidermal growth factor receptor 2-positive gastric cancer cells. Cancer Sci. 2017;108(7):1458–68. 10.1111/cas.13253. 28388007 10.1111/cas.13253 PMC5497802

[90] Yu SF, Zheng B, Go M, Lau J, Spencer S, Raab H, et al. A novel anti-CD22 anthracycline-based antibody-drug conjugate (ADC) that overcomes resistance to auristatin-based ADCs. Clin Cancer Res. 2015;21(14):3298–306. 10.1158/1078-0432.CCR-14-2035. 25840969 10.1158/1078-0432.CCR-14-2035

[91] Coates JT, Sun S, Leshchiner I, Thimmiah N, Martin EE, McLoughlin D, et al. Parallel Genomic Alterations of Antigen and Payload Targets Mediate Polyclonal Acquired Clinical Resistance to Sacituzumab Govitecan in Triple-Negative Breast Cancer. Cancer Discov. 2021;11(10):2436–45. 10.1158/2159-8290.CD-21-0702. 34404686 10.1158/2159-8290.CD-21-0702 PMC8495771

[92] Nussinov R, Tsai CJ, Jang H. Anticancer drug resistance: an update and perspective. Drug Resist Updat. 2021;59:100796. 10.1016/j.drup.2021.100796. 34953682 10.1016/j.drup.2021.100796 PMC8810687

[93] Khalaf K, Hana D, Chou JT, Singh C, Mackiewicz A, Kaczmarek M. Aspects of the tumor microenvironment involved in immune resistance and drug resistance. Front Immunol. 2021;12:656364. 10.3389/fimmu.2021.656364. 34122412 10.3389/fimmu.2021.656364 PMC8190405

[94] Dagogo-Jack I, Shaw AT. Tumour heterogeneity and resistance to cancer therapies. Nat Rev Clin Oncol. 2018;15(2):81–94. 10.1038/nrclinonc.2017.166. 29115304 10.1038/nrclinonc.2017.166

[95] Marusyk A, Janiszewska M, Polyak K. Intratumor heterogeneity: the Rosetta stone of therapy resistance. Cancer Cell. 2020;37(4):471–84. 10.1016/j.ccell.2020.03.007. 32289271 10.1016/j.ccell.2020.03.007 PMC7181408

[96] Nam AS, Chaligne R, Landau DA. Integrating genetic and non-genetic determinants of cancer evolution by single-cell multi-omics. Nat Rev Genet. 2021;22(1):3–18. 10.1038/s41576-020-0265-5. 32807900 10.1038/s41576-020-0265-5 PMC8450921

[97] Shklovskaya E, Rizos H. Spatial and Temporal Changes in PD-L1 Expression in Cancer: The Role of Genetic Drivers, Tumor Microenvironment and Resistance to Therapy. Int J Mol Sci. 2020;21(19). 10.3390/ijms21197139. 10.3390/ijms21197139 PMC7583014 32992658

[98] Ma X, Xu J, Wang Y, Fleishman JS, Bing H, Yu B, et al. Research progress on gene mutations and drug resistance in leukemia. Drug Resist Updat. 2025;79:101195. 10.1016/j.drup.2024.101195. 39740374 10.1016/j.drup.2024.101195

[99] Qin K, Hong L, Zhang J, Le X. MET Amplification as a Resistance Driver to TKI Therapies in Lung Cancer: Clinical Challenges and Opportunities. Cancers (Basel). 2023;15(3). 10.3390/cancers15030612. 10.3390/cancers15030612 PMC9913224 36765572

[100] Cocco E, Scaltriti M, Drilon A. NTRK fusion-positive cancers and TRK inhibitor therapy. Nat Rev Clin Oncol. 2018;15(12):731–47. 10.1038/s41571-018-0113-0. 30333516 10.1038/s41571-018-0113-0 PMC6419506

[101] Bukowski K, Kciuk M, Kontek R. Mechanisms of Multidrug Resistance in Cancer Chemotherapy. Int J Mol Sci. 2020;21(9). 10.3390/ijms21093233. 10.3390/ijms21093233 PMC7247559 32370233

[102] Hu J, Cao J, Topatana W, Juengpanich S, Li S, Zhang B, et al. Targeting mutant p53 for cancer therapy: direct and indirect strategies. J Hematol Oncol. 2021;14(1):157. 10.1186/s13045-021-01169-0. 34583722 10.1186/s13045-021-01169-0 PMC8480024

[103] Hientz K, Mohr A, Bhakta-Guha D, Efferth T. The role of p53 in cancer drug resistance and targeted chemotherapy. Oncotarget. 2017;8(5):8921–46. 10.18632/oncotarget.13475. 27888811 10.18632/oncotarget.13475 PMC5352454

[104] Robey RW, Pluchino KM, Hall MD, Fojo AT, Bates SE, Gottesman MM. Revisiting the role of ABC transporters in multidrug-resistant cancer. Nat Rev Cancer. 2018;18(7):452–64. 10.1038/s41568-018-0005-8. 29643473 10.1038/s41568-018-0005-8 PMC6622180

[105] Roninson IB. The role of the MDR1 (P-glycoprotein) gene in multidrug resistance *in vitro* and *in vivo*. Biochem Pharmacol. 1992;43(1):95–102. 10.1016/0006-2952(92)90666-7. 1346497 10.1016/0006-2952(92)90666-7

[106] Jensen NF, Agama K, Roy A, Smith DH, Pfister TD, Romer MU, et al. Characterization of DNA topoisomerase I in three SN-38 resistant human colon cancer cell lines reveals a new pair of resistance-associated mutations. J Exp Clin Cancer Res. 2016;35(56). 10.1186/s13046-016-0335-x. 10.1186/s13046-016-0335-x PMC4815242 27029323

[107] Gongora C, Vezzio-Vie N, Tuduri S, Denis V, Causse A, Auzanneau C, et al. New topoisomerase I mutations are associated with resistance to camptothecin. Mol Cancer. 2011;10:64. 10.1186/1476-4598-10-64. 21619602 10.1186/1476-4598-10-64 PMC3120799

[108] Kavallaris M. Microtubules and resistance to tubulin-binding agents. Nat Rev Cancer. 2010;10(3):194–204. 10.1038/nrc2803. 20147901 10.1038/nrc2803

[109] Smith ER, Wang JQ, Yang DH, Xu XX. Paclitaxel resistance related to nuclear envelope structural sturdiness. Drug Resist Updat. 2022;65:100881. 10.1016/j.drup.2022.100881. 36368286 10.1016/j.drup.2022.100881

[110] Soragni A, Knudsen ES, O’Connor TN, Tognon CE, Tyner JW, Gini B, et al. Acquired resistance in cancer: towards targeted therapeutic strategies. Nat Rev Cancer. 2025;25(8):613–33. 10.1038/s41568-025-00824-9. 40461793 10.1038/s41568-025-00824-9 PMC12307123

[111] Quintas-Cardama A, Cortes J. Therapeutic options against BCR-ABL1 T315I-positive chronic myelogenous leukemia. Clin Cancer Res. 2008;14(14):4392–9. 10.1158/1078-0432.CCR-08-0117. 18628453 10.1158/1078-0432.CCR-08-0117

[112] Braun TP, Eide CA, Druker BJ. Response and resistance to BCR-ABL1-targeted therapies. Cancer Cell. 2020;37(4):530–42. 10.1016/j.ccell.2020.03.006. 32289275 10.1016/j.ccell.2020.03.006 PMC7722523

[113] Haratani K, Hayashi H, Tanaka T, Kaneda H, Togashi Y, Sakai K, et al. Tumor immune microenvironment and nivolumab efficacy in EGFR mutation-positive non-small-cell lung cancer based on T790M status after disease progression during EGFR-TKI treatment. Ann Oncol. 2017;28(7):1532–9. 10.1093/annonc/mdx183. 28407039 10.1093/annonc/mdx183

[114] Jia Y, Yun CH, Park E, Ercan D, Manuia M, Juarez J, et al. Overcoming EGFR(T790M) and EGFR(C797S) resistance with mutant-selective allosteric inhibitors. Nature. 2016;534(7605):129–32. 10.1038/nature17960. 27251290 10.1038/nature17960 PMC4929832

[115] Sasaki T, Okuda K, Zheng W, Butrynski J, Capelletti M, Wang L, et al. The neuroblastoma-associated F1174L ALK mutation causes resistance to an ALK kinase inhibitor in ALK-translocated cancers. Cancer Res. 2010;70(24):10038–43. 10.1158/0008-5472.CAN-10-2956. 21030459 10.1158/0008-5472.CAN-10-2956 PMC3045808

[116] Dagogo-Jack I, Shaw AT. Crizotinib resistance: implications for therapeutic strategies. Ann Oncol. 2016;27 Suppl 3(Suppl 3):iii42–50. 10.1093/annonc/mdw305. 27573756 10.1093/annonc/mdw305 PMC5003168

[117] Liu X, Mei W, Zhang P, Zeng C. *PIK3CA* mutation as an acquired resistance driver to *EGFR-TKIs* in non-small cell lung cancer: clinical challenges and opportunities. Pharmacol Res. 2024;202:107123. 10.1016/j.phrs.2024.107123. 38432445 10.1016/j.phrs.2024.107123

[118] Rasti AR, Guimaraes-Young A, Datko F, Borges VF, Aisner DL, Shagisultanova E. PIK3CA Mutations Drive Therapeutic Resistance in Human Epidermal Growth Factor Receptor 2-Positive Breast Cancer. JCO Precis Oncol. 2022;6:e2100370. 10.1200/PO.21.00370. 35357905 10.1200/PO.21.00370 PMC8984255

[119] Juric D, Castel P, Griffith M, Griffith OL, Won HH, Ellis H, et al. Convergent loss of PTEN leads to clinical resistance to a PI(3)Kalpha inhibitor. Nature. 2015;518(7538):240–4. 10.1038/nature13948. 25409150 10.1038/nature13948 PMC4326538

[120] Lin JJ, Choudhury NJ, Yoda S, Zhu VW, Johnson TW, Sakhtemani R, et al. Spectrum of mechanisms of resistance to crizotinib and lorlatinib in ROS1 fusion-positive lung cancer. Clin Cancer Res. 2021;27(10):2899–909. 10.1158/1078-0432.CCR-21-0032. 33685866 10.1158/1078-0432.CCR-21-0032 PMC8127383

[121] Lin C, Ren Z, Yang X, Yang R, Chen Y, Liu Z, et al. Nerve growth factor (NGF)-TrkA axis in head and neck squamous cell carcinoma triggers EMT and confers resistance to the EGFR inhibitor erlotinib. Cancer Lett. 2020;472:81–96. 10.1016/j.canlet.2019.12.015. 31838083 10.1016/j.canlet.2019.12.015

[122] Deng S, Wang C, Wang Y, Xu Y, Li X, Johnson NA, et al. Ectopic JAK-STAT activation enables the transition to a stem-like and multilineage state conferring AR-targeted therapy resistance. Nat Cancer. 2022;3(9):1071–87. 10.1038/s43018-022-00431-9. 36065066 10.1038/s43018-022-00431-9 PMC9499870

[123] Zaretsky JM, Garcia-Diaz A, Shin DS, Escuin-Ordinas H, Hugo W, Hu-Lieskovan S, et al. Mutations associated with acquired resistance to PD-1 blockade in melanoma. N Engl J Med. 2016;375(9):819–29. 10.1056/NEJMoa1604958. 27433843 10.1056/NEJMoa1604958 PMC5007206

[124] Rizvi NA, Hellmann MD, Snyder A, Kvistborg P, Makarov V, Havel JJ, et al. Cancer immunology. Mutational landscape determines sensitivity to PD-1 blockade in non-small cell lung cancer. Science. 2015;348(6230):124–8. 10.1126/science.aaa1348. 25765070 10.1126/science.aaa1348 PMC4993154

[125] Shin DS, Zaretsky JM, Escuin-Ordinas H, Garcia-Diaz A, Hu-Lieskovan S, Kalbasi A, et al. Primary resistance to PD-1 blockade mediated by JAK1/2 mutations. Cancer Discov. 2017;7(2):188–201. 10.1158/2159-8290.CD-16-1223. 27903500 10.1158/2159-8290.CD-16-1223 PMC5296316

[126] IFNgamma Mutations Prompt CTLA-4 Inhibitor Resistance. Cancer Discov. 2017;7(1):OF3. 10.1158/2159-8290.CD-NB2016-148. 10.1158/2159-8290.CD-NB2016-148 27864228

[127] Gao J, Shi LZ, Zhao H, Chen J, Xiong L, He Q, et al. Loss of IFN-gamma Pathway Genes in Tumor Cells as a Mechanism of Resistance to Anti-CTLA-4 Therapy. Cell. 2016;167(2):397-404 e9. 10.1016/j.cell.2016.08.069. 27667683 10.1016/j.cell.2016.08.069 PMC5088716

[128] Ge LP, Jin X, Ma D, Wang ZY, Liu CL, Zhou CZ, et al. ZNF689 deficiency promotes intratumor heterogeneity and immunotherapy resistance in triple-negative breast cancer. Cell Res. 2024;34(1):58–75. 10.1038/s41422-023-00909-w. 38168642 10.1038/s41422-023-00909-w PMC10770380

[129] Deng P, Wang Z, Chen J, Liu S, Yao X, Liu S, et al. RAD21 amplification epigenetically suppresses interferon signaling to promote immune evasion in ovarian cancer. J Clin Invest. 2022;132(22). 10.1172/JCI159628. 10.1172/JCI159628 PMC9663158 36201246

[130] Adhikari S, Bhattacharya A, Adhikary S, Singh V, Gadad SS, Roy S, et al. The paradigm of drug resistance in cancer: an epigenetic perspective. Biosci Rep. 2022;42(4). 10.1042/BSR20211812. 10.1042/BSR20211812 PMC9069444 35438143

[131] Ravindran Menon D, Hammerlindl H, Torrano J, Schaider H, Fujita M. Epigenetics and metabolism at the crossroads of stress-induced plasticity, stemness and therapeutic resistance in cancer. Theranostics. 2020;10(14):6261–77. 10.7150/thno.42523. 32483452 10.7150/thno.42523 PMC7255038

[132] Dawson MA, Kouzarides T. Cancer epigenetics: from mechanism to therapy. Cell. 2012;150(1):12–27. 10.1016/j.cell.2012.06.013. 22770212 10.1016/j.cell.2012.06.013

[133] Lee TI, Young RA. Transcriptional regulation and its misregulation in disease. Cell. 2013;152(6):1237–51. 10.1016/j.cell.2013.02.014. 23498934 10.1016/j.cell.2013.02.014 PMC3640494

[134] Aydin B, Mazzoni EO. Cell reprogramming: the many roads to success. Annu Rev Cell Dev Biol. 2019;35:433. 10.1146/annurev-cellbio-100818-125127. 31340126 10.1146/annurev-cellbio-100818-125127

[135] Poetsch AR, Plass C. Transcriptional regulation by DNA methylation. Cancer Treat Rev. 2011;37(Suppl 1):S8-12. 10.1016/j.ctrv.2011.04.010. 21601364 10.1016/j.ctrv.2011.04.010

[136] Zhao LY, Song J, Liu Y, Song CX, Yi C. Mapping the epigenetic modifications of DNA and RNA. Protein Cell. 2020;11(11):792–808. 10.1007/s13238-020-00733-7. 32440736 10.1007/s13238-020-00733-7 PMC7647981

[137] Easwaran H, Tsai HC, Baylin SB. Cancer epigenetics: tumor heterogeneity, plasticity of stem-like states, and drug resistance. Mol Cell. 2014;54(5):716–27. 10.1016/j.molcel.2014.05.015. 24905005 10.1016/j.molcel.2014.05.015 PMC4103691

[138] Cao J, Yan Q. Cancer epigenetics, tumor immunity, and immunotherapy. Trends Cancer. 2020;6(7):580–92. 10.1016/j.trecan.2020.02.003. 32610068 10.1016/j.trecan.2020.02.003 PMC7330177

[139] Hogg SJ, Beavis PA, Dawson MA, Johnstone RW. Targeting the epigenetic regulation of antitumour immunity. Nat Rev Drug Discov. 2020;19(11):776–800. 10.1038/s41573-020-0077-5. 32929243 10.1038/s41573-020-0077-5

[140] Dai E, Zhu Z, Wahed S, Qu Z, Storkus WJ, Guo ZS. Epigenetic modulation of antitumor immunity for improved cancer immunotherapy. Mol Cancer. 2021;20(1):171. 10.1186/s12943-021-01464-x. 34930302 10.1186/s12943-021-01464-x PMC8691037

[141] Micevic G, Bosenberg MW, Yan Q. The crossroads of cancer epigenetics and immune checkpoint therapy. Clin Cancer Res. 2023;29(7):1173–82. 10.1158/1078-0432.CCR-22-0784. 36449280 10.1158/1078-0432.CCR-22-0784 PMC10073242

[142] Meng H, Cao Y, Qin J, Song X, Zhang Q, Shi Y, et al. DNA methylation, its mediators and genome integrity. Int J Biol Sci. 2015;11(5):604–17. 10.7150/ijbs.11218. 25892967 10.7150/ijbs.11218 PMC4400391

[143] Lee AV, Nestler KA, Chiappinelli KB. Therapeutic targeting of DNA methylation alterations in cancer. Pharmacol Ther. 2024;258:108640. 10.1016/j.pharmthera.2024.108640. 38570075 10.1016/j.pharmthera.2024.108640

[144] Zeller C, Dai W, Steele NL, Siddiq A, Walley AJ, Wilhelm-Benartzi CS, et al. Candidate DNA methylation drivers of acquired cisplatin resistance in ovarian cancer identified by methylome and expression profiling. Oncogene. 2012;31(42):4567–76. 10.1038/onc.2011.611. 22249249 10.1038/onc.2011.611

[145] Zhao T, Bao Y, Gan X, Wang J, Chen Q, Dai Z, et al. DNA methylation-regulated QPCT promotes sunitinib resistance by increasing HRAS stability in renal cell carcinoma. Theranostics. 2019;9(21):6175–90. 10.7150/thno.35572. 31534544 10.7150/thno.35572 PMC6735520

[146] Liang J, Liao J, Chang R, Jia W, Li G, Chen Z, et al. Riplet promotes lipid metabolism changes associated with CD8 T cell exhaustion and anti-PD-1 resistance in hepatocellular carcinoma. Sci Immunol. 2025;10(108):eado3485. 10.1126/sciimmunol.ado3485. 40577442 10.1126/sciimmunol.ado3485

[147] Neganova ME, Klochkov SG, Aleksandrova YR, Aliev G. Histone modifications in epigenetic regulation of cancer: perspectives and achieved progress. Semin Cancer Biol. 2022;83:452. 10.1016/j.semcancer.2020.07.015. 32814115 10.1016/j.semcancer.2020.07.015

[148] Zhao A, Xu W, Han R, Wei J, Yu Q, Wang M, et al. Role of histone modifications in neurogenesis and neurodegenerative disease development. Ageing Res Rev. 2024;98:102324. 10.1016/j.arr.2024.102324. 38762100 10.1016/j.arr.2024.102324

[149] Baratchian M, Tiwari R, Khalighi S, Chakravarthy A, Yuan W, Berk M, et al. H3k9 methylation drives resistance to androgen receptor-antagonist therapy in prostate cancer. Proc Natl Acad Sci U S A. 2022;119(21):e2114324119. 10.1073/pnas.2114324119. 35584120 10.1073/pnas.2114324119 PMC9173765

[150] Chen Y, Wu S, Han Y, Shi H, Yuan J, Cui W. LncRNA SH3PXD2A-AS1 facilitates cisplatin resistance in non-small cell lung cancer by regulating FOXM1 succinylation. BMC Cancer. 2024;24(1):848. 10.1186/s12885-024-12624-9. 39020302 10.1186/s12885-024-12624-9 PMC11256434

[151] Wang Z, Wu H, Li Z, Chen Z, Feng A, Chu Y, et al. *PADI4* facilitates stem-like properties and cisplatin resistance through upregulating *PRMT2*/*IDs* family in oesophageal squamous cell carcinoma. Clin Transl Med. 2025;15(3):e70272. 10.1002/ctm2.70272. 40078091 10.1002/ctm2.70272 PMC11904308

[152] Chen S, Zhong M, Wang X, Su Y, Zhao Y, Wang S, et al. Activation of epigenetic reprogramming via crotonylation overcomes resistance to EGFR-TKI therapy in lung cancer. Proc Natl Acad Sci USA. 2025;122(40):e2509255122. 10.1073/pnas.2509255122. 41026825 10.1073/pnas.2509255122 PMC12519144

[153] Li W, Zhou C, Yu L, Hou Z, Liu H, Kong L, et al. Tumor-derived lactate promotes resistance to bevacizumab treatment by facilitating autophagy enhancer protein RUBCNL expression through histone H3 lysine 18 lactylation (H3K18la) in colorectal cancer. Autophagy. 2024;20(1):114–30. 10.1080/15548627.2023.2249762. 37615625 10.1080/15548627.2023.2249762 PMC10761097

[154] Yuan H, Wu X, Wu Q, Chatoff A, Megill E, Gao J, et al. Lysine catabolism reprograms tumour immunity through histone crotonylation. Nature. 2023;617(7962):818–26. 10.1038/s41586-023-06061-0. 37198486 10.1038/s41586-023-06061-0 PMC11089809

[155] Liu Z, Jin K, Xu Z, Xu J, Su X, Li B, et al. Gender disparities in clinical outcomes of urothelial carcinoma linked to X chromosome gene KDM6A mutation. BMJ Oncol. 2023;2(1):e000199. 10.1136/bmjonc-2023-000199. 39886491 10.1136/bmjonc-2023-000199 PMC11234999

[156] Eustermann S, Patel AB, Hopfner KP, He Y, Korber P. Energy-driven genome regulation by ATP-dependent chromatin remodellers. Nat Rev Mol Cell Biol. 2024;25(4):309–32. 10.1038/s41580-023-00683-y. 38081975 10.1038/s41580-023-00683-y PMC12303036

[157] Mashtalir N, Dao HT, Sankar A, Liu H, Corin AJ, Bagert JD, et al. Chromatin landscape signals differentially dictate the activities of mSWI/SNF family complexes. Science. 2021;373(6552):306–15. 10.1126/science.abf8705. 34437148 10.1126/science.abf8705 PMC8390793

[158] de Miguel FJ, Gentile C, Feng WW, Silva SJ, Sankar A, Exposito F, et al. Mammalian SWI/SNF chromatin remodeling complexes promote tyrosine kinase inhibitor resistance in EGFR-mutant lung cancer. Cancer Cell. 2023;41(8):1516-34 e9. 10.1016/j.ccell.2023.07.005. 37541244 10.1016/j.ccell.2023.07.005 PMC10957226

[159] Dayanc B, Eris S, Gulfirat NE, Ozden-Yilmaz G, Cakiroglu E, Coskun Deniz OS, et al. Integrative multi-omics identifies AP-1 transcription factor as a targetable mediator of acquired osimertinib resistance in non-small cell lung cancer. Cell Death Dis. 2025;16(1):414. 10.1038/s41419-025-07711-z. 40414926 10.1038/s41419-025-07711-z PMC12104440

[160] Liu Z, Zhang L, Jin K, Zeng H, Su X, Ding Y, et al. *ARID1A* loss plus CD8+ T-cell infiltration associate with favorable clinical outcomes in urothelial carcinoma. Clin Cancer Res. 2025;31(20):4311–22. 10.1158/1078-0432.CCR-25-0816. 40788182 10.1158/1078-0432.CCR-25-0816 PMC12521915

[161] Masliah-Planchon J, Bieche I, Guinebretiere JM, Bourdeaut F, Delattre O. SWI/SNF chromatin remodeling and human malignancies. Annu Rev Pathol. 2015;10(1):145. 10.1146/annurev-pathol-012414-040445. 25387058 10.1146/annurev-pathol-012414-040445

[162] Slack FJ, Chinnaiyan AM. The role of non-coding RNAs in oncology. Cell. 2019;179(5):1033–55. 10.1016/j.cell.2019.10.017. 31730848 10.1016/j.cell.2019.10.017 PMC7347159

[163] Matsui M, Corey DR. Non-coding RNAs as drug targets. Nat Rev Drug Discov. 2017;16(3):167–79. 10.1038/nrd.2016.117. 27444227 10.1038/nrd.2016.117 PMC5831170

[164] Huang H, Weng H, Chen J. M(6)A modification in coding and non-coding RNAs: roles and therapeutic implications in cancer. Cancer Cell. 2020;37(3):270–88. 10.1016/j.ccell.2020.02.004. 32183948 10.1016/j.ccell.2020.02.004 PMC7141420

[165] Wang H, Fleishman JS, Cheng S, Wang W, Wu F, Wang Y, et al. Epigenetic modification of ferroptosis by non-coding RNAs in cancer drug resistance. Mol Cancer. 2024;23(1):177. 10.1186/s12943-024-02088-7. 39192329 10.1186/s12943-024-02088-7 PMC11348582

[166] Han X, Li M, Xu J, Fu J, Wang X, Wang J, et al. MiR-1275 targets MDK/AKT signaling to inhibit breast cancer chemoresistance by lessening the properties of cancer stem cells. Int J Biol Sci. 2023;19(1):89–103. 10.7150/ijbs.74227. 36594100 10.7150/ijbs.74227 PMC9760432

[167] Qu L, Ding J, Chen C, Wu ZJ, Liu B, Gao Y, et al. Exosome-transmitted lncARSR promotes sunitinib resistance in renal cancer by acting as a competing endogenous RNA. Cancer Cell. 2016;29(5):653–68. 10.1016/j.ccell.2016.03.004. 27117758 10.1016/j.ccell.2016.03.004

[168] Hu Y, Cai ZR, Huang RZ, Wang DS, Ju HQ, Chen DL. Circular RNA circPHLPP2 promotes tumor growth and anti-PD-1 resistance through binding ILF3 to regulate IL36gamma transcription in colorectal cancer. Mol Cancer. 2024;23(1):272. 10.1186/s12943-024-02192-8. 39695693 10.1186/s12943-024-02192-8 PMC11658269

[169] Ou B, Liu Y, Gao Z, Xu J, Yan Y, Li Y, et al. Senescent neutrophils-derived exosomal piRNA-17560 promotes chemoresistance and EMT of breast cancer via FTO-mediated m6A demethylation. Cell Death Dis. 2022;13(10):905. 10.1038/s41419-022-05317-3. 36302751 10.1038/s41419-022-05317-3 PMC9613690

[170] Tan L, Mai D, Zhang B, Jiang X, Zhang J, Bai R, et al. PIWI-interacting RNA-36712 restrains breast cancer progression and chemoresistance by interaction with SEPW1 pseudogene SEPW1P RNA. Mol Cancer. 2019;18(1):9. 10.1186/s12943-019-0940-3. 30636640 10.1186/s12943-019-0940-3 PMC6330501

[171] Lu J, Zhu P, Zhang X, Zeng L, Xu B, Zhou P. tRNA-derived fragments: unveiling new roles and molecular mechanisms in cancer progression. Int J Cancer. 2024;155(8):1347–60. 10.1002/ijc.35041. 38867475 10.1002/ijc.35041

[172] Delaunay S, Helm M, Frye M. RNA modifications in physiology and disease: towards clinical applications. Nat Rev Genet. 2024;25(2):104–22. 10.1038/s41576-023-00645-2. 37714958 10.1038/s41576-023-00645-2

[173] Roundtree IA, Evans ME, Pan T, He C. Dynamic RNA modifications in gene expression regulation. Cell. 2017;169(7):1187–200. 10.1016/j.cell.2017.05.045. 28622506 10.1016/j.cell.2017.05.045 PMC5657247

[174] Barbieri I, Kouzarides T. Role of RNA modifications in cancer. Nat Rev Cancer. 2020;20(6):303–22. 10.1038/s41568-020-0253-2. 32300195 10.1038/s41568-020-0253-2

[175] Chen D, Gu X, Nurzat Y, Xu L, Li X, Wu L, et al. Writers, readers, and erasers RNA modifications and drug resistance in cancer. Mol Cancer. 2024;23(1):178. 10.1186/s12943-024-02089-6. 39215288 10.1186/s12943-024-02089-6 PMC11363509

[176] Zhang X, Xie K, Zhou H, Wu Y, Li C, Liu Y, et al. Role of non-coding RNAs and RNA modifiers in cancer therapy resistance. Mol Cancer. 2020;19(1):47. 10.1186/s12943-020-01171-z. 32122355 10.1186/s12943-020-01171-z PMC7050132

[177] Zhuang H, Yu B, Tao D, Xu X, Xu Y, Wang J, et al. The role of m6A methylation in therapy resistance in cancer. Mol Cancer. 2023;22(1):91. 10.1186/s12943-023-01782-2. 37264402 10.1186/s12943-023-01782-2 PMC10233906

[178] Xue H, Ma Y, Guan K, Zhou Y, Liu Y, Cao F, et al. The role of m6A methylation in targeted therapy resistance in lung cancer. Am J Cancer Res. 2024;14(6):2994–3009. 10.62347/LXOS2662. 39005690 10.62347/LXOS2662 PMC11236795

[179] Wang L, Yang Q, Zhou Q, Fang F, Lei K, Liu Z, et al. METTL3-m(6)A-EGFR-axis drives lenvatinib resistance in hepatocellular carcinoma. Cancer Lett. 2023;559:216122. 10.1016/j.canlet.2023.216122. 36898427 10.1016/j.canlet.2023.216122

[180] Wan W, Ao X, Chen Q, Yu Y, Ao L, Xing W, et al. METTL3/IGF2BP3 axis inhibits tumor immune surveillance by upregulating N(6)-methyladenosine modification of PD-L1 mRNA in breast cancer. Mol Cancer. 2022;21(1):60. 10.1186/s12943-021-01447-y. 35197058 10.1186/s12943-021-01447-y PMC8864846

[181] Nie S, Zhang L, Liu J, Wan Y, Jiang Y, Yang J, et al. ALKBH5-HOXA10 loop-mediated JAK2 m6a demethylation and cisplatin resistance in epithelial ovarian cancer. J Exp Clin Cancer Res. 2021;40(1):284. 10.1186/s13046-021-02088-1. 34496932 10.1186/s13046-021-02088-1 PMC8425158

[182] Tao EW, Wang Y, Tan J, Chen Y, Sun TY, Hao Y, et al. TRMT6-mediated tRNA m(1)a modification acts as a translational checkpoint of histone synthesis and facilitates colorectal cancer progression. Nat Cancer. 2025. 10.1038/s43018-025-00977-4. 40461825 10.1038/s43018-025-00977-4

[183] Liu Y, Zhang S, Gao X, Ru Y, Gu X, Hu X. Research progress of N1-methyladenosine RNA modification in cancer. Cell Commun Signal. 2024;22(1):79. 10.1186/s12964-023-01401-z. 38291517 10.1186/s12964-023-01401-z PMC10826226

[184] Huang M, Long J, Yao Z, Zhao Y, Zhao Y, Liao J, et al. METTL1-mediated m7G tRNA modification promotes lenvatinib resistance in hepatocellular carcinoma. Cancer Res. 2023;83(1):89–102. 10.1158/0008-5472.CAN-22-0963. 36102722 10.1158/0008-5472.CAN-22-0963

[185] Chen J, Li K, Chen J, Wang X, Ling R, Cheng M, et al. Aberrant translation regulated by METTL1/WDR4-mediated tRNA N7-methylguanosine modification drives head and neck squamous cell carcinoma progression. Cancer Commun. 2022;42(3):223–44. 10.1002/cac2.12273. 10.1002/cac2.12273 PMC8923133 35179319

[186] Bushweller JH. Targeting transcription factors in cancer — from undruggable to reality. Nat Rev Cancer. 2019;19(11):611–24. 10.1038/s41568-019-0196-7. 31511663 10.1038/s41568-019-0196-7 PMC8820243

[187] Donati G, Amati B. MYC and therapy resistance in cancer: risks and opportunities. Mol Oncol. 2022;16(21):3828–54. 10.1002/1878-0261.13319. 36214609 10.1002/1878-0261.13319 PMC9627787

[188] Tan XQ, Guo AY, Zheng LF, Xiong J. Research progress on FOXM1 in ovarian cancer diagnosis and therapeutics. Front Oncol. 2025;15:1598868. 10.3389/fonc.2025.1598868. 40612352 10.3389/fonc.2025.1598868 PMC12222324

[189] Zhao Y, Xing C, Deng Y, Ye C, Peng H. HIF-1alpha signaling: Essential roles in tumorigenesis and implications in targeted therapies. Genes Dis. 2024;11(1):234–51. 10.1016/j.gendis.2023.02.039. 37588219 10.1016/j.gendis.2023.02.039 PMC10425810

[190] Lee KM, Giltnane JM, Balko JM, Schwarz LJ, Guerrero-Zotano AL, Hutchinson KE, et al. MYC and MCL1 Cooperatively Promote Chemotherapy-Resistant Breast Cancer Stem Cells via Regulation of Mitochondrial Oxidative Phosphorylation. Cell Metab. 2017;26(4):633-47 e7. 10.1016/j.cmet.2017.09.009. 28978427 10.1016/j.cmet.2017.09.009 PMC5650077

[191] Li GH, Qu Q, Qi TT, Teng XQ, Zhu HH, Wang JJ, et al. Super-enhancers: a new frontier for epigenetic modifiers in cancer chemoresistance. J Exp Clin Cancer Res. 2021;40(1):174. 10.1186/s13046-021-01974-y. 34011395 10.1186/s13046-021-01974-y PMC8132395

[192] Sengupta S, George RE. Super-enhancer-driven transcriptional dependencies in cancer. Trends Cancer. 2017;3(4):269–81. 10.1016/j.trecan.2017.03.006. 28718439 10.1016/j.trecan.2017.03.006 PMC5546010

[193] Lu B, Chen S, Guan X, Chen X, Du Y, Yuan J, et al. Lactate accumulation induces H4K12la to activate super-enhancer-driven RAD23A expression and promote niraparib resistance in ovarian cancer. Mol Cancer. 2025;24(1):83. 10.1186/s12943-025-02295-w. 40102876 10.1186/s12943-025-02295-w PMC11921584

[194] Bi B, Qiu M, Liu P, Wang Q, Wen Y, Li Y, et al. Protein post-translational modifications: a key factor in colorectal cancer resistance mechanisms. Biochimica et Biophysica Acta (BBA). 2023;1866(4):194977. 10.1016/j.bbagrm.2023.194977. 10.1016/j.bbagrm.2023.194977 37625568

[195] Miao C, Huang Y, Zhang C, Wang X, Wang B, Zhou X, et al. Post-translational modifications in drug resistance. Drug Resist Updat. 2025;78:101173. 10.1016/j.drup.2024.101173. 39612546 10.1016/j.drup.2024.101173

[196] Yu L, Chen Z, Wu Y, Xu M, Zhong D, Xu H, et al. Unraveling role of ubiquitination in drug resistance of gynecological cancer. Am J Cancer Res. 2024;14(5):2523–37. 10.62347/WYKZ9784. 38859858 10.62347/WYKZ9784 PMC11162667

[197] Wang Z, Li Y, Mao R, Zhang Y, Wen J, Liu Q, et al. *DNAJB8* in small extracellular vesicles promotes Oxaliplatin resistance through *TP53/MDR1* pathway in colon cancer. Cell Death Dis. 2022;13(2):151. 10.1038/s41419-022-04599-x. 35165262 10.1038/s41419-022-04599-x PMC8844036

[198] Porcelli L, Giovannetti E, Assaraf YG, Jansen G, Scheffer GL, Kathman I, et al. The EGFR pathway regulates BCRP expression in NSCLC cells: role of erlotinib. Curr Drug Targets. 2014;15(14):1322–30. 10.2174/1389450116666141205145620. 25479544 10.2174/1389450116666141205145620

[199] Rehberger M, Schafer JA, Krampitz AM, Bretz AC, Jost L, Haferlach T, et al. The nuclear proteins TP73 and CUL4A confer resistance to cytarabine by induction of translesion DNA synthesis via mono-ubiquitination of PCNA. Hemasphere. 2022;6(5):e0708. 10.1097/HS9.0000000000000708. 35519003 10.1097/HS9.0000000000000708 PMC9067361

[200] Han F, Qi G, Li R, Peng J, Yan S, Yuan C, et al. USP28 promotes PARP inhibitor resistance by enhancing SOX9-mediated DNA damage repair in ovarian cancer. Cell Death Dis. 2025;16(1):305. 10.1038/s41419-025-07647-4. 40240356 10.1038/s41419-025-07647-4 PMC12003857

[201] Xiong W, Gao X, Zhang T, Jiang B, Hu MM, Bu X, et al. Usp8 inhibition reshapes an inflamed tumor microenvironment that potentiates the immunotherapy. Nat Commun. 2022;13(1):1700. 10.1038/s41467-022-29401-6. 35361799 10.1038/s41467-022-29401-6 PMC8971425

[202] Sun H, Zheng J, Xiao J, Yue J, Shi Z, Xuan Z, et al. TOPK/PBK is phosphorylated by ERK2 at serine 32, promotes tumorigenesis and is involved in sorafenib resistance in RCC. Cell Death Dis. 2022;13(5):450. 10.1038/s41419-022-04909-3. 35546143 10.1038/s41419-022-04909-3 PMC9095598

[203] Guo Y, He J, Zhang H, Chen R, Li L, Liu X, et al. Linear ubiquitination of PTEN impairs its function to promote prostate cancer progression. Oncogene. 2022;41(44):4877–92. 10.1038/s41388-022-02485-6. 36192478 10.1038/s41388-022-02485-6

[204] Xuan Z, Chen C, Sun H, Yang K, Li J, Fu M, et al. NDR1/FBXO11 promotes phosphorylation-mediated ubiquitination of beta-catenin to suppress metastasis in prostate cancer. Int J Biol Sci. 2024;20(12):4957–77. 10.7150/ijbs.98907. 39309441 10.7150/ijbs.98907 PMC11414387

[205] Chen Q, Yang B, Liu X, Zhang XD, Zhang L, Liu T. Histone acetyltransferases CBP/p300 in tumorigenesis and CBP/p300 inhibitors as promising novel anticancer agents. Theranostics. 2022;12(11):4935–48. 10.7150/thno.73223. 35836809 10.7150/thno.73223 PMC9274749

[206] Contreras-Sanzon E, Prado-Garcia H, Romero-Garcia S, Nunez-Corona D, Ortiz-Quintero B, Luna-Rivero C, et al. Histone deacetylases modulate resistance to the therapy in lung cancer. Front Genet. 2022;13:960263. 10.3389/fgene.2022.960263. 36263432 10.3389/fgene.2022.960263 PMC9574126

[207] Wei Z, Ye Y, Liu C, Wang Q, Zhang Y, Chen K, et al. MIER2/PGC1A elicits sunitinib resistance via lipid metabolism in renal cell carcinoma. J Adv Res. 2025;70:287–305. 10.1016/j.jare.2024.04.032. 38702028 10.1016/j.jare.2024.04.032 PMC11976417

[208] Sun Y, Wang H, Cui Z, Yu T, Song Y, Gao H, et al. Lactylation in cancer progression and drug resistance. Drug Resist Updat. 2025;81:101248. 10.1016/j.drup.2025.101248. 40287994 10.1016/j.drup.2025.101248

[209] Li F, Zhang H, Huang Y, Li D, Zheng Z, Xie K, et al. Single-cell transcriptome analysis reveals the association between histone lactylation and cisplatin resistance in bladder cancer. Drug Resist Updat. 2024;73:101059. 10.1016/j.drup.2024.101059. 38295753 10.1016/j.drup.2024.101059

[210] Yue Q, Wang Z, Shen Y, Lan Y, Zhong X, Luo X, et al. Histone H3K9 lactylation confers temozolomide resistance in glioblastoma via LUC7L2-mediated MLH1 intron retention. Adv Sci. 2024;11(19):e2309290. 10.1002/advs.202309290. 10.1002/advs.202309290 PMC11109612 38477507

[211] Li G, Wang D, Zhai Y, Pan C, Zhang J, Wang C, et al. Glycometabolic reprogramming-induced XRCC1 lactylation confers therapeutic resistance in ALDH1A3-overexpressing glioblastoma. Cell Metab. 2024;36(8):1696-710 e10. 10.1016/j.cmet.2024.07.011. 39111285 10.1016/j.cmet.2024.07.011

[212] Chang HM, Yeh ETH. SUMO: from bench to bedside. Physiol Rev. 2020;100(4):1599–619. 10.1152/physrev.00025.2019. 32666886 10.1152/physrev.00025.2019 PMC7717128

[213] Yuan H, Lu Y, Chan YT, Zhang C, Wang N, Feng Y. The Role of Protein SUMOylation in Human Hepatocellular Carcinoma: A Potential Target of New Drug Discovery and Development. Cancers (Basel). 2021;13(22). 10.3390/cancers13225700. 10.3390/cancers13225700 PMC8616375 34830854

[214] Feng D, He J, Yuan M, Chen Q, Zeng X, Zhou Q, et al. *SUMO2/3* promotes the progression and oxaliplatin resistance of colorectal cancer through facilitating the SUMOylation at Ku80-K307. BioFactors. 2023;49(6):1158–73. 10.1002/biof.1984. 37338025 10.1002/biof.1984

[215] Wei B, Yang F, Yu L, Qiu C. Crosstalk between SUMOylation and other post-translational modifications in breast cancer. Cell Mol Biol Lett. 2024;29(1):107. 10.1186/s11658-024-00624-3. 39127633 10.1186/s11658-024-00624-3 PMC11316377

[216] Garemilla SSS, Gampa SC, Garimella S. Role of the tumor microenvironment in cancer therapy: unveiling new targets to overcome drug resistance. Med Oncol. 2025;42(6):202. 10.1007/s12032-025-02754-w. 40332723 10.1007/s12032-025-02754-w

[217] Pu Y, Ji Q. Tumor-associated macrophages regulate PD-1/PD-L1 immunosuppression. Front Immunol. 2022;13:874589. 10.3389/fimmu.2022.874589. 35592338 10.3389/fimmu.2022.874589 PMC9110638

[218] Tie Y, Tang F, Wei YQ, Wei XW. Immunosuppressive cells in cancer: mechanisms and potential therapeutic targets. J Hematol Oncol. 2022;15(1):61. 10.1186/s13045-022-01282-8. 35585567 10.1186/s13045-022-01282-8 PMC9118588

[219] Rizzolio S, Giordano S, Corso S. The importance of being CAFs (in cancer resistance to targeted therapies). J Exp Clin Cancer Res. 2022;41(1):319. 10.1186/s13046-022-02524-w. 36324182 10.1186/s13046-022-02524-w PMC9632140

[220] Kundu M, Butti R, Panda VK, Malhotra D, Das S, Mitra T, et al. Modulation of the tumor microenvironment and mechanism of immunotherapy-based drug resistance in breast cancer. Mol Cancer. 2024;23(1):92. 10.1186/s12943-024-01990-4. 38715072 10.1186/s12943-024-01990-4 PMC11075356

[221] de Heer EC, Jalving M, Harris AL. HIFs, angiogenesis, and metabolism: elusive enemies in breast cancer. J Clin Invest. 2020;130(10):5074–87. 10.1172/JCI137552. 32870818 10.1172/JCI137552 PMC7524491

[222] Bao MH, Wong CC. Hypoxia, Metabolic Reprogramming, and Drug Resistance in Liver Cancer. Cells. 2021;10(7). 10.3390/cells10071715. 10.3390/cells10071715 PMC8304710 34359884

[223] Chang CH, Qiu J, O’Sullivan D, Buck MD, Noguchi T, Curtis JD, et al. Metabolic competition in the tumor microenvironment is a driver of cancer progression. Cell. 2015;162(6):1229–41. 10.1016/j.cell.2015.08.016. 26321679 10.1016/j.cell.2015.08.016 PMC4864363

[224] Mah AY, Rashidi A, Keppel MP, Saucier N, Moore EK, Alinger JB, et al. Glycolytic requirement for NK cell cytotoxicity and cytomegalovirus control. JCI Insight. 2017;2(23). 10.1172/jci.insight.95128. 10.1172/jci.insight.95128 PMC5752285 29212951

[225] Alabaster O, Woods T, Ortiz-Sanchez V, Jahangeer S. Influence of microenvironmental pH on adriamycin resistance. Cancer Res. 1989;49(20):5638–43. 2790781

[226] Qiu C, Tang C, Tang Y, Su K, Chai X, Zhan Z, et al. RGS5+ lymphatic endothelial cells facilitate metastasis and acquired drug resistance of breast cancer through oxidative stress-sensing mechanism. Drug Resist Updat. 2024;77:101149. 10.1016/j.drup.2024.101149. 39306871 10.1016/j.drup.2024.101149

[227] Ji S, Wu W, Jiang Q. Crosstalk between Endothelial Cells and Tumor Cells: A New Era in Prostate Cancer Progression. Int J Mol Sci. 2023;24(23). 10.3390/ijms242316893. 10.3390/ijms242316893 PMC10707594 38069225

[228] Lu Y, Liu Y, Zuo X, Li G, Wang J, Liu J, et al. CXCL12(+) tumor-associated endothelial cells promote immune resistance in hepatocellular carcinoma. J Hepatol. 2025;82(4):634–48. 10.1016/j.jhep.2024.09.044. 39393439 10.1016/j.jhep.2024.09.044

[229] Tan J, Fan W, Liu T, Zhu B, Liu Y, Wang S, et al. TREM2(+) macrophages suppress CD8(+) T-cell infiltration after transarterial chemoembolisation in hepatocellular carcinoma. J Hepatol. 2023;79(1):126–40. 10.1016/j.jhep.2023.02.032. 36889359 10.1016/j.jhep.2023.02.032

[230] Mai Z, Lin Y, Lin P, Zhao X, Cui L. Modulating extracellular matrix stiffness: a strategic approach to boost cancer immunotherapy. Cell Death Dis. 2024;15(5):307. 10.1038/s41419-024-06697-4. 38693104 10.1038/s41419-024-06697-4 PMC11063215

[231] Sleeboom JJF, van Tienderen GS, Schenke-Layland K, van der Laan LJW, Khalil AA, Verstegen MMA. The extracellular matrix as hallmark of cancer and metastasis: from biomechanics to therapeutic targets. Sci Transl Med. 2024;16(728):eadg3840. 10.1126/scitranslmed.adg3840. 38170791 10.1126/scitranslmed.adg3840

[232] Darvishi B, Eisavand MR, Majidzadeh AK, Farahmand L. Matrix stiffening and acquired resistance to chemotherapy: concepts and clinical significance. Br J Cancer. 2022;126(9):1253–63. 10.1038/s41416-021-01680-8. 35124704 10.1038/s41416-021-01680-8 PMC9043195

[233] Park CC, Zhang HJ, Yao ES, Park CJ, Bissell MJ. Beta1 integrin inhibition dramatically enhances radiotherapy efficacy in human breast cancer xenografts. Cancer Res. 2008;68(11):4398–405. 10.1158/0008-5472.CAN-07-6390. 18519702 10.1158/0008-5472.CAN-07-6390 PMC3719863

[234] Brown JA, Yonekubo Y, Hanson N, Sastre-Perona A, Basin A, Rytlewski JA, et al. TGF-beta-Induced Quiescence Mediates Chemoresistance of Tumor-Propagating Cells in Squamous Cell Carcinoma. Cell Stem Cell. 2017;21(5):650-64 e8. 10.1016/j.stem.2017.10.001. 29100014 10.1016/j.stem.2017.10.001 PMC5778452

[235] Zhong C, Tao B, Tang F, Yang X, Peng T, You J, et al. Remodeling cancer stemness by collagen/fibronectin via the AKT and CDC42 signaling pathway crosstalk in glioma. Theranostics. 2021;11(4):1991–2005. 10.7150/thno.50613. 33408794 10.7150/thno.50613 PMC7778591

[236] Chanmee T, Ontong P, Mochizuki N, Kongtawelert P, Konno K, Itano N. Excessive hyaluronan production promotes acquisition of cancer stem cell signatures through the coordinated regulation of Twist and the transforming growth factor beta (TGF-beta)-Snail signaling axis. J Biol Chem. 2014;289(38):26038–56. 10.1074/jbc.M114.564120. 25077968 10.1074/jbc.M114.564120 PMC4176217

[237] Lin CH, Pelissier FA, Zhang H, Lakins J, Weaver VM, Park C, et al. Microenvironment rigidity modulates responses to the HER2 receptor tyrosine kinase inhibitor lapatinib via YAP and TAZ transcription factors. Mol Biol Cell. 2015;26(22):3946–53. 10.1091/mbc.E15-07-0456. 26337386 10.1091/mbc.E15-07-0456 PMC4710228

[238] Long JE, Wongchenko MJ, Nickles D, Chung WJ, Wang BE, Riegler J, et al. Therapeutic resistance and susceptibility is shaped by cooperative multi-compartment tumor adaptation. Cell Death Differ. 2019;26(11):2416–29. 10.1038/s41418-019-0310-0. 30824837 10.1038/s41418-019-0310-0 PMC6889278

[239] Deng M, Lin J, Nowsheen S, Liu T, Zhao Y, Villalta PW, et al. Extracellular matrix stiffness determines DNA repair efficiency and cellular sensitivity to genotoxic agents. Sci Adv. 2020;6(37). 10.1126/sciadv.abb2630. 10.1126/sciadv.abb2630 PMC7486107 32917705

[240] Moreau JF, Pradeu T, Grignolio A, Nardini C, Castiglione F, Tieri P, et al. The emerging role of ECM crosslinking in T cell mobility as a hallmark of immunosenescence in humans. Ageing Res Rev. 2017;35:322. 10.1016/j.arr.2016.11.005. 27876574 10.1016/j.arr.2016.11.005

[241] Nicolas-Boluda A, Vaquero J, Vimeux L, Guilbert T, Barrin S, Kantari-Mimoun C, et al. Tumor stiffening reversion through collagen crosslinking inhibition improves T cell migration and anti-PD-1 treatment. Elife. 2021;10. 10.7554/eLife.58688. 10.7554/eLife.58688 PMC8203293 34106045

[242] Zhang T, Jia Y, Yu Y, Zhang B, Xu F, Guo H. Targeting the tumor biophysical microenvironment to reduce resistance to immunotherapy. Adv Drug Deliv Rev. 2022;186:114319. 10.1016/j.addr.2022.114319. 35545136 10.1016/j.addr.2022.114319

[243] Miyazawa A, Ito S, Asano S, Tanaka I, Sato M, Kondo M, et al. Regulation of PD-L1 expression by matrix stiffness in lung cancer cells. Biochem Biophys Res Commun. 2018;495(3):2344–9. 10.1016/j.bbrc.2017.12.115. 29274784 10.1016/j.bbrc.2017.12.115

[244] Eno MS, Brubaker JD, Campbell JE, De Savi C, Guzi TJ, Williams BD, et al. Discovery of BLU-945, a reversible, potent, and wild-type-sparing next-generation EGFR mutant inhibitor for treatment-resistant non-small-cell lung cancer. J Med Chem. 2022;65(14):9662–77. 10.1021/acs.jmedchem.2c00704. 35838760 10.1021/acs.jmedchem.2c00704 PMC9340769

[245] Paydas S. Management of adverse effects/toxicity of ibrutinib. Crit Rev Oncol Hematol. 2019;136:56–63. 10.1016/j.critrevonc.2019.02.001. 30878129 10.1016/j.critrevonc.2019.02.001

[246] Naeem A, Utro F, Wang Q, Cha J, Vihinen M, Martindale S, et al. Pirtobrutinib targets BTK C481S in ibrutinib-resistant CLL but second-site BTK mutations lead to resistance. Blood Adv. 2023;7(9):1929–43. 10.1182/bloodadvances.2022008447. 36287227 10.1182/bloodadvances.2022008447 PMC10202739

[247] Kim H, Xu H, George E, Hallberg D, Kumar S, Jagannathan V, et al. Combining PARP with ATR inhibition overcomes PARP inhibitor and platinum resistance in ovarian cancer models. Nat Commun. 2020;11(1):3726. 10.1038/s41467-020-17127-2. 32709856 10.1038/s41467-020-17127-2 PMC7381609

[248] Thatikonda V, Lyu H, Jurado S, Kostyrko K, Bristow CA, Albrecht C, et al. Co-targeting SOS1 enhances the antitumor effects of KRAS(G12C) inhibitors by addressing intrinsic and acquired resistance. Nat Cancer. 2024;5(9):1352–70. 10.1038/s43018-024-00800-6. 39103541 10.1038/s43018-024-00800-6 PMC11424490

[249] Li L, Nie L, Jordan A, Cai Q, Liu Y, Li Y, et al. Targeting glutaminase is therapeutically effective in ibrutinib-resistant mantle cell lymphoma. Haematologica. 2023;108(6):1616–27. 10.3324/haematol.2022.281538. 36420799 10.3324/haematol.2022.281538 PMC10230437

[250] Mukherjee N, Skees J, Todd KJ, West DA, Lambert KA, Robinson WA, et al. MCL1 inhibitors S63845/MIK665 plus Navitoclax synergistically kill difficult-to-treat melanoma cells. Cell Death Dis. 2020;11(6):443. 10.1038/s41419-020-2646-2. 32513939 10.1038/s41419-020-2646-2 PMC7280535

[251] Wong KM, Horton KJ, Coveler AL, Hingorani SR, Harris WP. Targeting the tumor stroma: the biology and clinical development of Pegylated Recombinant Human Hyaluronidase (PEGPH20). Curr Oncol Rep. 2017;19(7):47. 10.1007/s11912-017-0608-3. 28589527 10.1007/s11912-017-0608-3

[252] Morosi L, Meroni M, Ubezio P, Fuso Nerini I, Minoli L, Porcu L, et al. PEGylated recombinant human hyaluronidase (PEGPH20) pre-treatment improves intra-tumour distribution and efficacy of paclitaxel in preclinical models. J Exp Clin Cancer Res. 2021;40(1):286. 10.1186/s13046-021-02070-x. 34507591 10.1186/s13046-021-02070-x PMC8434701

[253] Heil F, Babitzki G, Julien-Laferriere A, Ooi CH, Hidalgo M, Massard C, et al. Vanucizumab mode of action: Serial biomarkers in plasma, tumor, and skin-wound-healing biopsies. Transl Oncol. 2021;14(2):100984. 10.1016/j.tranon.2020.100984. 33338877 10.1016/j.tranon.2020.100984 PMC7749407

[254] Hutchinson LG, Mueller HJ, Gaffney EA, Maini PK, Wagg J, Phipps A, et al. Modeling Longitudinal Preclinical Tumor Size Data to Identify Transient Dynamics in Tumor Response to Antiangiogenic Drugs. CPT Pharmacometrics Syst Pharmacol. 2016;5(11):636–45. 10.1002/psp4.12142. 27863175 10.1002/psp4.12142 PMC5192995

[255] Zhang H, Almuqbil RM, Alhudaithi SS, Sunbul FS, da Rocha SRP. Pulmonary administration of a CSF-1R inhibitor alters the balance of tumor-associated macrophages and supports first-line chemotherapy in a lung cancer model. Int J Pharm. 2021;598:120350. 10.1016/j.ijpharm.2021.120350. 33545279 10.1016/j.ijpharm.2021.120350

[256] Omstead AN, Paskewicz M, Gorbunova A, Zheng P, Salvitti MS, Mansoor R, et al. CSF-1R inhibitor, pexidartinib, sensitizes esophageal adenocarcinoma to PD-1 immune checkpoint blockade in a rat model. Carcinogenesis. 2022;43(9):842–50. 10.1093/carcin/bgac043. 35552655 10.1093/carcin/bgac043

[257] Wu SY, Jin X, Liu Y, Wang ZY, Zuo WJ, Ma D, et al. Mobilizing antigen-presenting mast cells in anti-PD-1-refractory triple-negative breast cancer: a phase 2 trial. Nat Med. 2025;31(7):2405–15. 10.1038/s41591-025-03776-7. 40563015 10.1038/s41591-025-03776-7

[258] Liu Z, Ji X, He D, Zhang R, Liu Q, Xin T. Nanoscale drug delivery systems in glioblastoma. Nanoscale Res Lett. 2022;17(1):27. 10.1186/s11671-022-03668-6. 35171358 10.1186/s11671-022-03668-6 PMC8850533

[259] Zhang Y, Ma H, Li L, Sun C, Yu C, Wang L, et al. Dual-Targeted Novel Temozolomide Nanocapsules Encapsulating siPKM2 Inhibit Aerobic Glycolysis to Sensitize Glioblastoma to Chemotherapy. Adv Mater. 2024;36(29):e2400502. 10.1002/adma.202400502. 38651254 10.1002/adma.202400502

[260] Liu B, Ji Q, Cheng Y, Liu M, Zhang B, Mei Q, et al. Biomimetic GBM-targeted drug delivery system boosting ferroptosis for immunotherapy of orthotopic drug-resistant GBM. J Nanobiotechnology. 2022;20(1):161. 10.1186/s12951-022-01360-6. 35351131 10.1186/s12951-022-01360-6 PMC8962245

[261] Qu C, Yuan H, Tian M, Zhang X, Xia P, Shi G, et al. Precise photodynamic therapy by Midkine nanobody-engineered nanoparticles remodels the microenvironment of pancreatic ductal adenocarcinoma and potentiates the immunotherapy. ACS Nano. 2024;18(5):4019–37. 10.1021/acsnano.3c07002. 38253029 10.1021/acsnano.3c07002

[262] Zhang G, Li N, Qi Y, Zhao Q, Zhan J, Yu D. Synergistic ferroptosis-gemcitabine chemotherapy of the gemcitabine loaded carbonaceous nanozymes to enhance the treatment and magnetic resonance imaging monitoring of pancreatic cancer. Acta Biomater. 2022;142:284. 10.1016/j.actbio.2022.02.006. 35151925 10.1016/j.actbio.2022.02.006

[263] Jones JJ, Jones KL, Wong SQ, Whittle J, Goode D, Nguyen H, et al. Plasma ctDNA enables early detection of temozolomide resistance mutations in glioma. Neuro-Oncol Adv. 2024;6(1):vdae041. 10.1093/noajnl/vdae041. 10.1093/noajnl/vdae041 PMC11003533 38596716

[264] Malla M, Loree JM, Kasi PM, Parikh AR. Using circulating tumor DNA in colorectal cancer: current and evolving practices. J Clin Oncol. 2022;40(24):2846–57. 10.1200/JCO.21.02615. 35839443 10.1200/JCO.21.02615 PMC9390824

[265] Di Martino MT, Meschini S, Scotlandi K, Riganti C, De Smaele E, Zazzeroni F, et al. From single gene analysis to single cell profiling: a new era for precision medicine. J Exp Clin Cancer Res. 2020;39(1):48. 10.1186/s13046-020-01549-3. 32138788 10.1186/s13046-020-01549-3 PMC7059661

[266] Dufva O, Gandolfi S, Huuhtanen J, Dashevsky O, Duan H, Saeed K, et al. Single-cell functional genomics reveals determinants of sensitivity and resistance to natural killer cells in blood cancers. Immunity. 2023;56(12):2816 e13-2835. 10.1016/j.immuni.2023.11.008. 38091953 10.1016/j.immuni.2023.11.008

[267] Kashima Y, Shibahara D, Suzuki A, Muto K, Kobayashi IS, Plotnick D, et al. Single-Cell Analyses Reveal Diverse Mechanisms of Resistance to EGFR Tyrosine Kinase Inhibitors in Lung Cancer. Cancer Res. 2021;81(18):4835–48. 10.1158/0008-5472.CAN-20-2811. 34247147 10.1158/0008-5472.CAN-20-2811 PMC8448980

[268] Zhang S, Yuan L, Danilova L, Mo G, Zhu Q, Deshpande A, et al. Spatial transcriptomics analysis of neoadjuvant cabozantinib and nivolumab in advanced hepatocellular carcinoma identifies independent mechanisms of resistance and recurrence. Genome Med. 2023;15(1):72. 10.1186/s13073-023-01218-y. 37723590 10.1186/s13073-023-01218-y PMC10506285

[269] Bueschbell B, Caniceiro AB, Suzano PMS, Machuqueiro M, Rosario-Ferreira N, Moreira IS. Network biology and artificial intelligence drive the understanding of the multidrug resistance phenotype in cancer. Drug Resist Updat. 2022;60:100811. 10.1016/j.drup.2022.100811. 35121338 10.1016/j.drup.2022.100811

[270] Li W, Huang YH, Zhu T, Zhang YM, Zheng XX, Zhang TF, et al. Noninvasive Artificial Intelligence System for Early Predicting Residual Cancer Burden During Neoadjuvant Chemotherapy in Breast Cancer. Ann Surg. 2025;281(4):645–54. 10.1097/SLA.0000000000006279. 38557792 10.1097/SLA.0000000000006279 PMC11888841

[271] Zhang Z, Wang ZX, Chen YX, Wu HX, Yin L, Zhao Q, et al. Integrated analysis of single-cell and bulk RNA sequencing data reveals a pan-cancer stemness signature predicting immunotherapy response. Genome Med. 2022;14(1):45. 10.1186/s13073-022-01050-w. 35488273 10.1186/s13073-022-01050-w PMC9052621

[272] Wang S, Yu H, Gan Y, Wu Z, Li E, Li X, et al. Mining whole-lung information by artificial intelligence for predicting EGFR genotype and targeted therapy response in lung cancer: a multicohort study. Lancet Digit Health. 2022;4(5):e309–19. 10.1016/S2589-7500(22)00024-3. 35341713 10.1016/S2589-7500(22)00024-3

[273] Qi Z, Xu Z, Zhang L, Zou Y, Li J, Yan W, et al. Overcoming resistance to immune checkpoint therapy in PTEN-null prostate cancer by intermittent anti-PI3Kalpha/beta/delta treatment. Nat Commun. 2022;13(1):182. 10.1038/s41467-021-27833-0. 35013322 10.1038/s41467-021-27833-0 PMC8748754

[274] Ruiz-Vico M, Wetterskog D, Orlando F, Thakali S, Wingate A, Jayaram A, et al. Liquid biopsy in progressing prostate cancer patients starting docetaxel with or without enzalutamide: a biomarker study of the PRESIDE phase 3b trial. Eur Urol Oncol. 2025;8(1):135–44. 10.1016/j.euo.2024.08.006. 39261236 10.1016/j.euo.2024.08.006

[275] Fidelle M, Rauber C, Alves Costa Silva C, Tian AL, Lahmar I, de La Varende AM, et al. A microbiota-modulated checkpoint directs immunosuppressive intestinal T cells into cancers. Science. 2023;380(6649):eabo2296. 10.1126/science.abo2296. 37289890 10.1126/science.abo2296

[276] Joachim L, Gottert S, Sax A, Steiger K, Neuhaus K, Heinrich P, et al. The microbial metabolite desaminotyrosine enhances T-cell priming and cancer immunotherapy with immune checkpoint inhibitors. EBioMedicine. 2023;97:104834. 10.1016/j.ebiom.2023.104834. 37865045 10.1016/j.ebiom.2023.104834 PMC10597767

[277] Huang J, Liu D, Wang Y, Liu L, Li J, Yuan J, et al. Ginseng polysaccharides alter the gut microbiota and kynurenine/tryptophan ratio, potentiating the antitumour effect of antiprogrammed cell death 1/programmed cell death ligand 1 (anti-PD-1/PD-L1) immunotherapy. Gut. 2022;71(4):734–45. 10.1136/gutjnl-2020-321031. 34006584 10.1136/gutjnl-2020-321031 PMC8921579

[278] Rodriguez-Garcia A, Ancos-Pintado R, Garcia-Vicente R, Ortiz-Ruiz A, Arroyo A, Navarro MA, et al. Microbiota-derived urolithin A in monoclonal gammopathies and multiple myeloma therapy. Microbiome. 2025;13(1):56. 10.1186/s40168-025-02045-6. 40022244 10.1186/s40168-025-02045-6 PMC11869585

[279] Wu H, Liu J, Zhang XH, Jin S, Li P, Liu H, et al. The combination of flaxseed lignans and PD-1/ PD-L1 inhibitor inhibits breast cancer growth via modulating gut microbiome and host immunity. Drug Resist Updat. 2025;80:101222. 10.1016/j.drup.2025.101222. 40048957 10.1016/j.drup.2025.101222

[280] Xu Q, Gao J, Zhao R, Li H, Cui H, Yuan Z, et al. *Akkermansia muciniphila*-derived pentadecanoic acid enhances oxaliplatin sensitivity in gastric cancer by modulating glycolysis. Pharmacol Res. 2024;206:107278. 10.1016/j.phrs.2024.107278. 38908613 10.1016/j.phrs.2024.107278

[281] Kim CY, Park DJ, Ahn BC, Baek S, Hong MH, Nguyen LT, et al. A conserved pilin from uncultured gut bacterial clade TANB77 enhances cancer immunotherapy. Nat Commun. 2024;15(1):10726. 10.1038/s41467-024-55388-3. 39730328 10.1038/s41467-024-55388-3 PMC11680825

[282] Mannion J, Gifford V, Bellenie B, Fernando W, Ramos Garcia L, Wilson R, et al. A RIPK1-specific PROTAC degrader achieves potent antitumor activity by enhancing immunogenic cell death. Immunity. 2024;57(7):1514-32 e15. 10.1016/j.immuni.2024.04.025. 38788712 10.1016/j.immuni.2024.04.025 PMC11236506

[283] Schroder M, Renatus M, Liang X, Meili F, Zoller T, Ferrand S, et al. DCAF1-based PROTACs with activity against clinically validated targets overcoming intrinsic- and acquired-degrader resistance. Nat Commun. 2024;15(1):275. 10.1038/s41467-023-44237-4. 38177131 10.1038/s41467-023-44237-4 PMC10766610

[284] Gough SM, Flanagan JJ, Teh J, Andreoli M, Rousseau E, Pannone M, et al. Oral estrogen receptor PROTAC vepdegestrant (ARV-471) is highly efficacious as monotherapy and in combination with CDK4/6 or PI3K/mTOR pathway inhibitors in preclinical ER+ breast cancer models. Clin Cancer Res. 2024;30(16):3549–63. 10.1158/1078-0432.CCR-23-3465. 38819400 10.1158/1078-0432.CCR-23-3465 PMC11325148

[285] Yu X, Lu D, Qi X, Paudel RR, Lin H, Holloman BL, et al. Development of a RIPK1 degrader to enhance antitumor immunity. Nat Commun. 2024;15(1):10683. 10.1038/s41467-024-55006-2. 39681571 10.1038/s41467-024-55006-2 PMC11649918

[286] Guo Y, Li Y, Zhou Z, Hou L, Liu W, Ren W, et al. Targeting PRMT5 through PROTAC for the treatment of triple-negative breast cancer. J Exp Clin Cancer Res. 2024;43(1):314. 10.1186/s13046-024-03237-y. 39614393 10.1186/s13046-024-03237-y PMC11607928

[287] Zhao LP, Zheng RR, Rao XN, Huang CY, Zhou HY, Yu XY, et al. Chemotherapy-Enabled Colorectal Cancer Immunotherapy of Self-Delivery Nano-PROTACs by Inhibiting Tumor Glycolysis and Avoiding Adaptive Immune Resistance. Adv Sci (Weinh). 2024;11(15):e2309204. 10.1002/advs.202309204. 38239040 10.1002/advs.202309204 PMC11022706

[288] Fu X, Huang J, Chen X, Xie D, Chen H, Liang Z, et al. Development of dual aptamers-functionalized c-MET PROTAC degraders for targeted therapy of osteosarcoma. Theranostics. 2025;15(1):103–21. 10.7150/thno.99588. 39744222 10.7150/thno.99588 PMC11667235

[289] Vartak R, Patel K. Targeted nanoliposomes of oncogenic protein degraders: significant inhibition of tumor in lung-cancer bearing mice. J Control Release. 2024;376:502. 10.1016/j.jconrel.2024.10.007. 39406280 10.1016/j.jconrel.2024.10.007

[290] Chia S, Wen Seow JJ, Peras da Silva R, Suphavilai C, Shirgaonkar N, Murata-Hori M, et al. CAN-Scan: A multi-omic phenotype-driven precision oncology platform identifies prognostic biomarkers of therapy response for colorectal cancer. Cell Rep Med. 2025;6(4):102053. 10.1016/j.xcrm.2025.102053. 40187357 10.1016/j.xcrm.2025.102053 PMC12047494

[291] Chen D, Liu P, Lin J, Zang L, Liu Y, Zhai S, et al. A Distinguished Roadmap of Fibroblast Senescence in Predicting Immunotherapy Response and Prognosis Across Human Cancers. Adv Sci (Weinh). 2025;12(7):e2406624. 10.1002/advs.202406624. 39739618 10.1002/advs.202406624 PMC11831569

[292] Fomin V, So WV, Barbieri RA, Hiller-Bittrolff K, Koletou E, Tu T, et al. Machine learning identifies clinical tumor mutation landscape pathways of resistance to checkpoint inhibitor therapy in NSCLC. J Immunother Cancer. 2025;13(3). 10.1136/jitc-2024-009092. 10.1136/jitc-2024-009092 PMC11877243 40032600

[293] Ricciuti B, Lamberti G, Puchala SR, Mahadevan NR, Lin JR, Alessi JV, et al. Genomic and immunophenotypic landscape of acquired resistance to PD-(L)1 blockade in non-small-cell lung cancer. J Clin Oncol. 2024;42(11):1311–21. 10.1200/JCO.23.00580. 38207230 10.1200/JCO.23.00580 PMC11095860

[294] Sahni S, Wang B, Wu D, Dhruba SR, Nagy M, Patkar S, et al. A machine learning model reveals expansive downregulation of ligand-receptor interactions that enhance lymphocyte infiltration in melanoma with developed resistance to immune checkpoint blockade. Nat Commun. 2024;15(1):8867. 10.1038/s41467-024-52555-4. 39402030 10.1038/s41467-024-52555-4 PMC11473774

[295] Hamidi H, Senbabaoglu Y, Beig N, Roels J, Manuel C, Guan X, et al. Molecular heterogeneity in urothelial carcinoma and determinants of clinical benefit to PD-L1 blockade. Cancer Cell. 2024;42(12):2098-112 e4. 10.1016/j.ccell.2024.10.016. 39577421 10.1016/j.ccell.2024.10.016

[296] Zhou Y, Liu Z, Gong C, Zhang J, Zhao J, Zhang X, et al. Targeting treatment resistance: unveiling the potential of RNA methylation regulators and TG-101,209 in pan-cancer neoadjuvant therapy. J Exp Clin Cancer Res. 2024;43(1):232. 10.1186/s13046-024-03111-x. 39160604 10.1186/s13046-024-03111-x PMC11331809

[297] Chen Y, He L, Ianevski A, Nader K, Ruokoranta T, Linnavirta N, et al. A machine learning-based strategy predicts selective and synergistic drug combinations for relapsed acute myeloid leukemia. Cancer Res. 2025;85(14):2753–68. 10.1158/0008-5472.CAN-24-3840. 40354625 10.1158/0008-5472.CAN-24-3840 PMC12260508

[298] Zhuo Z, Wang J, Zhang Y, Meng G. Integrative alternative splicing analysis reveals new prognosis signature in B-cell acute lymphoblastic leukemia. Int J Biol Sci. 2024;20(11):4496–512. 10.7150/ijbs.98899. 39247833 10.7150/ijbs.98899 PMC11380455

[299] Ianevski A, Nader K, Driva K, Senkowski W, Bulanova D, Moyano-Galceran L, et al. Single-cell transcriptomes identify patient-tailored therapies for selective co-inhibition of cancer clones. Nat Commun. 2024;15(1):8579. 10.1038/s41467-024-52980-5. 39362905 10.1038/s41467-024-52980-5 PMC11450203

[300] Sun W, Zhu Y, Zou Z, Wang L, Zhong J, Shen K, et al. An advanced comprehensive muti-cell-type-specific model for predicting anti-PD-1 therapeutic effect in melanoma. Theranostics. 2024;14(5):2127–50. 10.7150/thno.91626. 38505619 10.7150/thno.91626 PMC10945348

[301] Tamura T, Nagai S, Masuda K, Imaeda K, Sugihara E, Yamasaki J, et al. mTOR-mediated p62/SQSTM1 stabilization confers a robust survival mechanism for ovarian cancer. Cancer Lett. 2025;616:217565. 10.1016/j.canlet.2025.217565. 39971122 10.1016/j.canlet.2025.217565

[302] Sun J, Zhu Z, Li W, Shen M, Cao C, Sun Q, et al. UBE2T-regulated H2AX monoubiquitination induces hepatocellular carcinoma radioresistance by facilitating CHK1 activation. J Exp Clin Cancer Res. 2020;39(1):222. 10.1186/s13046-020-01734-4. 33087136 10.1186/s13046-020-01734-4 PMC7576867

[303] Hong W, Zhang Y, Wang S, Zheng D, Hsu S, Zhou J, et al. Deciphering the immune modulation through deep transcriptomic profiling and therapeutic implications of DNA damage repair pattern in hepatocellular carcinoma. Cancer Lett. 2024;582:216594. 10.1016/j.canlet.2023.216594. 38135208 10.1016/j.canlet.2023.216594

[304] Tang T, Zhou Z, Chen M, Li N, Sun J, Chen Z, et al. Plasma metabolic profiles-based prediction of induction chemotherapy efficacy in nasopharyngeal carcinoma: results of a bidirectional clinical trial. Clin Cancer Res. 2024;30(14):2925–36. 10.1158/1078-0432.CCR-23-3608. 38713248 10.1158/1078-0432.CCR-23-3608 PMC11247322

[305] Zhao X, Singhal A, Park S, Kong J, Bachelder R, Ideker T. Cancer mutations converge on a collection of protein assemblies to predict resistance to replication stress. Cancer Discov. 2024;14(3):508–23. 10.1158/2159-8290.CD-23-0641. 38236062 10.1158/2159-8290.CD-23-0641 PMC10905674

[306] Joshi SS, Badgwell BD. Current treatment and recent progress in gastric cancer. CA Cancer J Clin. 2021;71(3):264–79. 10.3322/caac.21657. 33592120 10.3322/caac.21657 PMC9927927

[307] Chen Z, Chen Y, Sun Y, Tang L, Zhang L, Hu Y, et al. Predicting gastric cancer response to anti-HER2 therapy or anti-HER2 combined immunotherapy based on multi-modal data. Signal Transduct Target Ther. 2024;9(1):222. 10.1038/s41392-024-01932-y. 39183247 10.1038/s41392-024-01932-y PMC11345439

[308] Chen J, Wang X, Ma A, Wang QE, Liu B, Li L, et al. Deep transfer learning of cancer drug responses by integrating bulk and single-cell RNA-seq data. Nat Commun. 2022;13(1):6494. 10.1038/s41467-022-34277-7. 36310235 10.1038/s41467-022-34277-7 PMC9618578

[309] Bi X, Wang J, Xue B, He C, Liu F, Chen H, et al. Sersomes for metabolic phenotyping and prostate cancer diagnosis. Cell Rep Med. 2024;5(6):101579. 10.1016/j.xcrm.2024.101579. 38776910 10.1016/j.xcrm.2024.101579 PMC11228451

[310] Castro E, Goh C, Olmos D, Saunders E, Leongamornlert D, Tymrakiewicz M, et al. Germline BRCA mutations are associated with higher risk of nodal involvement, distant metastasis, and poor survival outcomes in prostate cancer. J Clin Oncol. 2013;31(14):1748–57. 10.1200/JCO.2012.43.1882. 23569316 10.1200/JCO.2012.43.1882 PMC3641696

[311] Elmarakeby HA, Hwang J, Arafeh R, Crowdis J, Gang S, Liu D, et al. Biologically informed deep neural network for prostate cancer discovery. Nature. 2021;598(7880):348–52. 10.1038/s41586-021-03922-4. 34552244 10.1038/s41586-021-03922-4 PMC8514339

[312] Gallagher K, Strobl MAR, Park DS, Spoendlin FC, Gatenby RA, Maini PK, et al. Mathematical model-driven deep learning enables personalized adaptive therapy. Cancer Res. 2024;84(11):1929–41. 10.1158/0008-5472.CAN-23-2040. 38569183 10.1158/0008-5472.CAN-23-2040 PMC11148552

[313] Sonke GS, van Ommen-Nijhof A, Wortelboer N, van der Noort V, Swinkels ACP, Blommestein HM, et al. Early versus deferred use of CDK4/6 inhibitors in advanced breast cancer. Nature. 2024;636(8042):474–80. 10.1038/s41586-024-08035-2. 39604725 10.1038/s41586-024-08035-2

[314] Kalinsky K, Bianchini G, Hamilton E, Graff SL, Park KH, Jeselsohn R, et al. Abemaciclib plus fulvestrant in advanced breast cancer after progression on CDK4/6 inhibition: results from the phase III postMONARCH trial. J Clin Oncol. 2025;43(9):1101–12. 10.1200/JCO-24-02086. 39693591 10.1200/JCO-24-02086 PMC11936477

[315] Park S, Silva E, Singhal A, Kelly MR, Licon K, Panagiotou I, et al. A deep learning model of tumor cell architecture elucidates response and resistance to CDK4/6 inhibitors. Nat Cancer. 2024;5(7):996–1009. 10.1038/s43018-024-00740-1. 38443662 10.1038/s43018-024-00740-1 PMC11286358

[316] Swain SM, Shastry M, Hamilton E. Targeting HER2-positive breast cancer: advances and future directions. Nat Rev Drug Discov. 2023;22(2):101–26. 10.1038/s41573-022-00579-0. 36344672 10.1038/s41573-022-00579-0 PMC9640784

[317] Cameron D, Piccart-Gebhart MJ, Gelber RD, Procter M, Goldhirsch A, de Azambuja E, et al. 11 years’ follow-up of trastuzumab after adjuvant chemotherapy in HER2-positive early breast cancer: final analysis of the HERceptin Adjuvant (HERA) trial. Lancet. 2017;389(10075):1195–205. 10.1016/S0140-6736(16)32616-2. 28215665 10.1016/S0140-6736(16)32616-2 PMC5465633

[318] Kim J, Son HY, Lee S, Rho HW, Kim R, Jeong H, et al. Deep learning-assisted monitoring of trastuzumab efficacy in HER2-overexpressing breast cancer via SERS immunoassays of tumor-derived urinary exosomal biomarkers. Biosens Bioelectron. 2024;258:116347. 10.1016/j.bios.2024.116347. 38723332 10.1016/j.bios.2024.116347

[319] Castellanos E, Feld E, Horn L. Driven by mutations: the predictive value of mutation subtype in EGFR-mutated non-small cell lung cancer. J Thorac Oncol. 2017;12(4):612–23. 10.1016/j.jtho.2016.12.014. 28017789 10.1016/j.jtho.2016.12.014

[320] Wu YL, Zhou Q. Combination therapy for EGFR-mutated lung cancer. N Engl J Med. 2023;389(21):2005–7. 10.1056/NEJMe2311559. 37937797 10.1056/NEJMe2311559

[321] Jin W, Zhang Y, Zhao Z, Gao M. Developing targeted therapies for neuroblastoma by dissecting the effects of metabolic reprogramming on tumor microenvironments and progression. Theranostics. 2024;14(9):3439–69. 10.7150/thno.93962. 38948053 10.7150/thno.93962 PMC11209723

[322] Shadman M. Diagnosis and treatment of chronic lymphocytic leukemia: a review. JAMA. 2023;329(11):918–32. 10.1001/jama.2023.1946. 36943212 10.1001/jama.2023.1946

[323] Hallek M, Shanafelt TD, Eichhorst B. Chronic lymphocytic leukaemia. Lancet. 2018;391(10129):1524–37. 10.1016/S0140-6736(18)30422-7. 29477250 10.1016/S0140-6736(18)30422-7

[324] Shah K, Nasimian A, Ahmed M, Al Ashiri L, Denison L, Sime W, et al. PLK1 as a cooperating partner for BCL2-mediated antiapoptotic program in leukemia. Blood Cancer J. 2023;13(1):139. 10.1038/s41408-023-00914-7. 37679323 10.1038/s41408-023-00914-7 PMC10484999

[325] Johannet P, Coudray N, Donnelly DM, Jour G, Illa-Bochaca I, Xia Y, et al. Using machine learning algorithms to predict immunotherapy response in patients with advanced melanoma. Clin Cancer Res. 2021;27(1):131–40. 10.1158/1078-0432.CCR-20-2415. 33208341 10.1158/1078-0432.CCR-20-2415 PMC7785656

[326] Cai Z, Apolinario S, Baiao AR, Pacini C, Sousa MD, Vinga S, et al. Synthetic augmentation of cancer cell line multi-omic datasets using unsupervised deep learning. Nat Commun. 2024;15(1):10390. 10.1038/s41467-024-54771-4. 39614072 10.1038/s41467-024-54771-4 PMC11607321

[327] Baran N, Konopleva M. Molecular pathways: Hypoxia-activated prodrugs in cancer therapy. Clin Cancer Res. 2017;23(10):2382–90. 10.1158/1078-0432.CCR-16-0895. 28137923 10.1158/1078-0432.CCR-16-0895 PMC5433896

[328] Tap WD, Papai Z, Van Tine BA, Attia S, Ganjoo KN, Jones RL, et al. Doxorubicin plus evofosfamide versus doxorubicin alone in locally advanced, unresectable or metastatic soft-tissue sarcoma (TH CR-406/SARC021): an international, multicentre, open-label, randomised phase 3 trial. Lancet Oncol. 2017;18(8):1089–103. 10.1016/S1470-2045(17)30381-9. 28651927 10.1016/S1470-2045(17)30381-9 PMC7771354

[329] Zhang P, Wang X, Cen X, Zhang Q, Fu Y, Mei Y, et al. A deep learning framework for in silico screening of anticancer drugs at the single-cell level. Natl Sci Rev. 2025;12(2):nwae451. 10.1093/nsr/nwae451. 39872221 10.1093/nsr/nwae451 PMC11771446

[330] Ye Z, Chen F, Zeng J, Gao J, Zhang MQ. Scaffcomb: a phenotype-based framework for drug combination virtual screening in large-scale chemical datasets. Adv Sci. 2021;8(24):e2102092. 10.1002/advs.202102092. 10.1002/advs.202102092 PMC8693048 34723439

[331] Lin S, Tu C, Hu R, Wang H, Dong Z, Luo H, et al. Dr. Kinase: predicting the drug-resistance hotspots of protein kinases. Nucleic Acids Res. 2025;53(W1):W258–65. 10.1093/nar/gkaf366. 40308214 10.1093/nar/gkaf366 PMC12230729

[332] Friedmann Angeli JP, Krysko DV, Conrad M. Ferroptosis at the crossroads of cancer-acquired drug resistance and immune evasion. Nat Rev Cancer. 2019;19(7):405–14. 10.1038/s41568-019-0149-1. 31101865 10.1038/s41568-019-0149-1

[333] Vasan N, Baselga J, Hyman DM. A view on drug resistance in cancer. Nature. 2019;575(7782):299–309. 10.1038/s41586-019-1730-1. 31723286 10.1038/s41586-019-1730-1 PMC8008476

[334] Morad G, Helmink BA, Sharma P, Wargo JA. Hallmarks of response, resistance, and toxicity to immune checkpoint blockade. Cell. 2021;184(21):5309–37. 10.1016/j.cell.2021.09.020. 34624224 10.1016/j.cell.2021.09.020 PMC8767569

[335] Honkala A, Malhotra SV, Kummar S, Junttila MR. Harnessing the predictive power of preclinical models for oncology drug development. Nat Rev Drug Discov. 2022;21(2):99–114. 10.1038/s41573-021-00301-6. 34702990 10.1038/s41573-021-00301-6

[336] Michaelis M, Wass MN, Cinatl J. Drug-adapted cancer cell lines as preclinical models of acquired resistance. Cancer Drug Resist. 2019;2(3):447–56. 10.20517/cdr.2019.005. 35582596 10.20517/cdr.2019.005 PMC8992517

[337] Adriani G, Cappello P, Lovisa S. Editorial: Preclinical models and emerging technologies to study the effects of the tumor microenvironment on cancer heterogeneity and drug resistance. Front Oncol. 2023;13:1289756. 10.3389/fonc.2023.1289756. 37841424 10.3389/fonc.2023.1289756 PMC10569458

[338] LeBeau AM, Sevillano N, King ML, Duriseti S, Murphy ST, Craik CS, et al. Imaging the urokinase plasminongen activator receptor in preclinical breast cancer models of acquired drug resistance. Theranostics. 2014;4(3):267–79. 10.7150/thno.7323. 24505235 10.7150/thno.7323 PMC3915090

[339] Alkema NG, Wisman GB, van der Zee AG, van Vugt MA, de Jong S. Studying platinum sensitivity and resistance in high-grade serous ovarian cancer: Different models for different questions. Drug Resist Updat. 2016;24:55–69. 10.1016/j.drup.2015.11.005. 26830315 10.1016/j.drup.2015.11.005

[340] Vlachogiannis G, Hedayat S, Vatsiou A, Jamin Y, Fernandez-Mateos J, Khan K, et al. Patient-derived organoids model treatment response of metastatic gastrointestinal cancers. Science. 2018;359(6378):920–6. 10.1126/science.aao2774. 29472484 10.1126/science.aao2774 PMC6112415

[341] Van Allen EM, Miao D, Schilling B, Shukla SA, Blank C, Zimmer L, et al. Genomic correlates of response to CTLA-4 blockade in metastatic melanoma. Science. 2015;350(6257):207–11. 10.1126/science.aad0095. 26359337 10.1126/science.aad0095 PMC5054517

[342] Xin L, Xiao W, Che L, Liu J, Miccio L, Bianco V, et al. Label-free assessment of the drug resistance of epithelial ovarian cancer cells in a microfluidic holographic flow cytometer boosted through machine learning. ACS Omega. 2021;6(46):31046–57. 10.1021/acsomega.1c04204. 34841147 10.1021/acsomega.1c04204 PMC8613806

[343] Lambo S, Trinh DL, Ries RE, Jin D, Setiadi A, Ng M, et al. A longitudinal single-cell atlas of treatment response in pediatric AML. Cancer Cell. 2023;41(12):2117-35 e12. 10.1016/j.ccell.2023.10.008. 37977148 10.1016/j.ccell.2023.10.008

[344] Zhang D, Deng Y, Kukanja P, Agirre E, Bartosovic M, Dong M, et al. Spatial epigenome-transcriptome co-profiling of mammalian tissues. Nature. 2023;616(7955):113–22. 10.1038/s41586-023-05795-1. 36922587 10.1038/s41586-023-05795-1 PMC10076218

[345] Han X, Ma Q, Chang R, Xin S, Zhang G, Wang R, et al. Identification of m(6)A-modified gene signatures in lung adenocarcinoma tumorigenesis and their potential role in drug resistance. Discover Oncol. 2025;16(1):392. 10.1007/s12672-025-02106-0. 10.1007/s12672-025-02106-0 PMC11937470 40131660

[346] Wei J, Liu F, Lu Z, Fei Q, Ai Y, He PC, et al. Differential m(6)A, m(6)A(m), and m(1)A Demethylation Mediated by FTO in the Cell Nucleus and Cytoplasm. Mol Cell. 2018;71(6):973-85 e5. 10.1016/j.molcel.2018.08.011. 30197295 10.1016/j.molcel.2018.08.011 PMC6151148

[347] Siravegna G, Marsoni S, Siena S, Bardelli A. Integrating liquid biopsies into the management of cancer. Nat Rev Clin Oncol. 2017;14(9):531–48. 10.1038/nrclinonc.2017.14. 28252003 10.1038/nrclinonc.2017.14

